# Halide Perovskites and Their Derivatives for Efficient, High‐Resolution Direct Radiation Detection: Design Strategies and Applications

**DOI:** 10.1002/adma.202304523

**Published:** 2023-12-06

**Authors:** Kavya Reddy Dudipala, Thanh‐Hai Le, Wanyi Nie, Robert L. Z. Hoye

**Affiliations:** ^1^ Inorganic Chemistry Laboratory University of Oxford Oxford OX1 3QR UK; ^2^ Center for Integrated Nanotechnologies Los Alamos National Laboratory Los Alamos NM 87545 USA

**Keywords:** charge‐carrier kinetics, halide perovskites, imaging, ion migration, perovskite‐inspired materials, radiation detectors, stability

## Abstract

The past decade has witnessed a rapid rise in the performance of optoelectronic devices based on lead‐halide perovskites (LHPs). The large mobility‐lifetime products and defect tolerance of these materials, essential for optoelectronics, also make them well‐suited for radiation detectors, especially given the heavy elements present, which is essential for strong X‐ray and γ‐ray attenuation. Over the past decade, LHP thick films, wafers, and single crystals have given rise to direct radiation detectors that have outperformed incumbent technologies in terms of sensitivity (reported values up to 3.5 × 10^6^ µC Gy_air_^−1^ cm^−2^), limit of detection (directly measured values down to 1.5 nGy_air_ s^−1^), along with competitive energy and imaging resolution at room temperature. At the same time, lead‐free perovskite‐inspired materials (e.g., methylammonium bismuth iodide), which have underperformed in solar cells, have recently matched and, in some areas (e.g., in polarization stability), surpassed the performance of LHP detectors. These advances open up opportunities to achieve devices for safer medical imaging, as well as more effective non‐invasive analysis for security, nuclear safety, or product inspection applications. Herein, the principles behind the rapid rises in performance of LHP and perovskite‐inspired material detectors, and how their properties and performance link with critical applications in non‐invasive diagnostics are discussed. The key strategies to engineer the performance of these materials, and the important challenges to overcome to commercialize these new technologies are also discussed.

## Graphical abstract

Lead‐halide perovskites, and their bismuth‐based derivatives, have given rise to X‐ray detectors that perform orders of magnitude better than incumbent technologies. Herein, the principles behind these rapid improvements in performance are discussed, along with the challenges for the commercialization of these new technologies, and their wider impact in room‐temperature, high‐resolution particle detection.

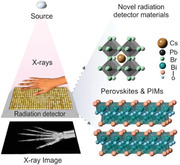

## 1 Introduction

Over the past decade, lead‐halide perovskites (LHPs) have come to dominate the emerging optoelectronic materials scene, with rapid rises in efficiency in photovoltaics and light‐emitting diodes.^[^ ^1^, ^2^, ^3^, ^4^, ^5^ ^]^ The unprecedented rate of development of these new halide perovskite technologies arises from a combination of the exceptional optoelectronic properties (including high photoluminescence quantum yields, sharp absorption onsets, and strong optical absorption, as well as long charge‐carrier transport lengths) and compatibility with facile solution and vapor‐based processing techniques.^[^ ^6^, ^7^, ^8^, ^9^, ^10^, ^11^, ^12^, ^13^ ^]^ At the same time, the successes of the lead‐halide perovskites, as well as their limitations in terms of toxicity and stability,^[^ ^14^ ^]^ have led to the search for nontoxic, air‐stable alternatives that could mimic their exceptional optoelectronic properties, especially tolerance to point defects, which is considered to be key to allowing efficient devices to be made using cost‐effective processing methods.^[^ ^15^, ^16^, ^17^, ^18^, ^19^ ^]^ Such efforts have focused primarily on compounds based on heavy post‐transition metal cations (namely Bi^3+^, Sb^3+^, and Sn^2+^).^[^ ^20^, ^21^, ^22^, ^23^ ^]^ These compounds are termed ‘perovskite‐inspired’ materials (PIMs) because they are either chemically (e.g., tin perovskites),^[^ ^24^ ^]^ structurally (e.g., halide elpasolites, such as Cs_2_AgBiBr_6_)^[^ ^25^ ^]^ or electronically (*e.g*., sodium bismuth sulfide)^[^ ^21^ ^]^ analogous to LHPs. In a number of cases (*e.g*., for BiI_3_ and SbSeI), the compounds explored were originally investigated for radiation detection.^[^ ^16^, ^26^, ^27^ ^]^ Over the past decade, there has been a growing shift in the efforts from the community working on LHPs and PIMs back from their newfound focus on photovoltaics to encompass radiation detectors again, owing to the rapid improvements in sensitivity and limit of detection.^[^ ^28^, ^29^ ^]^

**Figure 1:**
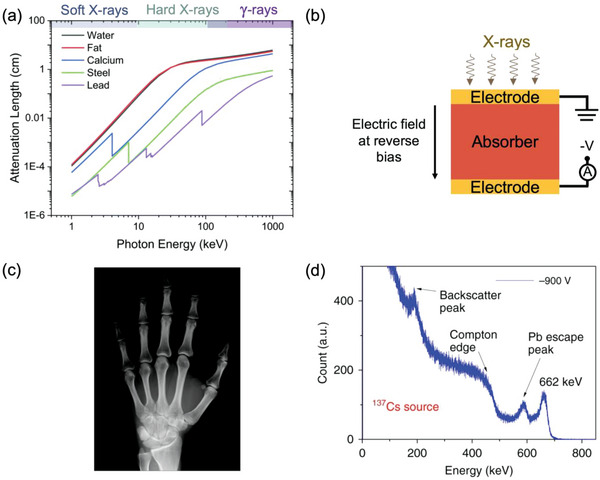
Application of ionizing radiation for non‐invasive, non‐destructive diagnostics. a) Illustration of the attenuation of common materials to electromagnetic radiation, with the regions categorized as soft and hard X‐rays, and γ‐rays. Reproduced under the terms of the CC‐BY‐3.0 license.^[^ ^31^ ^]^ Copyright 2021, The Authors. Published by the Royal Society of Chemistry. b) Sketch of a direct radiation detector (vertical photoconductor structure) operated under reverse bias.^[^ ^43^ ^]^ c) Example of an image of a hand taken using X‐rays and an amorphous selenium detector. Reproduced under the terms of the CC‐BY license.^[^ ^44^ ^]^ Copyright 2011, The Authors. Published by MDPI. d) Example of a γ‐ray spectrum taken with a CsPbBr_3_ single crystal detector, showing the resolution of the 662 keV ^137^Cs peak. Reproduced under the terms of the CC‐BY license.^[^ ^45^ ^]^ Copyright 2018, The Authors. Published by Springer Nature.

Ionizing radiation plays a powerful role in modern non‐invasive, non‐destructive diagnostic tools because of the highly penetrating nature of the high‐energy particles or electromagnetic waves used.^[^ ^30^, ^31^, ^32^, ^33^ ^]^ In particular, soft (0.1–10 keV) and hard (10–200 keV) X‐rays,^[^ ^34^ ^]^ as well as γ‐rays (0.1–100 MeV) are widely used,^[^ ^30^, ^32^, ^33^ ^]^ and can penetrate tissue and biological materials on the millimeter scale or longer (**Figure** **1**a).^[^ ^35^ ^]^ The attenuation of radiation is proportional to $Z_{\mathrm{av}}^{n}\;A^{-1}E^{-3}$, where $Z_{\mathrm{av}}$ is the average atomic number of the elements present in the material, 4 ≤ *n* ≤ 5 for photoelectric absorption,^[^ ^36^ ^]^ *A* the atomic mass and *E* the photon energy.^[^ ^28^ ^]^ As such, imaging with ionizing radiation allows for contrast of matter based on $Z_{\mathrm{av}}$ and *A*, and is therefore widely used in non‐invasive medical diagnostics (*e.g*., to image tissues and bones, see Figure 1c for an example image),^[^ ^37^, ^38^ ^]^ non‐destructive inspection of industrial goods (such as microprocessors),^[^ ^39^ ^]^ homeland security, oil exploration, and space exploration, among many other possibilities.^[^ ^31^, ^33^, ^40^ ^]^ Furthermore, ionizing radiation is widely used in materials research (*e.g*., crystallography, X‐ray photoemission spectroscopy, X‐ray absorption spectroscopy), astronomy,^[^ ^41^ ^]^ and the detection of radioactive waste through γ‐ray spectrometry.^[^ ^42^ ^]^

There are two common ways to detect ionizing radiation in the solid‐state: direct and indirect methods. The direct method involves absorbing radiation in a detector to generate electrons and holes, which are then extracted and the signal read (Figure 1c).^[^ ^30^ ^]^ In the indirect method, ionizing radiation is absorbed by a scintillator, which downconverts the energy to photons in the UV or visible wavelength range that are then collected by weak light sensors. Detailed reviews on indirect detectors can be found in several sources, including Ref. [30, 31, 32, 33, 40] which also go into detail on the mechanism behind indirect detectors, and the properties of the scintillator materials required. Indirect detectors are advantageous because they decouple the attenuation of radiation and the generation of an electronic signal into two steps. This means that there is no longer a requirement to collect the charge‐carriers generated through radiation absorption, and the radiation attenuation material and electronics can be optimized separately, such that novel scintillators can still make use of the mature photodetectors that have undergone decades of advancement.^[^ ^31^ ^]^ On the other hand, direct detectors are advantageous by typically giving higher spatial resolution in imaging than indirect detectors (although emerging lead‐halide perovskite scintillators are challenging this position—see Section 5.5 and Table 4),^[^ ^46^ ^]^ and are more accurate in measuring the energy of incident radiation.^[^ ^45^ ^]^ Furthermore, in the direct approach, there is no need for the material to have a high light yield, making it highly suitable for perovskite‐inspired materials, which strongly attenuate ionizing radiation, but which, in many cases, do not strongly luminesce. This Review will focus on direct solid‐state detectors.

The current state‐of‐the‐art materials used industrially for direct detectors are primarily amorphous selenium (α‐Se), cadmium zinc telluride (Cd_1‐_ *_x_* Zn*_x_* Te or CZT), silicon and high‐purity germanium (HPGe).^[^ ^30^ ^]^ However, these materials face challenges due to limited performance, scalability, compatibility with flexible, curved substrates, and/or growing sufficiently large crystals cost‐effectively.^[^ ^30^, ^47^ ^]^ In particular, the dose rate of radiation required for medical imaging is limited by how effectively they can be detected, and the current medical standard dose rate for X‐rays is 5500 nGy_air_ s^−1^.^[^ ^48^, ^49^ ^]^ This is four orders of magnitude above background levels of X‐rays (0.1 nGy_air_ s^−1^),^[^ ^30^ ^]^ and a standard computed tomography (CT) scan exposes the patient to ionizing radiation equivalent to 2 years’ worth of natural radiation exposure.^[^ ^50^ ^]^ It has been estimated that approximately 2% of cancers diagnosed in the United States in 2007 could be linked to CT scans.^[^ ^50^ ^]^ To improve the safety of medical imaging, as well as to improve the effectiveness of a wide range of other diagnostics and characterization involving ionizing radiation, it is essential to develop new materials capable of detecting lower dose rates of radiation, which are cost‐effective to manufacture.

**Figure 2:**
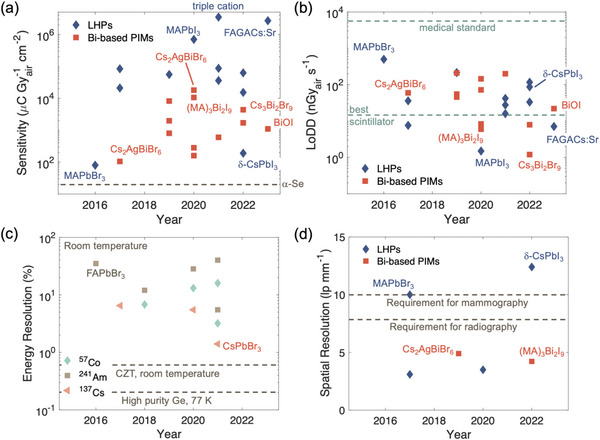
Rise in performance of lead‐halide perovskite (LHP) and Bi‐based perovskite‐inspired material (PIM) radiation detectors. Plot of a) sensitivity and b) lowest detectable dose rate (LoDD) reported for X‐ray detectors based on single crystal LHPs and Bi‐based PIMs. The sensitivity of commercial‐standard α‐Se is shown (Tables 1 and 3), as is the LoDD of α‐Se (the current medical standard, 5500 nGy_air_ s^−1^)^[^ ^48^, ^49^ ^]^ and the best LoDD of scintillators (13 nGy_air_ s^−1^).^[^ ^51^ ^]^ c) Energy resolution of LHP single crystal detectors for 122 keV ^57^Co, 59.6 keV ^241^Am and 667 keV ^137^Cs γ‐rays,^[^ ^52^ ^]^ compared to the energy resolution of high purity Ge at 77 K (0.2%) and CZT at room temperature (0.5%).^[^ ^30^ ^]^ d) Spatial resolution (at 20% modulation transfer function) of LHPs and Bi‐based PIM X‐ray imagers, compared with the minimum requirements for radiography (5.7 lp mm^−1^) and mammography (10 lp mm^−1^).^[^ ^53^ ^]^ Please refer to Table 4 for a more detailed discussion of the evolution of spatial resolution in LHP imagers. Data obtained from Ref. [54] Higher sensitivity and spatial resolution are better, while lower LoDD and energy resolution are better. Please note that the reports shown were not all obtained under the same conditions (including dose rate, mean radiation energy, spectrum of radiation source, applied bias). Since each of these parameters influences the performance of radiation detectors, we cannot obtain a direct comparison between each report. But this figure serves as a useful visual guide of what values have been reported. Readers are encouraged to go to Table 3, and the references therein to check the measurement conditions used.

In the decade of active work on radiation detectors based on LHPs and PIMs, these devices have already achieved the lowest detectable dose rates (LoDDs) over two orders of magnitude below the current medical standard for X‐ray imaging, with orders of magnitude higher sensitivities. The astonishing rise in performance of this class of radiation detectors is highlighted in **Figure** **2**. For radiation detector materials currently used commercially, a common drawback of the direct detection of radiation concept is that a thick detector is needed to adequately absorb high‐energy γ‐ray radiation, such that it is difficult to extract all charge‐carriers generated.^[^ ^31^ ^]^ However, Pb and Bi are the two heaviest elements that do not undergo radioactive decay, such that LHPs and PIMs have stopping powers nearly double that of CdTe in the hundreds of keV photon energy range. Furthermore, cm‐scale single crystals of lead‐halide perovskites and Bi‐based compounds (*e.g*., BiI_3_) can be grown, such that there is sufficient material to attenuate γ‐rays.^[^ ^30^ ^]^ As a result, an energy resolution of 1.4% has been achieved with direct CsPbBr_3_ detectors,^[^ ^55^ ^]^ and 2.2% for Sb‐doped BiI_3_ for 662 keV ^137^Cs γ‐rays.^[^ ^56^ ^]^

This Review discusses the principles behind the rapid rise in performance of direct radiation detectors based on LHPs and PIMs, and the potential impact on non‐invasive diagnostics. We begin by discussing key applications and their requirements, especially the materials properties needed to achieve high performance. Next, we discuss how these properties can be met by the major classes of halide perovskites and their derivatives, and the important strategies for engineering their performance. This includes the interplay between composition, dimensionality, defects, and interfaces on performance, and the role of carrier‐phonon coupling, and how this could be controlled. Finally, we discuss the pressing challenges to address to commercialize these detectors, and the important future challenges of this technology. We hope that this Review will draw in the wider community working on metal‐halide semiconductors towards efforts at developing more effective radiation detectors, and equip them with the critical principles and strategies to push the boundaries in performance. We also highlight important challenges around the inconsistency in measuring and reporting detector and imager performance, and hope to advance the field towards standardization, which will be critical as the field continues to grow.

## 2 Properties Required for Effective Radiation Detection

In this section, we will discuss the key applications for radiation detectors, the requirements for each application and important performance metrics, and how the detector materials could be optimized to maximize each metric. In particular, we emphasize the limitations of incumbent technologies for each application, and the areas in which lead‐halide perovskites and their derivatives could make an impact. We also highlight the limitations of some of the current practices in measuring detector performance, especially the effects of photoconductive gain.

### 2.1 Applications

**Figure 3:**
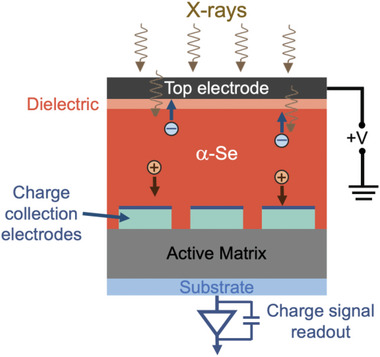
Simplified diagram of a typical direct‐conversion Flat Panel X‐ray Imager based on α‐Se as the X‐ray attenuation layer. Diagram based on Ref. [57] Here, we discuss the attenuating materials used for digital radiography, and the key properties required. A discussion of the interfaces, and how they influence performance and polarization stability is given in Sections 4.6 and 5.3.

Digital radiography has had a profound impact on medical imaging and non‐invasive screening, enabling improved precision, faster image processing, greater ease of image transmission over communication networks, and increased flexibility of display possibilities compared to traditional film‐based imaging.^[^ ^39^, ^57^, ^58^ ^]^ Central to these advances was the development of Flat Panel X‐ray Imagers (FPXIs), which, for direct detectors, was comprised of an X‐ray absorber deposited over and electronically integrated with an active matrix (AM) array of electronic components,^[^ ^57^ ^]^ namely capacitors and thin film transistor switches,^[^ ^57^ ^]^ as shown in **Figure** **3**. α‐Se (typically 500 µm thickness) and hydrogenated amorphous silicon (a‐Si:H) have been the favored materials for the X‐ray photoconductor and AM array, respectively, because they can be deposited cost‐effectively over a large area.^[^ ^57^, ^59^ ^]^ It is important that the detectors used in digital radiography can record transmitted X‐rays over the entire area of the object investigated, with common examples in chest radiography (35 cm × 43 cm) or mammography (18 cm × 24 cm, or 24 cm × 30 cm).^[^ ^39^ ^]^

α‐Se direct FPXIs are advantageous over indirect FPXIs by having a higher detective quantum efficiency, modulation transfer function, and image contrast, enabling the imaging of fine anatomic structures.^[^ ^59^ ^]^ However, the low atomic number of Se (34) limits the stopping power for radiation, such that α‐Se FPXIs are more suited to the detection of lower‐energy radiation (such as in mammography, **Table** **1**).^[^ ^58^, ^60^ ^]^ By contrast, computed tomography (CT), and other high‐energy radiation applications, typically uses scintillator detectors.^[^ ^59^ ^]^ CdTe and CZT are also commercial direct‐detector materials, and are advantageous over α‐Se by having a higher atomic number (48–52) and mobility‐lifetime (*µτ*) product of 10^−5^–10^−2^ cm^2^ V^−1^, compared to 10^−7^ cm^2^ V^−1^ for α‐Se.^[^ ^61^, ^62^, ^63^, ^64^, ^65^ ^]^ As a result, higher sensitivities have been achieved with CZT detectors (typically 318–2400 µC Gy_air_^−1^ cm^−2^ in polycrystalline materials,^[^ ^66^ ^]^ compared to 20 µC Gy_air_^−1^ cm^−2^ for α‐Se).^[^ ^61^ ^]^ But we note that there are reports of CZT detectors with substantially higher sensitivities reaching >10^4^ µC Gy_air_^−1^ cm^−2^ (see Table 1), although this came about through the effects of photoconductive gain that may have been accentuated by charge‐injection from the electrodes, as well as trapping effects. However, the high photon fluxes used in CT, as well as the requirements for fast data acquisition, make CT applications still a significant challenge for CdTe and CdZnTe detectors, and remains the subject of intense research efforts.^[^ ^58^ ^]^ Whilst CZT detectors have been used for low‐energy astronomy applications (Table 1),^[^ ^31^ ^]^ their spectroscopic performance and time resolution remain poor compared to scintillators for γ‐ray applications.^[^ ^67^ ^]^ Thus, scintillators remain the detectors of choice for positron emission tomography, which is becoming an increasingly more commonplace medical imaging technique and involves the emission of 511 keV γ‐ray photons when an emitted positron annihilates with an electron.^[^ ^67^ ^]^ At the same time, Ge has found uses in γ‐ray spectroscopy, but requires expensive processing to achieve high purities with low structural defect densities, and needs to be operated at cryogenic temperatures (Table 1).^[^ ^68^ ^]^ New classes of high‐stopping power radiation detectors are therefore urgently needed that can overcome the limitations of incumbent direct detectors, but which can still be manufactured cost‐effectively into large‐area detectors.^[^ ^31^ ^]^ It is especially important to develop detectors with lower detection limits, since established materials for both indirect and direct detection are not expected to have further substantial improvements in this metric.^[^ ^59^ ^]^

**Table 1:** Important applications of direct radiation detectors, typical materials, their performance and key detector requirements

Application	Energy range [keV]	Typical material	Current performance				Key requirements
			Sensitivity [µC Gy_air_^−1^ cm^−2^]	LoDD [nGy_air_ s^−1^]		Energy resolution [%]
				Extrapolated	Direct
Computed tomography (medical)	80 – 410	CdWO_4_ (scin‐tillator)	n/a	n/a	n/a	n/a	Detectors need to be stable under high flux X‐rays ^[^ ^58^ ^]^ Very fast‐response detectors
Mammography (medical)	20 – 40	α‐Se	20 ^[^ ^61^ ^]^	n/a	5500	n/a	Hundreds of microns spatial resolution to detect tumors and microcalcifications^[^ ^31^ ^]^
Digital radiography (medical)	60 – 120	α‐Se	20 ^[^ ^58^ ^]^	n/a	5500	n/a	Photoelectric conversion efficiency >30 e^−^ keV^−1^ Capable of resolving 1 frame s^−1^ (static) or 30 frames s^−1^ (dynamic) Dark current density <10^−10^ A cm^−2 [^ ^69^ ^]^
Astronomy	3 – 79 (NuSTAR)	CZT	15,200 ^[^ ^65^ ^]^	n/a	<14.3^[^ ^65^ ^]^	0.5 ^[^ ^30^ ^]^	Energy resolution of ≈1.5% at 60 keV
γ‐ray spectrometry (nuclear security)	10‐10000	HPGe (77 K)	n/a	n/a	n/a	0.2 ^[^ ^68^ ^]^	Energy resolution <7% at 662 keV to resolve isotopes Low‐cost and scalable

### 2.2 Device Requirements

When electromagnetic radiation interacts with matter, the types of interactions that occur and particles formed depend on the energy of the incident radiation.^[^ ^33^ ^]^ For radiation up to the hundreds of keV in energy, the dominant process is photoelectric absorption, in which the incident photon ionizes atoms, giving rise to a primary electron and an inner shell hole.^[^ ^30^, ^33^ ^]^ The average electron‐hole pair creation energy (also referred to as the ionization energy), Δ, was found empirically to be related to the bandgap (*E*_g_) by Equation 1.^[^ ^70^ ^]^

(1) $$\Delta =2.00E_{g}+1.43$$

It can be seen from Equation 1 that photoelectron generation is more difficult in materials with a wider bandgap due to a higher electron‐hole pair creation energy. At the same time, a wider bandgap is favorable for reducing the dark current, which is needed to achieve a high sensitivity and low lowest detectable dose rate (refer to the next two sub‐sections). Typically, bandgaps between 1.4 and 2.5 eV are considered ideal for radiation detectors.^[^ ^70^, ^71^ ^]^

Following ionization, the energetic photoelectron loses kinetic energy to generate multiple electron‐hole pairs through inelastic electron‐electron scattering, secondary X‐ray generation, and Auger processes. These electrons and holes subsequently drift to opposite electrodes via an electric field gradient, where they are collected as a photocurrent.^[^ ^30^, ^36^ ^]^ For medical imaging, these detectors are typically operated in current mode, in which the detector is connected to an ammeter circuit with a slow response time, such that the current produced is an average across all radiation pulses, and is proportional to the intensity of the incident radiation.^[^ ^30^ ^]^ For spectroscopy, detectors are typically operated in pulse mode, where there is a relatively weak photon flux intensity (as low as 1 photon cm^−2^ s^−1^, or smaller),^[^ ^72^ ^]^ such that each incident photon produces a set of electron‐hole pairs, which is measured as a current pulse. The number of electron‐hole pairs is proportional to the incident energy, allowing a histogram to be generated that reflects the spectrum of the incident radiation.^[^ ^30^ ^]^ Apart from photoelectric absorption, there is also Compton scattering (involving the transfer of only a small fraction of energy from the radiation to the atoms) or electron‐positron pair formation, but these occur for radiation with energy greater than a few hundred keV, or >1.02 MeV, respectively.^[^ ^33^ ^]^

#### 2.2.1 High Sensitivity

Sensitivity, *S*, is one of the most important figures of merit for radiation detectors, and is defined as the charge, *Q*, collected per unit exposure to radiation (*D*, in grays per second; Gy_air_ s^−1^, where 1 Gy_air_ = 1 J kg^−1^) per unit area (*A*), as given in Equation 2.^[^ ^33^ ^]^

(2) $$S=\left(I_{\mathrm{light}}-I_{\mathrm{dark}}\right)/DA$$

In Equation 2, *I*_light_ is the total current extracted under illumination (photocurrent plus dark current) and *I*_dark_ the total current in the dark. Typically, the photocurrent, *I*_light_ – *I*_dark_ would be averaged across several light/dark cycles as the radiation source is chopped. Increasing the dose rate should ideally produce a linear increase in photocurrent, but this is not always the case in novel systems.^[^ ^54^ ^]^

Sensitivity is an important parameter because a high sensitivity enhances the dynamic range achievable with the detector, with lower radiation exposure to the patient.^[^ ^73^ ^]^ The sensitivity depends on several material parameters, namely: i) the mass attenuation coefficient, *µ*_ac_, ii) mobility‐lifetime (*µτ*) product, iii) charge‐collection efficiency, and iv) leakage current.^[^ ^33^ ^]^

A high mass attenuation coefficient, *µ*_ac_, is necessary for high‐energy radiation to be absorbed within a depth that is small enough for there to be a high efficiency of charge‐carrier extraction. The photon flux of transmitted radiation (*I*) is given by Equation 3.^[^ ^30^ ^]^

(3) $$I=I_{0}\exp\left[-\mu_{\mathrm{ac}}\;\rho \;l\right]$$

In Equation 3, *I*_0_ is the incident photon flux of radiation, *ρ* the mass density of the material, and *l* the interaction length. It can be seen that materials with high *Z*_av_ and therefore high *µ*_ac_ are needed for strong radiation attenuation. Materials are also referred to by their stopping power, which is the average energy dissipated by ionization radiation per unit length of travel.^[^ ^74^ ^]^

The drift length is given by the product between *µτ* and the electric field (*E*) within the active channel. Thus, a high *µτ* product is necessary for charge‐carriers to be extracted from the large interaction volume within which ionizing radiation is absorbed. The *µτ* product is typically measured by fitting the modified Hecht equation to the measured photocurrent (*I*_ph_), as given in Equation 4.^[^ ^30^ ^]^

(4) $$\frac{I_{\mathrm{ph}}}{I_{0}}=\frac{\mu \tau V}{L^{2}}\;\frac{1-\exp\left(-\frac{L^{2}}{\mu \tau V}\right)}{1+\frac{Ls}{V\mu }}$$

**Figure 4:**
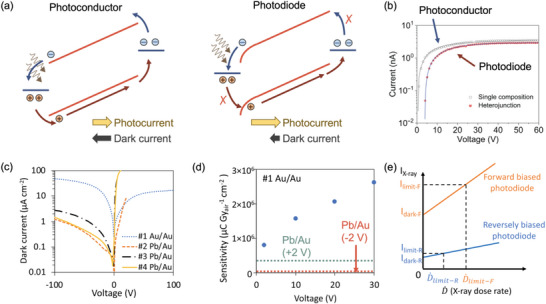
Effect of device architecture on photocurrent, and the performance metrics of radiation detectors. a) Comparison of the band diagram of photoconductors (left – symmetric Ohmic electrodes) and photodiodes (right – Schottky contact on the left, and Ohmic contact on the right) placed in reverse bias. In both cases, X‐rays are illuminated through the electrode on the left. For the photoconductor, charge‐carriers can be injected from the electrodes into the semiconductor, such that dark current can match or exceed the photocurrent. For the photodiode, the electrodes impede or block charge‐injection from the electrodes, such that the photocurrent substantially exceeds the dark current. b) Plot of photocurrent against applied bias for perovskite radiation detectors made into the photoconductor and photodiode structure. Reproduced under the terms of the CC‐BY license.^[^ ^75^ ^]^ Copyright 2021, The Authors. Published by Elsevier. c) Comparison of the dark currents for perovskite single crystal devices made with symmetric (Au/Au) or asymmetric (Pb/Au) electrodes, d) the sensitivities of Au/MAPbI_3_/Au *vs*. Pb/MAPbI_3_/Au devices under forward and reverse bias, and e) the lowest detectable dose rate (${\dot{D}}_{\mathrm{limit}}$) that would be extracted from the Pb/MAPbI_3_/Au device under reverse (photoconductive gain < 1) or forward bias (photoconductive gain > 1). Panels (c)–(e) reproduced under the terms of the CC‐BY license.^[^ ^43^ ^]^ Copyright 2021, The Authors. Published by Springer Nature.

In determining the *µτ* product from this model, it is assumed that i) photoelectrons are only generated in a narrow region next to one electrode (and not throughout the whole channel), and ii) the electrodes used are blocking, such that only charge‐carriers generated through ionization are extracted.^[^ ^75^ ^]^ Under such conditions, *I*_ph_ will increase with increasing applied field with a decreasing rate, and will eventually saturate. In Equation 4, *I*_0_ is the saturated photocurrent, *V* is the applied voltage, *L* is the length of the channel, and *s* is the surface recombination velocity.^[^ ^75^ ^]^ Assumption (i) can be fulfilled by illuminating the device through one electrode, and ensuring that the channel length is significantly larger than the absorption depth (**Figure** **4**a). Regarding assumption (ii), it is common historically to use symmetric electrodes and rely on the applied field to selectively extract either electrons or holes (Figure 4a).^[^ ^70^ ^]^ More recently, this practice has been adopted in the measurement of the *µτ* product of novel semiconductors, with Au a common electrode material.^[^ ^51^, ^76^, ^77^, ^78^, ^79^ ^]^ However, in using two Ohmic contacts, the assumption of blocking electrodes is not fulfilled, and charges are injected into the channel (Figure 4a, left). Thus, groups working on lead‐halide perovskites have adopted the charge‐selective contacts used in perovskite solar cells, or the use of one Schottky contact and one Ohmic contact and ensuring that the dark current is orders of magnitude below the photocurrent under reverse bias (Figure 4a, right).^[^ ^80^, ^81^ ^]^ It can be seen from Figure 4b that the photocurrent profile, and thus the *µτ* product that would be extracted by applying the modified Hecht model, would be different depending on whether the photoconductor or photodiode structure is used.

The charge collection efficiency (CCE) is defined as the ratio of the electrical charge generated in the external circuit (*Q*) to the charge generated by the incident radiation in the detector (*Q*_0_), and this can be calculated using the Hecht model, as given by Equation 5.^[^ ^33^ ^]^

(5) $$\begin{array}{ccc} \mathrm{CCE} & = & \frac{\mu_{h}\tau_{h}E}{L}\left(1-\exp\left[-\frac{x}{\mu_{h}\tau_{h}E}\right]\right)\\ & & +\;\frac{\mu_{e}\tau_{e}E}{L}\left(1-\exp\left[-\frac{\left(L-x\right)}{\mu_{e}\tau_{e}E}\right]\right) \end{array}$$

In Equation 5, *x* is the position from the electrode that the ionizing radiation is incident on. The other parameters are the same as in Equation 4, and the *µτ* product for holes (h) and electrons (e) are specified. From this equation, it can be seen that larger CCEs can be achieved with higher *µτ* products and shorter transport distances. Shorter transport distances can be achieved when the stopping power is higher, such that less material is needed to reach a desired attenuation efficiency.

Higher CCEs can be obtained by reducing leakage currents. This can be achieved by having a wider bandgap in the detector material (thus lowering the population of thermally‐generated charge‐carriers), minimizing defects, and compensating doping to obtain an intrinsic semiconductor (thus minimizing the background charge‐carrier population), as well as minimizing ionic conduction. In other words, achieving high CCEs requires a high resistivity, typically >10^9^ Ω cm.^[^ ^71^ ^]^

Finally, although high sensitivities >10^4^ µC Gy_air_^−1^ cm^−2^ have been reported in LHP X‐ray detectors (Figure 2a), it is important to note that these high values can arise in part as a consequence of photoconductive gain.^[^ ^82^ ^]^ Photoconductive gain occurs when the photocurrent obtained is amplified by a factor *G*, such that the ratio of electrons extracted to photons input exceeds 100% per unit time, and thereby causes the sensitivity to exceed the theoretical limit. The factor *G* can be described by Equation 6.^[^ ^47^, ^83^, ^84^ ^]^

(6) $$G=\frac{\tau_{r}}{\tau_{t}}=\frac{\mu V\tau_{r}}{L^{2}}$$

**Figure 5:**
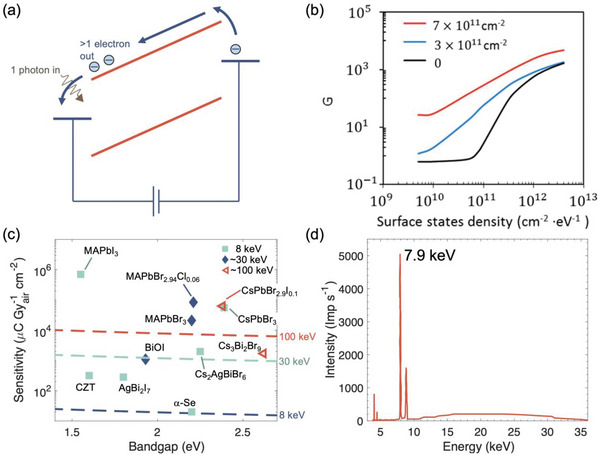
Effects of photoconductive gain, and the sensitivity of X‐ray detectors. a) Illustration of the conventional explanation for photoconductive gain. Only electrons shown for convenience. b) Plot of photoconductive gain (*G*) against surface state density for a simulated *p*‐type silicon slab (400 nm thick) for different concentrations of fixed charges. Part (b) reproduced with permission.^[^ ^85^ ^]^ Copyright 2018, American Chemical Society. c) Theoretical maximum sensitivity (*S*_0_) calculated from Equation 7 at different X‐ray energies (8 keV, 30 keV, 100 keV) as a function of the bandgap of the attenuation material, shown as dashed lines. The reported sensitivities of lead‐halide perovskite and Bi‐based PIM X‐ray detectors are shown as points, organized based on the approximate peak energy of the X‐ray source. Data shown in Table 3. d) X‐ray spectrum used to measure BiOI detectors, showing that whilst the peak energy is at 7.9 keV, the spectrum had photons at up to 35 keV energy. Part (d) reproduced with permission under the terms of the CC‐BY license.^[^ ^56^ ^]^ Copyright 2023, The Authors. AIP Publishing.

In Equation 6, τ_r_ is the recombination time of charge‐carriers localized in trap states, and τ_t_ the transit time of the free charge‐carriers, which can be described by the charge‐carrier mobility (µ), applied bias (*V*), and distance between the two electrodes (*L*), assuming the electric field to be uniform.^[^ ^47^, ^83^ ^]^ Equation 6 is based on the model that photoconductive gain arises because the minority charge‐carrier is trapped and accumulates before eventually recombining with the free majority carriers (**Figure** **5**a).^[^ ^47^ ^]^ As a result, photogenerated charge‐carriers can circulate through the device multiple times before recombining with the trapped carriers, giving the impression that there are more photogenerated carriers output than photons input.^[^ ^85^ ^]^ However, Dan et al. have argued that a photoconductor (e.g., LHP with two Ohmic Au electrodes) has no gain, or at least no significant gain, and that the perceived gain arises due to the spatial localization of excess minority carriers, along with the accumulation of majority carriers in the conducting channel. This gives the impression of photoconductive gain.^[^ ^85^ ^]^ That, is as the concentration of surface, interface, or bulk traps increases, and one carrier type is preferentially trapped, there is an increasing difference between the excess carrier density of electrons and holes, leading to a larger perceived gain (Figure 5b).^[^ ^85^ ^]^

Regardless of the mechanism behind photoconductive gain, the maximum theoretical limit in sensitivity (*S*_0_ in µC Gy_air_^−1^ cm^−2^) in the absence of photoconductive gain is given by Equation 7.^[^ ^52^, ^61^ ^]^

(7) $$S_{0}=\left(\frac{6.21\times 10^{21}q}{\left(\alpha_{\text{air}}/\rho_{\text{air}}\right)\Delta }\right)\left(\frac{\alpha_{\text{en}}}{\alpha }\right)$$

In Equation 7, *q* is the charge of an electron (in C), α_air_ the energy absorption coefficient of air (in cm^−1^), ρ_air_ density of air (in g cm^−3^), Δ the mean energy required to form an electron‐hole pair (in eV; see Equation 1), α_en_ the energy absorption coefficient of the photoconductor material, and α its linear attenutation coefficient.^[^ ^52^, ^61^ ^]^ The maximum value of $\frac{\alpha_{\mathrm{en}}}{\alpha }$ is unity, and thus *S*_0_ will vary with 1) ∆ between different materials, and 2) $\alpha_{\text{air}}/\rho_{\text{air}}$ for different radiation energies (e.g., $\alpha_{\text{air}}/\rho_{\text{air}}$ is 9.446 cm^2^ g^−1^ at 8 keV, 0.1537 cm^2^ g^−1^ at 30 keV, or 0.0233 cm^2^ g^−1^ at 100 keV).^[^ ^86^ ^]^ A plot of *S*_0_ against bandgap for common perovskite materials is given in Figure 5c. As can be seen, the reported sensitivities in many cases significantly exceed this theoretical limit. To a certain extent, this can occur because although the X‐ray energy is quoted at one value, this is usually the energy at which the X‐ray intensity is at a maximum, whereas the X‐ray source is a spectrum of a wide range of X‐ray energies (see for example Figure 5d). The sensitivity could then reach values exceeding the *S*_0_ calculated based on the energy of the X‐ray peak. At the same time, there are sensitivities reaching >10^4^ µC Gy_air_^−1^ cm^−2^ (Figure 5c), going well beyond the *S*_0_ for 100 keV X‐rays, and photoconductive gain likely plays a role. While photoconductive gain may give rise to a higher sensitivity and responsivity, it can also make the dark current and noise higher, and therefore does not enhance the capability of X‐ray detectors to lower the dose rate required for medical imaging.^[^ ^80^ ^]^ Thus, the more important parameters are the limit of detection (see next sub‐section), dark current stability, and dynamic behavior.^[^ ^87^ ^]^ Indeed, lower sensitivities can be compensated for by using an external amplifier.^[^ ^87^ ^]^

An important factor that influences photoconductive gain and the sensitivity measured is the device architecture used. Halide perovskites have been investigated as direct radiation detectors in the photoconductor, photodiode, and phototransistor device architectures. Usually, photodiodes have a vertical device structure where the perovskite absorber layer is sandwiched between electrodes on the top and bottom. By contrast, phototransistors have a lateral device structure with co‐planar electrodes on the perovskite absorber.^[^ ^88^, ^89^ ^]^ Photoconductors can either be fabricated in a vertical or lateral device structure, depending on the charge‐carrier transport properties and dark current along the crystallographic planes present in the attenuation material between the electrodes.^[^ ^54^ ^]^ **Table** **2** presents a comparison of halide perovskite direct X‐ray detectors in these three device architectures.

**Table 2:** Comparison of halide perovskite direct X‐ray detectors in vertical (V) and lateral (L) type photoconductor, photodiode and phototransistor device architectures

Material	Device type	Dose rate used for sensitivity measurement [nGy_air_ s^−1^]	Sensitivity [µC Gy_air_^−1^ cm^−2^]	LoDD [nGy_air_ s^−1^]	X‐ray peak energy	Reference
CsPbBrI_2_	Phototransistor (L)	1‐10^7^	10^7^	1	20 keV	[92]
CsPbBr_2.9_I_0.1_	Photoconductor (V)	12.5‐30 × 10^3^	6.27 × 10^4^	117	120 keV	[95]
CsPbBr_3_	Phototransistor (L)	3.5 × 10^3^	1.2 × 10^9^	79	10‐60 keV	[89]
CsPbBr_3_	Photodiode (V)	0.35‐1.4 × 10^5^	55684	215	30 keV	[96]
CsPbBr_3_	Photodiode (V)	1‐7 × 10^3^	1256	n.r.	80 keV	[88]
CsPbBr_3_	Photoconductor (V)	0.15‐1.35 × 10^5^	61	n.r.	80 keV
CsPbBr_3_	Photoconductor (L)	17.3 × 10^3^	1450	n.r.	1‐2.5 keV	[97]
MAPbBr_3_	Photodiode (V)	1.2‐7 × 10^3^	529	1210	80 keV
MAPbBr_3_	Photodiode (V)	0.4‐4.5 × 10^3^	80	500	22 keV
MAPbBr_2.94_I_0.06_	Photodiode (V)	0.76‐4 × 10^3^	8.4 × 10^4^	7.6	8 keV
MAPbI_3_	Phototransistor (L)	3 × 10^5^	10^8^	n.r.	17.5 keV
MAPbI_3_	Photoconductor (L)	1‐10^4^	7.1 × 10^5^	1.5	50 keV
MAPbI_3_	Photoconductor (V)	0.2‐1.4 × 10^5^	968.9	n.r.	30 keV
MAPbI_3_	Photodiode (V)	7.46‐58.36 × 10^3^	9 × 10^3^	> 267	40 keV
MAPbI_3_	Photodiode (L)	1.5‐10^3^	5.2 × 10^6^	0.1	22 keV
FA_0.92_Cs_0.04_MA_0.04_PbI_3_	Phototransistor (L)	0.2‐2.9 × 10^5^	1.3 × 10^4^	7.84	40 keV
FA_0.85_MA_0.1_Cs_0.05_PbI_2.55_Br_0.45_	Photodiode (V)	7.46‐58.36 × 10^3^	3.5 × 10^6^	42	40 keV
FA_0.85_MA_0.1_Cs_0.05_PbI_2.55_Br_0.45_	Photoconductor (V)	7.46‐58.36 × 10^3^	2.1 × 10^6^	n.r.	40 keV
FAPbI_3_	Photodiode (V)	7.46‐58.36 × 10^3^	1.5 × 10^5^	> 267	40 keV

Cao and co‐workers compared a MAPbI_3_ perovskite single crystal made into a photodiode (i.e., with an Ohmic Au electrode on one side, and Schottky Pb electrode on the other) vs photoconductor (i.e., ohmic Au electrodes on both sides), as illustrated in Figure 4a.^[^ ^43^ ^]^ Under reverse bias, the photodiode architecture blocks charge injection, such that the photocurrent gain is always less than unity (Figure 4a, right). By contrast, the photodiode architecture under forward bias, or the photoconductor device under either forward or reverse bias will have charge‐injection from the electrodes and a photoconductive gain exceeding unity can occur, especially if the charge‐carrier lifetime exceeds the transit time under drift to the electrodes (Figure 4a, left). With photoconductive gain, more charges are extracted, leading to higher sensitivity values. For example, Pb/MAPbI_3_/Au under 2 V reverse bias has a sensitivity of 1.1 × 10^4^ µC Gy_air_^−1^ cm^−2^, whereas under 2 V forward bias increases to 3.5 × 10^5^ µC Gy_air_^−1^ cm^−2^, similar to the value for the Au/MAPbI_3_/Au device under 2 V forward bias (Figure 4d).^[^ ^43^ ^]^ At the same time, simply using a photodiode architecture does not guarantee that photoconductive gain is avoided. Jia et al. found that MAPbI_3_ detectors made in a photodiode architecture exhibited significant photoconductive gain, with external quantum efficiencies exceeding 100% due to ion accumulation at interfaces changing band bending, such that the diodes operated like photoconductors.^[^ ^90^ ^]^ It is therefore critical to account for the influence of photoconductive gain, and develop approaches that can minimize this effect.

On the other hand, in phototransistors, a high photoconductive gain can be attained while maintaining a low noise current, resulting in an excellent signal‐to‐noise ratio even with ultrathin (few‐nm thick) perovskite absorber layers.^[^ ^91^ ^]^ Phototransistors combine the functionalities of photoconductors and transistors, i.e. here, first the incident X‐ray photons are attenuated and charge‐carriers are generated in the absorber layer which results in an increased current density in the channel which is proportional to the intensity of incident X‐rays. Second, a gate voltage can be supplied to tune the conductivity of the channel. Thus, in phototransistors, the current density in the channel can not only be modulated by the incident X‐rays but also by supplying a gate voltage, which allows a controlled signal amplification without significant noise increment. This is beneficial for achieving both a high sensitivity and low detection limit simultaneously.^[^ ^89^, ^92^, ^93^ ^]^ Gao et al. employed a heterojunction phototransistor using CsPbBrI_2_ nanocrystals (∼50 nm thick layer) and PDPPBTT copolymer where the perovskite nanocrystals act as an attenuating medium and the copolymer acts as a conductive channel (Please refer to Ref. [92] for the full device structure). The charge transfer efficiency of photogenerated charge‐carriers from perovskite nanocrystals to the conductive channel depends on built‐in voltage and interface quality of CsPbBrI_2_/PDPPBTT heterojunction (Please refer to Section 4.6. for a detailed discussion on heterojunctions). Using this heterojunction phototransistor for the detection of 20 keV X‐rays, a high photoconductive gain of 10^8^ and low noise current of 10^−14^ A Hz^−1/2^ close to shot noise level was obtained.^[^ ^92^ ^]^ Owing to such high gain and low noise, a high sensitivity of 10^5^ µC Gy_air_^−1^ cm^−2^ and detection limit of 1 nGy_air_ s^−1^ were achieved simultaneously.

#### 2.2.2 Low Limit of Detection

The LoDD (also referred to as the detection limit, or limit of detection) of radiation detectors is commonly considered to be the radiation dose rate that gives a detector signal‐to‐noise ratio (SNR) of 3.^[^ ^43^, ^94^ ^]^ The SNR is commonly taken as the average photocurrent signal divided by the standard deviation in the photocurrent, and the LoDD determined by repeatedly measuring the SNR at different dose rates.^[^ ^78^, ^79^ ^]^ However, this approach is limited by fluctuations in the X‐ray intensity, which may lead to lower SNR values (and therefore higher LoDD values) than if the noise of the device only were considered.^[^ ^78^, ^79^ ^]^ This fluctuation in the light current typically exceeds that of the dark current, which strictly speaking should be the one used in determining the LoDD.^[^ ^79^ ^]^ An alternative approach has been proposed by Cao and co‐workers, which was derived based on statistical analysis. In this approach, the LoDD ($\dot{D}$) is calculated from Equation 8.^[^ ^79^ ^]^

(8) $$\dot{D}=I_{\mathrm{limit}}/A.S=3.29\sigma_{\mathrm{dark}}/A.S$$

In Equation 8, *I*_limit_ is the detection limit in the photocurrent. In the case where the number of measurements of the dark current significantly exceeds that of the photocurrent (*e.g*., 500 digitized dark current data points vs. tens of photocurrent data points), then *I*_limit_ will reach 3.29σ_dark_, where σ_dark_ is the standard deviation in the dark current. *A* and *S* are the detector area and sensitivity, respectively.^[^ ^79^ ^]^ The sensitivity should be measured with the same applied field as the dark and photocurrent measurements. It should also be pointed out that just as the device architecture influences the sensitivity due to photoconductive gain, the LoDD is also affected. For example, Cao and co‐workers showed that whereas Pb/MAPbI_3_/Au photodiodes had an LoDD of 2.4 nGy_air_ s^−1^ under 2 V reverse bias, the LoDD increased to 77.1 nGy_air_ s^−1^ under 2 V forward bias (concept illustrated in Figure 4e).^[^ ^79^ ^]^ In the current field, there are not yet any widely‐adopted standards in how sensitivity and LoDD are measured, or consideration for how these values are influenced by photoconductive gain.

#### 2.2.3 High Spatial Resolution and Imaging Requirements

**Figure 6:**
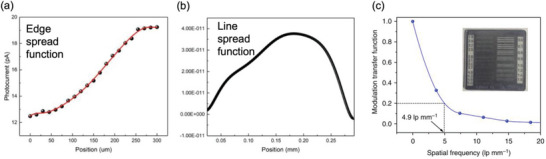
Determining the spatial resolution of an X‐ray imager. The a) edge spread function (ESF) is first determined by measuring the photocurrent from the imager across a precisely‐defined edge attenuating the X‐ray source. The first derivative of the ESF is calculated to give b) the line spread function, and a Fourier transform is taken to obtain c) the modulation transfer function *vs*. the spatial frequency. Reproduced under the terms of the CC‐BY license.^[^ ^69^ ^]^ Copyright 2019, The Authors. Published by Springer Nature.

Beyond effective signal detection (high sensitivity and low detection limit), a high spatial resolution is also required for imaging applications. Spatial resolution describes the smallest distinguishable separation between two points, and is often measured from a line‐pair resolution test, in which the largest number of line pairs (lp per unit distance) is determined, as measured in lp mm^−1^.^[^ ^33^, ^69^, ^81^ ^]^ The ability to achieve high imaging contrast at high resolution is measured through the modulation transfer function (MTF). The MTF is the ratio between the contrast in the image plane vs object plane, and is calculated from MTF = (*I*_max_ – *I*_min_)/(*I*_max_ + *I*_min_), where *I*_max_ is the maximum intensity and *I*_min_ the minimum intensity, and can be measured by imaging a series of black and white lines with different values of line pairs per mm.^[^ ^105^ ^]^ Alternatively, the plot of MTF vs the spatial frequency can be obtained from a Fourier transform of a Point Spread Function (by imaging a single point) or (more commonly) a Line Spread Function (LSF) by imaging a narrow slit.^[^ ^106^, ^107^ ^]^ However, measuring a point or slit requires high‐precision fabrication, fine alignment with the radiation beam, and corrections for the finite value of the slit. A more robust method for measuring the MTF is by measuring an opaque object with a straight edge and measuring the photocurrent in the detector as a function of position around the edge (i.e., the Edge Spread Function, or ESF), and differentiating to obtain the LSF, before taking the Fourier transform to calculate the MTF.^[^ ^106^ ^]^ An example of an ESF for the Au/Cs_2_AgBiBr_6_/Au photoconductor is shown in **Figure** **6**a, and the calculated LSF and MTF as a function of spatial frequency are shown in Figure 6b,c. The spatial resolution at an MTF of 20% for the Cs_2_AgBiBr_6_ photoconductor device is 4.9 lp mm^−1^.^[^ ^69^ ^]^ For a state‐of‐the‐art α‐Se detector, the spatial resolution at an MTF of 20% is 7.1 lp mm^−1^.^[^ ^81^ ^]^ For radiology, a resolution of 5.7 lp mm^−1^ is needed, whereas mammography has more stringent requirements, with a minimum required resolution of 10 lp mm^−1^.^[^ ^53^ ^]^

An important factor that influences spatial resolution is the transit time of charge‐carriers to the electrodes vs. neighboring pixels. Signal crosstalk will reduce the spatial resolution, and this can be prevented if there is little ion migration in the material, such that a large electric field can be applied to reduce the transit time between electrodes, whilst avoiding dark current drift.^[^ ^69^ ^]^

The increase in dark current due to ion migration can also lead to increases in the shot (or white) noise,^[^ ^69^, ^78^ ^]^ which arises due to the random generation of photoelectrons, and the shot noise power is proportional to current.^[^ ^108^ ^]^ Noise is the random fluctuation in the output current, and other types of intrinsic noise common in radiation detectors are scintillation (1/*f*, or low‐frequency) and thermal noise.^[^ ^69^, ^78^, ^99^ ^]^ Whereas shot noise is independent of the frequency, the scintillation noise power is inversely proportional to the frequency, and is typically dominant at low frequencies below 1 kHz. Scintillation noise is found in nearly all detectors, and can be caused by surface traps, or the uneven distribution of impurities.^[^ ^69^, ^108^ ^]^ Thermal noise increases proportionally with temperature, and arises due to the random thermal motion of charge‐carriers.^[^ ^108^ ^]^

The overall signal‐to‐noise in the detector can be quantified by the detective quantum efficiency (DQE) as a function of spatial frequency. DQE is taken as the square of the ratio of SNR_out_ to SNR_in_. SNR_out_ is the SNR discussed earlier in Section 2.2.2 (i.e., relating to the photocurrent and dark current of the detector), whereas SNR_in_ refers to the variation in the radiation source for the detectors.^[^ ^109^ ^]^ Thus, DQE can be considered as how much the detector can keep the signal‐to‐noise ratio in the output photocurrent as high as the input radiation source. Typically, DQE would decrease as the spatial frequency increases.^[^ ^39^ ^]^ The DQE for α‐Se is typically 0.35‐0.4, well below the DQE for indirect detectors due to the low stopping power of Se.^[^ ^57^ ^]^

In addition, a high‐performance X‐ray imager would have a high dynamic range, which describes the range of X‐ray intensities that can be imaged. Detectors with high dynamic range will allow a high degree of contrast to be obtained over a wide range of exposure levels.^[^ ^33^, ^57^ ^]^ The dynamic range is quantified as the ratio of the maximum X‐ray fluence that can be accommodated to the fluence associated with the detector and X‐ray quantum noise. Thus, the lower end of the dynamic range is affected by noise, and therefore dark current.^[^ ^39^ ^]^ Tang and co‐workers calculated that to achieve the largest possible dynamic range, a dark current of 1 pA mm^−2^ or smaller is required.^[^ ^69^ ^]^ Alternatively, the back‐end electronics can be used to enhance the image contrast and therefore increase the dynamic range.^[^ ^57^, ^69^ ^]^

#### 2.2.4 High Time and Energy Resolution, and Durability

Static and dynamic digital radiography requires 1 or 30 frames s^−1^, respectively (Table 1). Operating at these low frame rates requires low modulation frequencies, where 1/*f* noise dominates, and which needs to be minimized.^[^ ^69^ ^]^ On the other hand, the highest frequency that detectors can operate at depends on the cutoff frequency, which is the frequency at which the output photocurrent decreases by 3 dB (i.e., approximately halved) compared to the low‐frequency photocurrent. This depends on the mobility‐lifetime product and radiation absorption profile in the detector, among other factors.^[^ ^99^ ^]^ Finally, it is important that radiation detectors have a short response time, in order to image at a high frame rate and minimize the duration of a pulse of radiation, thus minimizing any possible damage to the subject.^[^ ^110^ ^]^

Energy resolution is particularly important for spectroscopy applications, and is defined as the ratio of the full width at half maximum (FWHM) of the photopeak compared to the photon energy associated with the peak.^[^ ^30^ ^]^ High energy resolution is necessary in γ‐ray spectroscopy to distinguish between different radionucleotides (see Table 1). Achieving high energy resolution requires a low leakage current in order to minimize the background noise.^[^ ^45^ ^]^ Energy resolution is also influenced by device architecture factors, such as the capacitance of the device, the electronics used, as well as intrinsic statistical factors, namely due to the incident radiation not being entirely used to form ion pairs.^[^ ^111^ ^]^

Beyond these requirements, it is also important that the materials exhibit high radiation hardness, and retain their performance over a wide range of photon energies, as well as at high photon fluxes.^[^ ^30^ ^]^ In addition, it is important that the detector is stable under operation. This is described by the polarization effect, which refers to any long‐term changes to the performance of the system with the application of an external field. Changes include drift in the dark current or reductions in the internal electric field, which can both arise from ion migration,^[^ ^33^ ^]^ and will cause reductions in CCE and sensitivity. Finally, the detectors need to be durable and operable over the lifetime of the product (e.g., 4–7 years currently for a commercial CT tube).^[^ ^112^ ^]^

## 3 Properties and Potential of Halide Perovskites and their Derivatives as Radiation Detectors

The previous section specified the properties of the material required to optimize the performance metrics, and some of the performance requirements of radiation detection applications, as well as the challenges of incumbent technologies. This section discusses how the properties of emerging materials compare with requirements, emphasizing both advantages and current challenges. In this section, we focus on the properties of single crystals, and expand the discussion to thick films and wafers later in Section 4 and 5.

### 3.1 Lead‐Halide Perovskites

Lead‐halide perovskites have a 3D crystal structure (typically cubic, orthorhombic, or tetragonal), and an ABX_3_ stoichiometry, where A is a monovalent cation (e.g., Cs^+^ or CH_3_NH_3_^+^), B is Pb^2+^ and X a monovalent anion (usually I^−^, Br^−^ or Cl^−^, but pseudo‐halide anions have also been used).^[^ ^8^, ^9^, ^114^, ^115^ ^]^ Among the many advantages of the lead‐halide perovskite family of materials is their high versatility, in that their dimensionality, bandgap, and optoelectronic properties can be tuned over a wide range through the composition.^[^ ^114^ ^]^ For example, tuning the halide anion adjusts the bandgap across the visible wavelength range, while increasing the size of the A‐site cation in the cuboctahedral vacancies adjusts the degree of octahedral tilting in a 3D structure, or can lower the structural and electronic dimensionality to 2D.^[^ ^114^, ^116^, ^117^, ^118^ ^]^ Furthermore, these materials can be grown as single crystals,^[^ ^99^, ^119^ ^]^ bulk powders,^[^ ^69^ ^]^ and thick or thin films (both single crystal and polycrystalline),^[^ ^28^, ^120^, ^121^, ^122^ ^]^ at low temperatures (often at up to 100 ˚C) using a wide variety of solution‐ and vapor‐based methods. It is estimated that single crystals of LHPs would cost in the range of US$0.5–1.0 cm^−3^,^[^ ^123^ ^]^ which is substantially lower than the cost of CZT single crystal detectors, which is approximately US$2000–3000 cm^−3^.^[^ ^30^, ^124^ ^]^ A summary of these growth methods is given in other Reviews.^[^ ^52^, ^125^, ^126^ ^]^ In this section, we focus on the key properties of the 3D lead‐halide perovskites that make them well suited to radiation detection, spectroscopy and imaging, and also discuss some of the key challenges. The discussion is extended towards lower dimensional perovskites in Section 4.1.

#### 3.1.1 High Stopping Power

**Figure 7:**
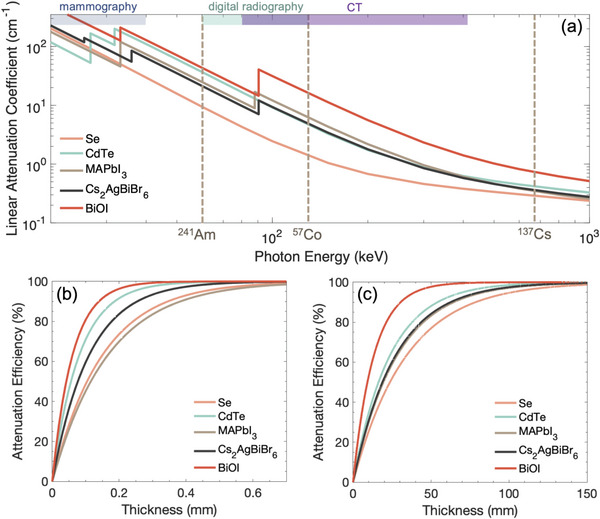
a) Linear attenuation coefficient of established (α‐Se, CdTe) and a selection of emerging radiation detector materials for ionizing radiation. The energy regions used for mammography, digital radiography and computed tomography (CT) scans are highlighted (see Table 1), along with the energies associated with common γ‐ray sources. The attenuation coefficients were calculated using Ref. [113], along with the densities of the materials.^[^ ^54^ ^]^ Attenuation efficiency of materials to b) 30 keV and c) 100 keV radiation calculated using the linear attenuation coefficients of these materials.

The stopping power, as a descriptor of the ability of the material to attenuate ionizing radiation, is typically characterized by the product between the mass attenuation coefficient (*µ*_ac_; see Section 2.2.1) and mass density of the material.^[^ ^30^ ^]^ As stated in the introduction, the mass attenuation coefficient depends on *Z*_av_ *^n^*. The composition of the heavy elements Pb and I in LHPs, as well as modest densities exceeding 3.5 g cm^−3^ (**Table** **3**),^[^ ^30^ ^]^ results in high linear attenuation coefficients (**Figure** **7**a).

**Table 3:** Champion properties and performance of lead‐halide perovskites and perovskite‐inspired materials for X‐ray detection. *Z*_eff_ values calculated from Equation 10, with the exponent *n* taken to be 2.94.^[^ ^127^ ^]^ Note that MA = CH_3_NH_3_^+^, FA = CH(NH_2_)_2_^+^, DMA = (CH_3_)_2_NH_2_^+^, GA = C(NH_2_)_3_^+^. As can be seen from this table, there is no consistency in the X‐ray energy, spectrum or dose rate used for measuring performance. Since each of these parameters influence sensitivity and LoDD, this prevents the direct comparison between different reports. Also, LoDD depends on the applied bias used, as well as the X‐ray energy and spectrum. However, this table serves as a useful comparison of the values reported in the literature for different materials, along with the key properties of these materials. Please note that the lead‐halide perovskite materials focused on here are 3D perovskites with a vertical or horizontal photoconductor or photodiode device architecture. Low‐dimensional perovskites are covered in Section 4, and a comparison with phototransistors was given earlier in Table 2

Material	*Z*_eff_	Density [g cm^−3^]	*µ*_ac_ at 100 keV [cm^−1^]	*µτ* [cm^2^ V^−1^]	Resistivity [Ω cm]	Sensitivity [µC Gy_air_^−1^ cm^−2^]	LoDD [nGy_air_ s^−1^]	X‐ray peak energy	Reference
Commercial‐standard materials									
α‐Se	34	4.28	2.5	10^−7^	10^14^	20	5500	20‐40 keV	[61]
Cd_1‐_ *_x_* Zn*_x_* Te (*x* < 0.2)	48–52	5.81	9.2	10^−5^–10^−2^	10^8^–10^11^	15,200	<14.3	35‐75 keV
HgI_2_	80, 53	6.36	21	10^−9^–10^−5^	10^10^–10^14^	1600	10 000	70 kVp	[130, 131, 132]
Lead‐halide perovskites									
MAPbI_3_	65.0	3.95‐4.15	11.8	10^−2^	n.r.	7 × 10^5^	1.5	22 keV (peak)	[102, 123, 133]
MAPbBr_3_	63.6	3.45	12	10^−2^	n.r.	2.1 × 10^4^	36	8 keV	[134, 135]
MAPbBr_2.94_Cl_0.06_	63.7	4.0	n.r.	1.8 × 10^−2^	3.6 × 10^9^	8.4 × 10^4^	7.6	8 keV
(DMA)MAPbI_3_	–	–	–	7.2 × 10^−3^	3.0 × 10^7^	1.2 × 10^4^	16.9	40 kVp	[136]
(GA)MAPbI_3_	–	–	–	1.3 × 10^−2^	2.1 × 10^8^	2.3 × 10^4^	16.9	40 kVp	[136]
α‐FAPbI_3_	64.6	4.10	–	–	–	1.5 × 10^5^	≈267	40 kVp	[82, 137]
δ‐FAPbI_3_	64.6	4.10	≈10	1.1 × 10^−4^	5.0 × 10^9^	591	81	50 keV
FA_0.9_Cs_0.1_Pb(I_0.9_Br_0.1_)_3_	64.5	–	–	8.5 × 10^−3^	–	1.0 × 10^3^	≈90 (≈40 extrap.)	27 kVp	[119]
FA_0.85_GA_0.05_Cs_0.1_Pb(I_0.9_Br_0.1_)_3_	–	–	–	1.1 × 10^−2^	–	1.0 × 10^4^	≈20 (≈8.5 extrap.)	27 kVp	[119]
FA_0.85_GA_0.05_Cs_0.1_Pb(I_0.9_Br_0.1_)_3_ doped with Sr ^a)^	–	–	≈15	1.3 × 10^−2^	–	2.7 × 10^4^	≈20 (7.09 extrap.)	27 kVp	[119]
FA_0.85_MA_0.1_Cs_0.05_PbI_2.55_Br_0.45_	64.5	–	–	1.8 × 10^−3^	4.6 × 10^7^	3.5 × 10^6^	<42	40 kVp	[82]
δ‐CsPbI_3_	64.4	5.38	23	3.6 × 10^−3^	7.9 × 10^9^	2370	219	50 kVp
CsPbBr_2.9_I_0.1_	65.8	4.85	23	5.06 × 10^−3^	8 × 10^9^	62 748	117	120 keV	[95]
CsPbBr_3_ ^b)^	63.1	4.55	23	1.32 × 10^−2^	10^4^	55 684	215	30 keV	[40, 45, 95, 96]
Bismuth‐based perovskite‐inspired materials									
Cs_2_AgBiBr_6_	56.7	4.65	10	5.95 × 10^−3^	10^10^	1974	45.7	30 keV
Cs_2_AgBiBr_6_ (film)	56.7	4.65	10	–	–	1.8 × 10^4^	145.2	83 keV	[140]
MA_3_Bi_2_I_9_	64.6	3.8‐3.98	15	2.8 × 10^−3^	10^11^	10 620	6 (0.62 extrap.)	n.r.
(NH_4_)_3_Bi_2_I_9_	63.2	4.3	n.r.	ǁ: 1.1 × 10^−2^ ⊥: 9.1 × 10^−3^	n.r.	ǁ: 8200 ⊥: 803	ǁ: 210 ⊥: 55	n.r.
Rb_3_Bi_2_I_9_	61.4	4.75	15	2.51 × 10^−3^	10^9^	159.7	8.32	50 kVp	[51, 143]
Cs_3_Bi_2_I_9_	62.3	5.02	‐	1.35 × 10^−3^	10^12^	4382	7.93	35 kVp
Cs_3_Bi_2_Br_9_	60.1	4.73	12	8.32 × 10^−4^	10^12^	1705	1.2 (0.58 extrap.)	120 keV
BiI_3_	66.8	5.78	25	10^−5^–10^−3^	10^9^–10^13^	10^4^	34	70 keV
BiOI	73.2	7.97	40	ǁ: (6±2) × 10^−2^ ⊥: (1.1±1.4) × 10^−3^	ǁ: 10^12^ ⊥: 10^9^	1100 (⊥)	22 (⊥) (1.1 extrap.)	7.9 keV	[54]
AgBi_2_I_7_	64.9	10.29	40	(1.2–3.4) × 10^−3^	1.3 × 10^8^	282.5	110 (72 extrap.)	43 keV
Other notable materials									
TMCM‐CdCl_3_ ^c)^	–	2.19	n.r.	10^−4^	10^7^	129	1060	29 keV	[154]
DABCO‐NH_4_Cl_3_ ^d)^	–	1.40	0.3	10^−3^	10^9^	165	‐	29 keV
DABCO‐NH_4_Br_3_ ^d)^	–	2.03	0.8–2	10^−4^–10^−3^	10^8^–10^9^	173–176	4960	29 keV	[155, 156]
DABCO‐NH_4_I_3_ ^d)^	–	2.58	6	10^−3^	10^9^	567	‐	29 keV
Cs_2_TeI_6_	53.4	3.2	≈10	–	10^11^	226.8	<115	50 kVp	[157, 158]

^a)^ For single crystals of FA_0.85_GA_0.05_Cs_0.1_Pb(I_0.9_Br_0.1_)_3_ doped with Sr, the sensitivity reported is at a bias field of 1 V cm^−1^ and X‐ray tube set at 27 kVp, which is also the condition under which the limit of detection was measured. Increasing the bias field to 400 V cm^−1^ and X‐ray tube voltage to 60 kVp increases the sensitivity by two orders of magnitude to 2.7 × 10^6^ µC Gy_air_^−1^ cm^−2^.^[^ ^119^ ^]^

^b)^ This sample was prepared as a thick (hundreds of microns) film by hot pressing, and the device structure was glass/FTO/CsPbBr_3_/Au^[^ ^96^ ^]^

^c)^ TMCM = (CH_3_)NCH_2_Cl, or trimethylchloromethyl; TMCM‐CdCl_3_ achieves ≈95% attenuation efficiency for 40 keV X‐rays with 2 mm thick crystals, which is lower than CdTe but significantly larger than Si^[^ ^154^ ^]^

^d)^ DABCO is *N*,*N*’‐diazabicyclo[2.2.2]octonium^[^ ^156^ ^]^

The effective average atomic number of a compound composed of different elements can be estimated by Equation 9, developed by Oto et al.

(9) $$Z_{\mathrm{eff}}=\frac{\sigma_{a}}{\sigma_{e}}\;=\frac{\mu_{\rho }\frac{\sum n_{i}M_{i}}{\sum n_{i}}}{\sum \frac{M_{i}}{Z_{i}}f_{i}\mu_{i}}\;$$

In Equation 9, σ_a_ and σ_e_ are the total atomic cross sections and total electronic cross‐sections, respectively. For the i^th^ element, *n*_i_ is the number of this atom per formula unit, *M*_i_ is its molar mass, *f*_i_ is its fractional abundance, and *Z*_i_ its atomic number.^[^ ^159^ ^]^ However, this requires careful measurement of the atomic and electron cross‐sections. A simpler way to calculate *Z*_eff_ is given by Equation 10, which was derived by Ishii and Kobayashi.^[^ ^160^ ^]^

(10) $$Z_{\mathrm{eff}}={\left(\frac{\sum_{i}\left[f_{i}M_{i}{\left(Z_{i}\right)}^{n}\right]}{\sum_{i}\left[f_{i}M_{i}\right]}\right)}^{1/n}\;$$

In Equation 10, *f_i_*, *M*_i_, and *Z*_i_ are the stoichiometry, molar mass, and atomic number of element *i* in the compound, respectively. *n* is an exponent, and common values that have been taken are 2.94 and 4.^[^ ^127^ ^]^ This equation only provides a first‐order approximation of *Z*_eff_, and it is assumed that photoelectric absorption dominates, and that the molar masses are proportional to the *Z* values.^[^ ^160^ ^]^ From both equations, it can be seen that whilst it is important to have high‐*Z* elements, achieving a high overall *Z*_eff_ requires their fraction to also be high in the material. For example, Bi has a higher atomic number (83) than Pb (82), and yet MA_3_Bi_2_I_9_ has a slightly lower *Z*_eff_ (64.6) than MAPbI_3_ (65.0) because of the lower overall fraction of Bi in MA_3_Bi_2_I_9_ than Pb in MAPbI_3_.

Overall, LHPs have *Z*_eff_ values substantially exceeding α‐Se (34), and are also larger than in CdTe (50.2—as calculated from Equation 10), as shown in Table 3. These differences in *Z*_eff_ are accentuated in the mass attenuation coefficient, due to the exponential dependence on *Z*_eff_. Thus, although MAPbI_3_ has a lower mass density than CdTe, the linear attenuation coefficient at 100 keV (11.8 cm^−1^) is larger than CdTe (9.2 cm^−1^), and substantially larger than α‐Se (2.5 cm^−1^), as shown in Table 3 and Figure 6. The thickness of material needed to attenuate 99% of the incident radiation at 100 keV would increase from 3.9 mm (MAPbI_3_) to 5.0 mm (CdTe) and 18.4 mm (α‐Se), as calculated using Equation 3. If we look more broadly among semiconductors used for radiation detection, LHPs have significantly stronger attenuation for X‐rays than molecular perovskites, but are on a similar level to HgI_2_, which contains heavy Hg (Table 3). A smaller required thickness to attenuate ionizing radiation means that a high charge collection efficiency can be achieved using a smaller applied bias, and therefore lower dark current. This, among other factors (discussed below) contributes to a lower LoDD, as well as reduced cross‐talk in imaging. As can be seen from Table 3 and Figure 2, the LoDD achieved with LHPs has reached as low as 1.5 nGy_air_ s^−1^ for MAPbI_3_,^[^ ^102^ ^]^ which is several orders of magnitude smaller than the LoDDs reported for α‐Se or molecular perovskite‐based detectors (TMCM‐Cd and DABCO‐NH_4_X_3_, Table 3).

#### 3.1.2 High Radiation Hardness

Radiation hardness is commonly referred to as the ability of materials and devices to maintain, or regain, their performance (e.g., luminescence quantum yield of a material, or photocurrent of a device) after exposure to high dose rates of ionizing radiation, or after extended continuous or repeated exposure.^[^ ^40^, ^161^, ^162^ ^]^ LHPs have demonstrated an outstanding radiation hardness to different types of ionizing radiation, which in most cases have been found to be at least equivalent, or better than commercial CZT detectors.^[^ ^163^ ^]^ For example, Zaffalon et al. recently reported that CsPbBr_3_ perovskite scintillators could maintain their radioluminescence efficiency after exposure to 1 MGy of ^60^Co γ‐rays (≈1.25 MeV mean energy).^[^ ^40^ ^]^ By contrast, commercial CZT detectors suffer from a reduction in performance after exposure to 30 kGy γ‐rays (also ^60^Co).^[^ ^163^, ^164^ ^]^ More broadly, LHP single crystals, thin films, and nanocrystals have been shown to be tolerant to X‐rays, *β*‐particles, electrons, and protons.^[^ ^161^, ^165^, ^166^, ^167^, ^168^, ^169^ ^]^ The high radiation hardness of LHPs is attributed to its high defect tolerance, ability to self heal, as well as high amenability to a wide variety of passivation strategies.

**Figure 8:**
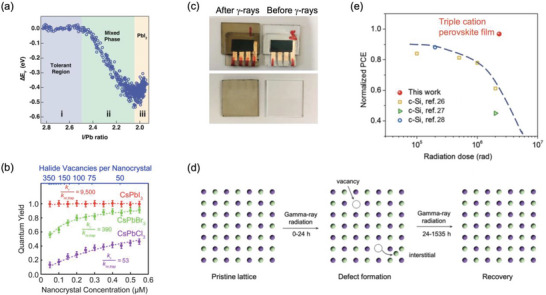
Defect tolerance and self‐healing of lead‐halide perovskites. a) Plot of the shift in valence band to Fermi level offset from the pristine MAPbI_3_ thin film sample (Δ*E*) as a function of the I/Pb ratio. Reproduced with permission.^[^ ^172^ ^]^ Copyright 2016, American Chemical Society. b) Photoluminescence quantum yield of CsPbX_3_ nanocrystals (X = I, Br or Cl), as a function of their concentration. As the concentration is decreased, the surface halide vacancy content increases. Reproduced with permission.^[^ ^173^ ^]^ Copyright 2018, American Chemical Society. c) Photographs of triple cation perovskite photovoltaic devices (top) and indium tin oxide (ITO)‐coated glass substrates (bottom) before and after exposure to 2.3 Mrad γ‐rays after 1535 h. d) Proposed mechanism for self‐healing in the perovskite films during γ‐ray exposure. e) Comparison of the normalized power conversion efficiency (PCE) of triple‐cation perovskite photovoltaics versus c‐Si photovoltaics as a function of the radiation dose. Parts (c–e) reproduced with permission.^[^ ^175^ ^]^ Copyright 2018, Wiley‐VCH GmbH.

Defect tolerance refers to the lifetime and mobility of free charge‐carriers being unaffected by an increase in trap density.^[^ ^15^, ^19^ ^]^ In LHPs, this can occur because dominant defects (with low formation energy) have shallow transition levels, enabling low rates of Shockley‐Read‐Hall recombination to be maintained despite high trap densities.^[^ ^170^ ^]^ Furthermore, the highly polarizable nature of LHPs results in these materials having high dielectric constants.^[^ ^170^ ^]^ Coupled with low effective masses, these factors result in low Sommerfeld enhancement factors for charged defect states, and therefore low capture cross‐sections.^[^ ^71^ ^]^ Several works have provided experimental evidence in support of defect tolerance in LHPs.^[^ ^171^ ^]^ Steirer et al. exposed MAPbI_3_ to continuous X‐ray exposure under vacuum, which led to the continuous removal of MAI. It was shown that the I/Pb ratio could be reduced to 2.5 before there was a shift in the Fermi level, showing there to be a large defect‐tolerant compositional range (**Figure** **8**a).^[^ ^172^ ^]^ Alivisatos and co‐workers similarly showed that CsPbI_3_ nanocrystals can maintain a photoluminescence quantum yield (PLQY) close to unity despite the introduction of 350 halide vacancies per nanocrystal (Figure 8b).^[^ ^173^ ^]^ However, not all traps in perovskites are shallow, and the likelihood of forming deep traps increases as the bandgap becomes wider (see CsPbCl_3_ in Figure 8b).^[^ ^19^, ^173^ ^]^ Deep level transient spectroscopy (DLTS) measurements on polycrystalline perovskite thin films showed there to be deep traps 0.76 eV below the band‐edge, but the capture cross‐section was only 10^−14^ cm^2^. 18%‐efficient LHP photovoltaics were therefore still achievable.^[^ ^174^ ^]^ Thus, exposure to ionizing radiation and introducing point defects in LHPs can still allow large mobility‐lifetime products to be retained.

Many groups have also reported an initial decrease in performance with ionizing radiation exposure, followed by recovery in the dark.^[^ ^176^, ^177^ ^]^ This was attributed to self‐healing, which involves the redistribution of ions in the lattice, facilitated by ion migration, allowing the recovery of the lattice from radiation‐induced point defects (e.g., vacancies).^[^ ^175^, ^177^, ^178^, ^179^ ^]^ This is consistent with the slow timescale of recovery, on the order of seconds to minutes.^[^ ^177^ ^]^ It is also believed that self‐healing can occur simultaneously to defect formation under ionizing radiation exposure. For example, Yang et al. exposed triple cation perovskite solar cells to γ‐rays (and 1‐sun illumination) for 1535 h, observing no change in the appearance of the perovskite, while the ITO‐coated glass substrate had significant discoloration (Figure 8c). The perovskite solar cells exhibited an initial decrease in performance, followed by the efficiency reaching steady‐state.^[^ ^175^ ^]^ The steady‐state conditions were believed to be due to ion migration of species displaced from the lattice due to γ‐ray exposure returning to the crystallographic lattice site, which are thermodynamically favored (Figure 8d). Overall, the perovskite solar cells retained 96.8% of their initial power conversion efficiency after exposure to a total of 2.3 Mrad of γ‐rays, whereas c‐Si solar cells could only retain 61.2% of their initial efficiency after approximately 2 Mrad of γ‐rays exposure (Figure 8e).^[^ ^175^ ^]^ At the same time, completely healing the defects induced through radiation exposure can only occur if the removal of species through volatilization is avoided (e.g., avoiding MAI removal).^[^ ^166^ ^]^

It has also been observed that passivating surface defect states can improve the stability of LHPs.^[^ ^180^, ^181^, ^182^, ^183^ ^]^ Zaffalon *et al.* found that CsPbBr_3_ nanocrystals have a radioluminescence (RL) peak that was red‐shifted compared to the photoluminescence (PL) peak, and this was ascribed to RL originating from emission via shallow traps. Exposure to γ‐rays from a 1.25 MeV ^60^Co source led to a tail in absorbance emerging over time, which is attributed to the agglomeration of NCs. This agglomeration was suppressed by passivating the surface bromide vacancies using fluorine (provided via didodecyl dimethylammonium fluoride). Both the untreated and F‐passivated CsPbBr_3_ maintained the same phase and PL quantum yield after 1 MGy γ‐ray exposure, showing their high radiation hardness, whereas the polypropylene vials used to contain the colloidal solutions became brittle after the same exposure. The CsPbBr_3_:F nanocrystals had the added advantages of avoiding agglomeration and maintaining high colloidal stability. Furthermore, the fluorinated samples had higher PL quantum yields (90±7%, compared to 48±5% for non‐passivated samples), and had the RL matching the PL spectrum, showing RL to originate from band‐edge radiative recombination.^[^ ^40^ ^]^

**Figure 9:**
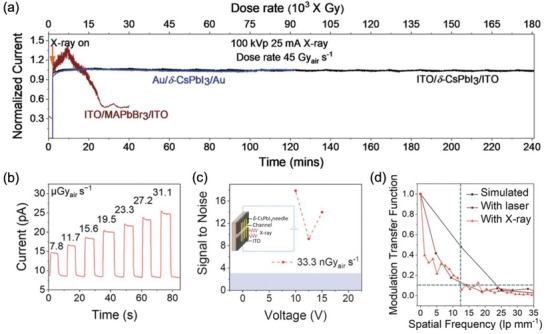
X‐ray detector performance of δ‐CsPbI_3_ microwires. a) Operational stability of MAPbI_3_ and δ‐CsPbI_3_ photoconductors under continuous exposure to X‐rays (45 Gy_air_ s^−1^ dose rate, 100 kVp). b) Current from δ‐CsPbI_3_ microwire detectors with and without exposure to 50 kVp X‐rays of different dose rates (labeled in µGy_air_ s^−1^). c) SNR of the photocurrent under 33.3 nGy_air_ s^−1^ dose rate at different applied biases. Device structure inset. d) Modulation transfer function to measure the spatial resolution of the δ‐CsPbI_3_ detectors. Reproduced with permission.^[^ ^53^ ^]^ Copyright 2022, Wiley‐VCH GmbH.

Finally, the stability of the perovskite itself also has an important influence on its radiation hardness. For example, Kundu et al. showed that MAPbBr_3_ devices (unencapsulated and in ambient air) decomposed after 9 min of exposure to 100 kVp X‐rays to form PbBr_2_ and PbO. By contrast, δ‐CsPbI_3_ is more environmentally and thermally stable, and maintained the same photocurrent when exposed to X‐rays under the same conditions, even after a total dose of 1.44 × 10^6^ Gy_air_ (**Figure** **9**a). This is equivalent to 720 million chest X‐rays.^[^ ^53^ ^]^

#### 3.1.3 High Mobility‐Lifetime Products

An outstanding property that became apparent in the early development of lead‐halide perovskites is its long diffusion lengths, which exceeds 1 µm in polycrystalline thin films, corresponding to a mobility‐lifetime (*µτ*) product of 4 × 10^−7^ cm^2^ V^−1^.^[^ ^184^, ^185^ ^]^ In single crystals, both the mobility and lifetime increase due to reduced scattering from structural defects (e.g., grain boundaries), as well as reduced non‐radiative recombination, leading to longer charge‐carrier lifetimes. The *µτ* products typically reported from bulk 3D LHP single crystals exceed 10^−2^ cm^2^ V^−2^ (Table 3), with ambipolar electron‐hole diffusion lengths exceeding 175 µm.^[^ ^186^ ^]^ Given the high stopping power of LHPs, a sufficiently long drift length (or Schubweg) that is comparable to the required thickness of the crystal can be achieved to obtain a high CCE without requiring a large applied electric field.

As discussed in Section 2.2.1, high charge‐collection efficiencies are needed for high sensitivities. Sensitivities exceeding 10^3^ µC Gy_air_^−1^ cm^−2^ have been widely reported in bulk 3D LHP single crystals detectors for X‐ray illumination (Figure 2, Table 3), outperforming α‐Se detectors, as well as many CZT detectors (see Tables 1 and 3).^[^ ^58^, ^61^, ^66^ ^]^ A high *Z*_eff_ and large *µτ* product also enable the detection of ionizing particles (e.g., γ‐rays) with high energy resolution. CsPbBr_3_ single crystal detectors with 1.4% energy resolution to 662 keV ^137^Cs γ‐rays have been achieved.^[^ ^55^ ^]^ Realizing such a high energy resolution at room temperature to high‐energy ionizing radiation is remarkable, and makes these detectors competitive with the current state‐of‐the‐art for room temperature γ‐ray detectors (CZT, 0.5% energy resolution), as well as high‐purity Ge (0.14% at 77 K).^[^ ^30^, ^52^ ^]^ In addition to high *µτ* products (8 × 10^−3^ cm^2^ V^−1^) and *Z*_eff_ (67.3), the high energy resolution of CsPbBr_3_ also came about because of a unipolar device structure that minimized dark currents.^[^ ^55^ ^]^ LHP single crystals offer the opportunity to achieve more cost‐effective γ‐ray detectors that are lower cost to produce (compared to expensive CZT single crystals) and operate (at room temperature, as opposed to 77 K for high‐purity Ge).

The exceptionally high *µτ* products achieved in 3D LHP single crystals come about due to the low exciton binding energy (≤25 meV, enabling free charge‐carriers at room temperature, as opposed to excitons),^[^ ^114^, ^187^ ^]^ low effective masses of charge‐carriers (<0.3*m*_0_, where *m*_0_ is the rest mass of electrons),^[^ ^188^ ^]^ defect‐tolerant nature of the materials, and delocalized nature of the charge‐carriers.^[^ ^189^ ^]^ The last point is particularly important. In bismuth‐halide semiconductors, carrier localization has been widely found, and this severely limits charge‐carrier mobilities (see Section 3.2.5).^[^ ^78^, ^188^, ^189^ ^]^ By contrast, in LHP single crystals, mobilities exceeding 100 cm^2^ V^−1^ s^−1^ have been widely reported.^[^ ^12^, ^186^ ^]^ At the same time, these mobilities are below the values expected based on the effective masses, and this is due to intermediate Fröhlich coupling between charge‐carriers and longitudinal optical (LO) phonons. Nevertheless, Fröhlich coupling is not strong, and the deformation potential is low (<7 eV for CsPbBr_3_), implying insufficiently strong coupling to acoustic phonons to form small polarons or self‐trapped excitons.^[^ ^188^ ^]^ The high mobilities achievable in single crystals, coupled with the long charge‐carrier lifetimes (in the hundreds of microsecond range),^[^ ^55^ ^]^ enable large *µτ* products.

#### 3.1.4 Tunable, Low Dark Currents

As discussed in Section 2.2.3, a dark current density lower than 1 pA mm^−2^ is needed to achieve the largest possible dynamic range in radiation detectors,^[^ ^69^ ^]^ unless back‐end electronics are used to enhance image contrast. Indeed α‐Se flat panel imagers are capable of achieving dark current densities of 1 pA mm^−2^ or lower.^[^ ^44^, ^190^ ^]^ Achieving a dark current density this low is challenging in LHPs, but optimized single crystals of bulk 3D LHPs are now capable of reaching down to 10 pA mm^−2^.^[^ ^52^ ^]^

##### Growth of Crystals with Low Defect Density

Key to achieving low dark current densities is growing crystals with a low density of defect states that would donate charge‐carriers to the lattice, and thus maintain a low carrier concentration. Resistivities are typically on the order of 10^9^ Ω cm in LHP single crystals, due to low trap densities, typically in the range of 10^9^–10^11^ cm^−3^.^[^ ^7^, ^186^, ^191^ ^]^ Achieving high resistivities requires careful growth to avoid the formation of structural defects, as well as minimizing the concentration of impurities. This is especially important for γ‐ray spectroscopy, since the photon flux density of γ‐ray sources is usually low, and a small number of charge‐carriers are generated from each absorbed γ‐ray photon, meaning that the signal can easily be lost through trapping. For example, Kanatzidis and co‐workers found that their original solution‐grown CsPbI_3_ single crystals were unable to resolve γ‐ray spectra.^[^ ^29^ ^]^ By purifying their PbBr_2_ precursor (to 99.999%), a two orders of magnitude improvement in resistivity was achieved, reaching 10^11^ Ω cm, giving rise to γ‐ray detectors with 3.4% energy resolution (662 keV ^137^Cs).^[^ ^52^ ^]^ Very recently, Huang and co‐workers found a route to achieve a similar energy resolution of 2.9% using low‐purity (98%) precursors by alloying Cs with FA. Alloying eliminated the phase transition from the growth temperature (60 °C) to room temperature, thus reducing the defect density from 10^12^ cm^−3^ to 5.6 × 10^10^ cm^−3^, and increasing resistivities from 10^6^–10^10^ Ω cm.^[^ ^124^ ^]^

Compositional tuning has also been employed to achieve low defect density in LHP single crystals. For example, Jiang et al. recently alloyed guanidinium (GA) and Sr into Cs_0.1_FA_0.9_Pb(I_0.9_Br_0.1_)_3_ perovskites to control the density of I and Pb vacancies. GA addition decreased the density of I vacancies by strongly bonding to I. However, the large size of GA introduced strain to the lattice, leading to Pb vacancy formation. To suppress these, doping the perovskite with a small quantity of Sr increased the formation energy of Pb vacancies, decreasing their concentration. Through this synergistic compositional tuning and strain engineering, the dark current was reduced to 10 pA mm^−2^ at ‐1 V applied bias, along with low noise (4 × 10^−14^ A Hz^−0.5^), thus leading to low limits of detection. The lowest dose rate directly measured by the authors was at approximately 20 nGy_air_ s^−1^. When linearly extrapolated down to an SNR of 3, the LoDD would be 7.09 nGy_air_ s^−1^ (Table 3).^[^ ^119^ ^]^ Liu et al. also found that using multiple species on the A‐site (i.e., FA_0.85_MA_0.1_Cs_0.05_PbI_2.55_Br_0.45_ triple cation perovskites) relieved lattice stress and reduced ion migration, giving rise to sensitivities reaching as high as (3.5±0.2) × 10^6^ µC Gy_air_^−1^ cm^−2^, along with LoDDs <42 nGy_air_ s^−1^ (Table 3, Figure 2).^[^ ^82^ ^]^ This sensitivity, to our knowledge, is the highest reported for lead‐halide perovskites thus far (Figure 2), but there is also significant photoconductive gain. Liu et al. calculated that the theoretical maximum sensitivity for FA_0.85_MA_0.1_Cs_0.05_PbI_2.55_Br_0.45_ triple cation perovskites is 1.51 × 10^3^ µC Gy_air_^−1^ cm^−2^.^[^ ^82^ ^]^

Beyond low LoDDs, low dark currents have been shown to be important to achieve high imaging resolution. For example, δ‐CsPbI_3_ single crystals were found to have low dark current densities of 12 pA mm^−2^ at 6000 V cm^−1^ applied field (Figure 9b), which was attributed in part to its wide indirect bandgap of 2.67 eV. As a result, a low LoDD of below 33.3 nGy_air_ s^−1^ was reported (note SNR at this dose rate was 17.8, and the limit of detection at an SNR of 3 would be lower; Figure 9c), along with a high imaging resolution of 12.4 lp mm^−1^ at 10% modulation transfer function (Figure 9d). This exceeds the threshold require for radiography and mammography (see Section 2.2.3), but note that most other groups report the imaging resolution at 20% MTF, which in this case would be <10 lp mm^−1^ (Figure 9d).^[^ ^53^ ^]^

##### Self‐Compensation

**Figure 10:**
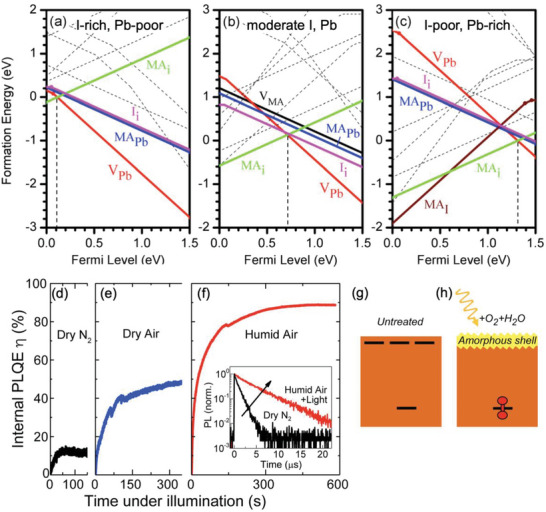
Defects and passivation in lead‐halide perovskites. Defect diagrams for MAPbI_3_ under a) I‐rich, Pb‐poor, b) moderate and c) I‐poor, Pb‐rich conditions. Each line represents a different point defect, and the slope represents the charge of the defect as a function of the Fermi energy relative to the valence band maximum. Positive‐sloped defects are donors, and negative‐sloped defects acceptors. The Fermi energy of the material is pinned by the cross‐over between the lowest formation energy donor and acceptor defects. MAPbI_3_ is therefore *p*‐type in (a), near‐intrinsic in (b), and *n*‐type in (c). Parts (a)–(c) reproduced with permission.^[^ ^162^ ^]^ Copyright 2014, American Institute of Physics. Change in internal photoluminescence quantum efficiency (PLQY) of MAPbI_3_ thin films under illumination in d) dry N_2_, e) dry air, and f) humid air (PL decays inset). g) Illustration of the untreated traps in as‐grown MAPbI_3_, and h) proposed passivation from an amorphous shell formed after illumination in humid air. Parts (d)–(h) reproduced under the terms of the CC‐BY license.^[^ ^193^ ^]^ Copyright 2017, The Authors. Published by the Authors.

Unlike traditional semiconductors, such as crystalline silicon, LHPs are very difficult to dope. This is because LHPs have low formation energy point defects (*e.g*., iodine vacancies), and the Fermi level is often pinned by the overlap between the lowest formation energy donor and acceptor defects (**Figure** **10**a‐c). This is known as self‐compensation. Depending on the chemical potentials during growth, the Fermi level may vary (as shown in Figure 10a‐c), but it is often reported that LHPs are close to intrinsic (or weakly *n*‐ or *p*‐type). The low carrier concentration in LHPs is reflected by the strong depletion of the perovskite when deposited onto *n*‐ or *p*‐type substrates. The low carrier concentration obtained in most LHP materials through self‐compensation is important to achieve low dark currents in this material, in particular to maintain dark currents at a low value when the applied field is increased to increase the drift length.^[^ ^100^, ^192^ ^]^

##### Self‐Doping

At the same time, the exact location of the cross‐over point between the lowest formation energy donor and acceptor intrinsic defects varies depending on the growth conditions. For example, in shifting the chemical potential from moderate to I‐rich and Pb‐poor, the Fermi level shifts from near mid‐gap to close to the valence band maximum, leading to *p*‐type character in the material (Figure 10a,b).^[^ ^192^ ^]^ This is known as self‐doping. Whilst self‐doping in some perovskites (*e.g*., MAPbI_3_) is ambipolar (can be tuned to *p*‐type or *n*‐type, depending on the chemical potentials during growth), MAPbBr_3_ has been found to have unipolar self‐doping towards *p*‐type.^[^ ^194^ ^]^ The *p*‐type self‐doping in MAPbBr_3_ limits how high the resistivities could reach in single crystals (only 2.0 × 10^8^ Ω cm).^[^ ^195^ ^]^ Wei et al. found that the resistivity could be increased through compensating doping with 2% Cl (since MAPbCl_3_ is weakly *n*‐type), raising the resistivity to 3.6 × 10^9^ Ω cm by reducing the carrier concentration.^[^ ^100^ ^]^ This lowered the dark current, whilst simultaneously increasing both the mobility (by a factor of three to 560 cm^2^ V^−1^ s^−1^) and lifetime (by a factor of 10 to approximately 5 µs). Thus, the *µτ* product reached a high value of 1.8 × 10^−2^ cm^2^ V^−1^, and an energy resolution of 6.5% was achieved for the resolution of 662 keV ^137^Cs γ‐rays.^[^ ^100^ ^]^

##### Ideal Bandgap

Another factor contributing to the low dark current densities is the wide bandgap of LHPs, which are in the range of 1.55 eV (MAPbI_3_) to 3.2 eV (MAPbCl_3_).^[^ ^196^ ^]^ Having a wide bandgap results in a low concentration of thermally‐generated charge‐carriers, thus reducing dark current. At the same time, increasing the bandgap too far results in an electron‐hole ionization energy that is too large, which reduces the number of electron‐hole pairs created through photoelectric absorption.^[^ ^36^ ^]^ This reduces the SNR, which not only could lead to higher LoDDs, but could also result in a longer acquisition time being required to measure the spectrum of a radiation source. The ideal range of bandgaps for radiation detectors is typically taken to be 1.4–2.5 eV,^[^ ^71^ ^]^ which encompasses iodide and bromide LHPs.

#### 3.1.5 Traps Amenable to Passivation and Mitigation

Despite the defect tolerance of LHPs, passivation is essential to reduce the leakage currents in single crystal devices and achieve the best performance, in terms of sensitivity, LoDD, and energy resolution. This is because even though the effect of bulk defects may be minimized (either by a low concentration, or because dominant defects are shallow) dangling bonds still form at surfaces, and can lead to high surface recombination.^[^ ^100^ ^]^ Thus, passivation of these defects at the surface, and any defects formed in the bulk, will be critical. An important advantage of LHPs is that they are highly amenable to a wide variety of passivation strategies, such as through the use of ligands, additives, or dopants.^[^ ^173^, ^180^, ^197^, ^198^ ^]^ For example, the defect tolerance of CsPbBr_3_ is lower than that of CsPbI_3_, in that bromide vacancies form deeper traps than iodide vacancies. The depth of the dominant defect in CsPbBr_3_ was measured to be 260 meV.^[^ ^40^ ^]^ The PLQY of CsPbBr_3_ is therefore lower than CsPbI_3_, and is reduced as the bromide vacancy concentration increases.^[^ ^173^ ^]^ Zaffalon et al. showed that an effective approach to passivating these shallow vacancies was through post‐synthesis fluorination (with a F/Br ratio of 0.081), leading to the PLQY increasing from 48±5% (CsPbBr_3_) to 90±7% (CsPbBr_3_:F).^[^ ^40^ ^]^ Surface defects in LHPs could also be eliminated through passivation from O_2_ and H_2_O physisorption, or via the formation of superoxide species, leading to reductions in surface recombination velocity from ≈1000 cm^2^ s^−1^ (unpassivated LHP single crystals)^[^ ^199^ ^]^ to values as low as 0.4‐4 cm^2^ s^−1^ (Figure 10d‐h).^[^ ^193^, ^200^ ^]^ Beyond these passivation strategies, the effects of traps can be mitigated through heteroepitaxial passivation, reducing the dimensionality of the perovskite, as well as through judicious choice of the charge extraction layers. These points are covered later in Section 4.

### 3.2 Perovskite‐Inspired Materials

An important limitation of lead‐halide perovskites is its high quantities of neurotoxic, bioaccumulative lead, which is readily available, due to the high solubility of the perovskites in polar solvents (e.g., water).^[^ ^8^, ^14^ ^]^ Furthermore, the concentration of lead exceeds the strict limits set in many jurisdictions (e.g., 0.1 wt.% for the EU Restriction of Hazardous Substances Directive for consumer electronics),^[^ ^201^ ^]^ and is compounded by the fact that larger masses of material are needed for radiation detectors than in thin film optoelectronic devices. A counter‐argument is that radiation detectors are usually well sealed away from the patient or user. On the other hand, the high quantities of lead may prevent halide perovskites from being used for wearable radiation detector monitors.^[^ ^202^ ^]^

Perovskite‐inspired materials (PIMs) describe the classes of compounds investigated as lower toxicity alternatives to lead‐halide perovskites.^[^ ^114^ ^]^ Broadly speaking, there have been three approaches in the community towards discovering PIMs. The first is to identify chemical analogs by substituting out Pb^2+^ for other divalent main group cations (namely Sn^2+^ and Ge^2+^). The second is to find structural analogs, such as double perovskites (e.g., Cs_2_AgBiBr_6_) and vacancy‐ordered double and triple perovskite (e.g., Cs_2_SnBr_6_ or Cs_3_Bi_2_I_9_, respectively). The third approach is to find electronic analogues*, i.e*., materials that maintain anti‐bonding orbitals at the band‐edges, with a significant contribution from the pnictogen valence *s* electrons, as found in LHPs. This is because the particular electronic structure of LHPs was believed to be conducive towards achieving defect tolerance,^[^ ^170^ ^]^ and the PIMs investigated have gone beyond the perovskite system towards materials including bismuth oxyiodide (BiOI) and BiSI.^[^ ^54^, ^203^ ^]^

Whilst defect tolerance has been found in some PIMs (e.g., BiOI, both computationally and experimentally),^[^ ^204^, ^205^ ^]^ the performance of PIMs in photovoltaics or light‐emitting diodes has thus far been inferior to LHP devices. Radiation detectors, however, have been an exception, with bismuth‐based PIMs demonstrating lower LoDDs than many LHP devices to values as low as 1.2 nGy_air_ s^−1^ for the vacancy‐ordered triple perovskite Cs_3_Bi_2_Br_9_ (Figure 2).^[^ ^146^ ^]^ This section discusses the properties of bismuth‐based PIMs that make them exceptionally promising radiation detectors, as well as current limitations and recent work showing potential pathways to overcome these limitations.

#### 3.2.1 High Stopping Power and Radiation Hardness

##### Stopping Power

**Figure 11:**
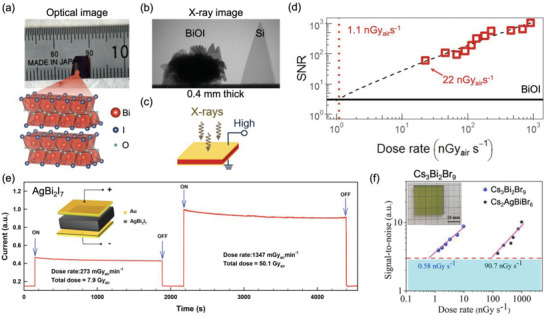
High stopping power, radiation hardness and low dark currents in selected Bi‐based perovskite‐inspired materials. BiOI: a) optical image of BiOI single crystals, along with an illustration of the layered crystal structure; b) image comparing the transmittance of X‐rays through 0.4 mm thick silicon *vs*. a 0.4 mm thick stack of BiOI single crystals; c) illustration of a BiOI photoconductor with transport in the out‐of‐plane direction (i.e., perpendicular device), and d) its signal‐to‐noise ratio (SNR) as a function of dose rate. Parts (a–d) reproduced under the terms of the CC‐BY license.^[^ ^54^ ^]^ Copyright 2023, The Authors. Published by Springer Nature. e) Operational stability of AgBi_2_I_7_ photoconductors, determined by measuring the photocurrent after continuous exposure to 7.9 Gy_air_ and 60.1 Gy_air_ X‐rays. Device structure inset. Reproduced with permission.^[^ ^152^ ^]^ Copyright 2020, American Chemical Society. f) SNR *vs*. dose rate for Cs_3_Bi_2_Br_9_ and Cs_2_AgBiBr_6_ photoconductors, comparing their limits of detection. Reproduced with permission.^[^ ^146^ ^]^ Copyright 2022, American Chemical Society.

As mentioned in Section 3.1.1 and in the introduction, Bi is the heaviest element that is stable against radioactive decay. But Bi needs to be combined with other heavy elements in order to have a high *Z*_eff_. For example, MA_3_Bi_2_I_9_ has a slightly lower *Z*_eff_ (63.6) than MAPbI_3_ (65.0) due to the higher fraction of the organic component, but is still higher than the *Z*_eff_ of CdTe (50.2) and α‐Se (34). This *Z*_eff_ increases to 66.7 for BiI_3_, and 73.2 for BiOI (Table 3). Coupled with its high mass density of 7.97 g cm^−3^, BiOI has high linear attenuation coefficients (Figure 7a). For example, we showed that only 2% of incident X‐rays (40 kV, 3 W power source) were transmitted through a 0.4 mm thick stack BiOI crystals, whereas 78% was transmitted through Si of the same thickness (**Figure** **11**a,b).^[^ ^54^ ^]^ Overall, Bi‐based vacancy‐ordered triple perovskites have similar linear attenuation coefficients as LHPs, whilst BiI_3_ and BiOI have larger values. CdTe has linear attenuation coefficients exceeding all materials apart from BiOI at photon energies below 100 keV, but falls below LHPs and PIMs at higher energies (Figure 7a). But due to the higher mass density of CdTe (5.85 g cm^−3^) than LHPs and many PIMs, the overall attenuation efficiency of CdTe is higher than all materials apart from BiOI (Figure 7b,c). All materials have linear attenuation coefficients exceeding α‐Se. To put this in context, 90% attenuation of 30 keV X‐rays can be achieved with approximately 400 µm of MAPbI_3_ (Rb_3_Bi_2_I_9_^[^ ^51^ ^]^ and (NH_4_)_3_Bi_2_I_9_ have similar values), 180 µm of CdTe, and only 134 µm BiOI (Figure 7b).^[^ ^79^ ^]^ For 667 keV γ‐ray photons, the attenuation efficiency is substantially increased in MAPbI_3_ over α‐Se, and approaches that of CdTe, but BiOI still has by far the largest attenuation efficiency (Figure 7c). These illustrative examples emphasize the benefits of using radiation detector materials with high fractions of Bi combined with other heavy elements (such as I), and with a high mass density.

##### Radiation Hardness

Beyond the high stopping power of Bi‐based PIMs, they have also been shown to have high radiation hardness. A wide range of exposure doses have been investigated, from 9.2 Gy_air_ X‐ray exposure (for Cs_2_AgBiBr_6_) to 480 000 Gy_air_ γ‐ray exposure (for Rb_3_Bi_2_I_9_).^[^ ^51^, ^78^ ^]^ 9.2 Gy_air_ X‐ray exposure is already 92 000 times the dose required for a chest X‐ray,^[^ ^152^ ^]^ and 480 000 Gy_air_ γ‐rays is equivalent to 10 million CT scans.^[^ ^51^ ^]^ In the cases of Cs_2_AgBiBr_6_ (9.2 Gy_air_, X‐rays, 30 keV peak intensity),^[^ ^78^ ^]^ AgBi_2_I_9_ (58 Gy_air_, X‐rays, 43 keV mean energy) and Rb_3_Bi_2_I_9_ (5000 Gy_air_, ^60^Co γ‐rays),^[^ ^51^, ^152^ ^]^ there was little to no change in the dark current, sensitivity, SNR, or photocurrent of the detectors (see Figure 11e for the example of AgBi_2_I_7_). For Rb_3_Bi_2_I_9_ single crystal detectors, as the total dose of γ‐rays was increased to 480 000 Gy_air_, the dark current only increased from 1.17 pA to 9.53 pA.^[^ ^51^ ^]^ The origin of this high radiation tolerance has not been thoroughly studied, but may occur for similar reasons to their LHP counterparts (see Section 3.1.2). It is notable that all of the stability tests were conducted with the Bi‐based PIM single crystals held unencapsulated in ambient air. All materials were found to stable when stored in ambient air, and exhibit higher thermal stability than LHPs.^[^ ^51^, ^78^, ^79^, ^152^ ^]^ These properties have generally been found among inorganic Bi‐based PIMs. For example, we found BiOI single crystals to retain the same phase after storage in dry air for 3 years. BiOI is tolerant to percent‐level intentional changes in surface composition,^[^ ^205^ ^]^ and therefore may also maintain its performance under harsh operating conditions.

#### 3.2.2 Low Dark Currents

##### Effects of Dimensionality

Bi‐based PIMs have exhibited LoDDs well below the values for α‐Se and CZT, and are comparable to or lower than the values reported for LHP single crystals. This is due to the high stopping power (see Section 3.2.1), large *µτ* products (in the range of 10^−3^–10^−2^ cm^2^ V^−1^ in most cases, see Table 3) and low dark current densities achievable in Bi‐based PIMs. The low dark current densities are partly due to the wide bandgaps of these materials (on the order of 2 eV or larger), as well as their low electronic dimensionality, which lead to higher resistivities. For example, (NH_4_)_3_Bi_2_I_9_ is a layered material, and these single crystals were grown with sufficiently large thickness to enable characterization in the in‐plane and out‐of‐plane directions. Higher resistivities were obtained in the out‐of‐plane direction, leading to lower LoDD values down to 55 nGy_air_ s^−1^ (whereas the in‐plane direction LoDD was 210 nGy_air_ s^−1^).^[^ ^79^ ^]^ At the same time, the out‐of‐plane direction also yielded lower sensitivities of 803 µC Gy_air_^−1^ cm^−2^ (compared to 8000 µC Gy_air_^−1^ cm^−2^ in‐plane). This difference was in‐part due to the out‐of‐plane direction having an order of magnitude lower mobility of 11 cm^2^ V^−1^ s^−1^ (compared to 213 cm^2^ V^−1^ s^−1^ in‐plane), leading to lower charge collection.^[^ ^79^ ^]^

The best LoDD values have been obtained from low‐dimensional Bi‐based PIMs, including 0D MA_3_Bi_2_I_9_ and Cs_3_Bi_2_I_9_, and 2D Cs_3_Bi_2_Br_9_, Rb_3_Bi_2_I_9_ and BiOI.^[^ ^51^, ^144^, ^146^ ^]^ The lowest directly measured LoDD is as low as 1.2 nGy_air_ s^−1^ for Cs_3_Bi_2_Br_9_ single crystals (Figure 11f).^[^ ^146^ ^]^ If this were extrapolated down to an SNR of 3, the LoDD would then be 0.58 nGy_air_ s^−1^ (Figure 11f).^[^ ^53^, ^126^, ^128^ ^]^ In all cases, the resistivities were high and dark current densities low, in the range of 10^11^–10^12^ Ω cm, and 3–27 pA mm^−2^, respectively, at the applied biases used to measure the LoDD.^[^ ^51^, ^142^, ^144^, ^146^ ^]^ These detectors therefore come close to fulfilling the 1 pA mm^−2^ requirement for achieving the maximum linear dynamic range,^[^ ^69^ ^]^ exceeding the performance of LHP radiation detectors. The LoDD values achieved with Bi‐based PIMs also outperform the best scintillator detectors (13 nGy_air_ s^−1^).^[^ ^51^ ^]^

##### Low‐Defect Densities in Single Crystals

Importantly, Bi‐based PIMs can be grown as single crystals with low defect densities in the range of 10^9^–10^11^ cm^−3^.^[^ ^51^, ^78^, ^79^, ^144^, ^146^ ^]^ These are important for achieving high resistivities, and can be achieved from solution growth of the single crystals, or from chemical vapor transport (CVT). An important parameter to minimize the defect density in these crystals is the cooling rate. For example, solution‐grown Rb_3_Bi_2_I_9_ needed to be cooled at a low rate of 1 ˚C h^−1^ to ensure low trap densities of 8.43 × 10^10^ cm^−3^.^[^ ^51^ ^]^ Similarly, BiOI grown by CVT needed to be cooled at <0.1 ˚C min^−1^ in order to obtain a low trap density of 2.3 × 10^9^ cm^−3^. Cooling at a higher rate led to lower optoelectronic quality, with a weaker PL signal.^[^ ^54^ ^]^ For Cs_3_Bi_2_Br_9_ single crystals grown by the vertical Bridgman method, reducing the growth rate from 3 mm h^−1^ to 1 mm h^−1^ led to a reduction in the trap density (from 65.6 × 10^8^ to 9.96 × 10^8^ cm^−3^) and an increase in resistivity (from 6.67 × 10^11^ to 14.1 × 10^11^ Ω cm). These factors contributed to the ultralow LoDD values obtained in Cs_3_Bi_2_Br_9_ crystals (1.2 nGy_air_ s^−1^ directly measured, 0.58 nGy_air_ s^−1^ extrapolated; Figure 11f) for high energy (120 keV) X‐rays.^[^ ^146^ ^]^

##### Controlling Surface Traps

Despite the low bulk defect density, it is still important to clean the surfaces of Bi‐based PIM crystals to achieve a low surface recombination velocity. For example, as‐grown Cs_2_AgBiBr_6_ single crystals had a defect density of 4.54 × 10^9^ cm^−3^, with a low mobility of 3.17 cm^2^ V^−1^ s^−1^. Annealing these crystals in an N_2_‐filled glovebox for 2 h led to a reduction in the trap density to 1.74 × 10^9^ cm^−3^, along with an improvement in mobility to 11.81 cm^2^ V^−1^ s^−1^.^[^ ^78^ ^]^ However, the surface recombination velocity was found to increase from 1496 cm s^−1^ to 4144 cm s^−1^ with thermal annealing. It was only by washing the surface with isopropanol or ethyl acetate that the surface recombination velocity reduced to 36.9 cm s^−1^.^[^ ^78^ ^]^ We have found washing the surface of BiOI single crystals (by rinsing in isopropanol, followed by degassing under vacuum for 2 h) to also be important to minimize dark current densities. Similarly, (NH_4_)_3_Bi_2_I_9_ single crystals were wiped and dried overnight in an N_2_‐filled glovebox, and annealed at 100 ˚C for 2 h to remove moisture from the surface and relieve lattice stress.^[^ ^79^ ^]^

##### Polycrystalline Wafers

**Figure 12:**
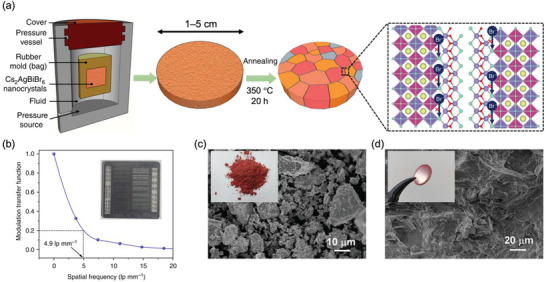
Bi‐based perovskite‐inspired material detectors, made from polycrystalline wafers. a) Illustration of the preparation of Cs_2_AgBiBr_6_ wafers from powders through isostatic pressing, followed by annealing. The surface of Cs_2_AgBiBr_6_ is heteroepitaxially passivated by BiOBr. b) Measurement of the spatial resolution of Cs_2_AgBiBr_6_ X‐ray imagers made from polycrystalline wafers. Parts (a) and (b) reproduced under the terms of the CC‐BY license.^[^ ^78^ ^]^ Copyright 2019, The Authors. Published by Springer Nature. Scanning electron microscopy (SEM) images and photographs (inset) of c) loose powders and d) pressed pellets of (MA)_3_Bi_2_I_9_. Parts (c) and (d) reproduced with permission.^[^ ^141^ ^]^ Copyright 2020, Wiley‐VCH GmbH.

Single crystals are limited by their small size (typical volumes on the order of mm^3^), or the long time (on the order of 1 week) taken to grow large crystals. For applications in medical imaging, it is therefore critical to focus on approaches to grow materials on the cm^3^ volume rapidly, while preserving the highly desirable properties found in single crystals. The most widely‐investigated approaches are thick film deposition and the fabrication of pressed pellets (also called wafers) from powders of the materials. The latter approach has already been investigated for Bi‐based PIMs. Following their successful demonstration as single crystals, Cs_2_AgBiBr_6_ and MA_3_Bi_2_I_9_ were recently investigated as polycrystalline wafers (**Figure** **12**).^[^ ^69^, ^141^ ^]^ These add to the work building off the older literature on wafers of Bi‐based compounds, such as BiOI and BiSI.^[^ ^203^, ^206^ ^]^ But the report on BiOI wafers had a sensitivity <1 µC Gy_air_^−1^ cm^−2^,^[^ ^206^ ^]^ well below the sensitivity of 1100 µC Gy_air_^−1^ cm^−2^ we recently achieved from BiOI single crystals.^[^ ^54^ ^]^ By contrast, the more recent reports of Cs_2_AgBiBr_6_ and MA_3_Bi_2_I_9_ wafers (both pressed at 0.2 GPa—see process in Figure 12a) achieved LoDDs and sensitivities approaching those of their single crystal counterparts. This was partly because of the high resistivities achieved in these wafers, in the range of 10^10^–10^11^ Ω cm.^[^ ^69^, ^141^ ^]^ The MA_3_Bi_2_I_9_ wafer had a low dark current density of 20 pA mm^−2^, along with densities reaching 97% those of MA_3_Bi_2_I_9_ single crystals.^[^ ^141^ ^]^ The compact morphology of MA_3_Bi_2_I_9_ wafers compared to their starting loose powders is shown in Figure 12c,d. The density of the Cs_2_AgBiBr_6_ wafer was not reported, but it had a compact morphology with large grains 100 nm to 1 µm in size due to its post‐annealing treatment at 350 °C for 20 h to promote grain growth.^[^ ^69^ ^]^ These factors would have ensured the wafers had a high linear attenuation coefficient, along with *µτ* products (10^−5^ cm^2^ V^−1^ for MA_3_Bi_2_I_9_, 10^−3^ cm^2^ V^−1^ for Cs_2_AgBiBr_6_) that were not severely reduced compared to those of their single crystal counterparts (10^−3^ cm^2^ V^−1^ for MA_3_Bi_2_I_9_, 6.3 × 10^−3^ cm^2^ V^−1^ for Cs_2_AgBiBr_6_), enabling their high performance.^[^ ^69^, ^78^, ^141^, ^142^ ^]^

In addition to efficient X‐ray detection, Cs_2_AgBiBr_6_ wafers also demonstrated promising initial spatial resolutions reaching 4.9 lp mm^−1^ at 20% modulation transfer function (Figure 12b).^[^ ^78^ ^]^ Although this is below the highest spatial resolution reported for LHP single crystals, it exceeds the spatial resolution reported for thick perovskite films (3.1 lp mm^−1^).^[^ ^81^ ^]^ The higher spatial resolution of the Cs_2_AgBiBr_6_ wafers was partly due to compact morphology, small pixel size used (200 µm × 200 µm), and low dark current densities. We note that an important factor behind the low dark current densities was the heteroepitaxial passivation with BiOBr on the surface,^[^ ^78^ ^]^ and this is discussed more in Section 4.1.

#### 3.2.3 High Activation Energy Barriers to Ion Migration

**Figure 13:**
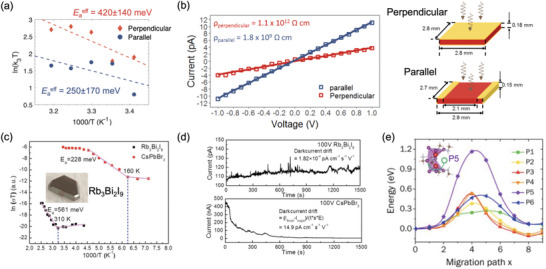
Resilience against ion migration in Bi‐based perovskite‐inspired material detectors. a) Arrhenius plots to determine the effective activation energy barrier to ion migration (*E*_a_^eff^) for BiOI single crystals, and b) dark current–voltage curves of BiOI photoconductors in the perpendicular and parallel configurations (illustrated on the right). Parts (a) and (b) reproduced under the terms of the CC‐BY license.^[^ ^54^ ^]^ Copyright 2023, The Authors. Springer Nature. c) Arrhenius plots to determine the activation energy barrier to ion migration (*E*_a_) in Rb_3_Bi_2_I_9_ *vs*. CsPbBr_3_ single crystals (photograph of Rb_3_Bi_2_I_9_ crystal inset). d) Dark current drift for Rb_3_Bi_2_I_9_ *vs*. CsPbBr_3_ single crystals with 100 V applied bias. Parts (c) and (d) reproduced with permission.^[^ ^51^ ^]^ Copyright 2020, Wiley‐VCH GmbH. e) Calculated activation energy barrier to I^−^ ion migration in (MA)_3_Bi_2_I_9_. Path for I^−^ along P5 shown inset. Reproduced with permission.^[^ ^210^ ^]^ Copyright 2020, CellPress.

LHPs are prone to ion migration when an electric field is applied, limiting their polarization stability (see Section 5.2 for a more detailed discussion).^[^ ^82^ ^]^ Bi‐based PIMs, on the other hand, have demonstrated greater stability with the application of an electric field, especially in low‐dimensional structures. This is because of their higher activation energy barriers (*E*_A_) to ion migration, exceeding 300 meV in many cases, which are greater than the *E*_A_ values reported for MAPbI_3_ (137–190 meV),^[^ ^51^, ^207^ ^]^ MAPbBr_3_ (126–168 meV)^[^ ^78^, ^207^, ^208^ ^]^ and CsPbBr_3_ (228 meV).^[^ ^51^ ^]^ *E*_A_ depends on formation energy of these defects, and the activation energy barrier for the defects to move across different lattice sites.^[^ ^209^ ^]^ Thus, reducing the electronic dimensionality to impede field‐induced defect migration can increase *E*_A_. Indeed, we have found that *E*_A_ is larger in the out‐of‐plane direction in BiOI (*E*_A_ = 350±50 meV to 420±140 meV, compared to 250±170 meV in‐plane) because it is more difficult for ions to migrate between planes (**Figure** **13**a).^[^ ^54^ ^]^ The in‐plane *E*_A_ of 250±170 meV is similar to that found in CsPbBr_3_ (Figure 13c), likely because the formation energies of iodide or bismuth vacancy defects are low,^[^ ^204^ ^]^ and thus have high concentration. The lower polarization in the in‐plane direction in BiOI single crystals enabled high resistivities reaching up to 10^12^ Ω cm, lower LoDDs and a more stable current baseline in chopped photoconductivity measurements (Figure 13b).^[^ ^54^ ^]^ Similar effects have been found in (NH_4_)_3_Bi_2_I_9_, in which the *E*_A_ was higher in the out‐of‐plane (910 meV) than in‐plane (720 meV) direction, leading to lower dark currents and LoDD values.^[^ ^79^ ^]^

Higher activation energy barriers have been measured in single crystals of 2D Rb_3_Bi_2_I_9_ (561 meV)^[^ ^51^ ^]^ and Cs_3_Bi_2_Br_9_ (1032.4 meV),^[^ ^146^ ^]^ as well as 0D MA_3_Bi_2_I_9_ (460 meV).^[^ ^142^ ^]^ Computations showed that the high *E*_A_ for MA_3_Bi_2_I_9_ arose from the difficulty in I^−^ hopping between adjacent isolated Bi_2_I_9_^3−^ clusters, with a calculated energy barrier as high as 1180 meV (Figure 13e).^[^ ^210^ ^]^ The highly suppressed ion migration in these materials was quantified by measuring how much the dark current changed over time with the application of a constant field. For example, Xia et al. compared the polarization stability of Rb_3_Bi_2_I_9_ and CsPbBr_3_ single crystals, applying 100 V bias across each of them. After 30 min, the drift of the CsPbBr_3_ device was 14.9 pA cm^−1^ s^−1^ V^−1^, whereas the Rb_3_Bi_2_I_9_ device had a five orders of magnitude smaller drift of 1.82 × 10^−4^ pA cm^−1^ s^−1^ V^−1^ (Figure 13d).^[^ ^51^ ^]^

The path for ion migration can be further disrupted and *E*_A_ further increased by moving towards a polycrystalline system. It was shown with both Cs_2_AgBiBr_6_ and MA_3_Bi_2_I_9_ that the *E*_A_ increased from 348 meV to 360 meV (Cs_2_AgBiBr_6_),^[^ ^69^, ^78^ ^]^ and from 460 meV to 480 meV (MA_3_Bi_2_I_9_).^[^ ^141^, ^142^ ^]^ At the same time, it is important to ensure that the grain boundaries and surfaces are well passivated to prevent them from acting as sites for fast ionic transport. For example, without heteroepitaxial passivation from BiOBr, polycrystalline wafers of Cs_2_AgBiBr_6_ had a lower *E*_A_ of 203 meV.^[^ ^69^ ^]^ Computational investigations showed that the barrier to bromide vacancy diffusion was lower at the surface (250 meV) than in the bulk (300 meV) of Cs_2_AgBiBr_6_, but can be greatly enhanced to 440 meV when passivated with BiOBr.^[^ ^69^ ^]^

#### 3.2.4 Carrier Localization

Finally, an important disadvantage found in many bismuth‐halide‐based compounds is the strong coupling between charge‐carriers and optical or acoustic phonons. This can severely reduce *µτ* products by i) limiting mobilities, and ii) creating new non‐radiative loss channels that will still be present in a defect‐free material. Such strong carrier‐phonon coupling is not present in bulk 3D LHPs, but has been so widely found in Bi‐halide compounds that it has been labeled a hallmark of these materials.^[^ ^189^, ^211^, ^212^ ^]^ This arises because of the soft, polar lattices (increasing the coupling to longitudinal optical [LO] phonons), and low electronic dimensionality found in many materials (reducing or eliminating the energy barrier to carrier localization). Coupling between free carriers and LO phonons (called Fröhlich coupling) severely reduces the mobility. If Fröhlich coupling is very strong, or if the coupling to acoustic phonons is strong, carrier localization will occur. This describes the reduction in the spatial extent of the electron wavefunction to the order of one unit cell or lower, meaning that electrons can no longer freely move, but needs to hop between sites.^[^ ^189^ ^]^ Carrier localization can occur with free carriers (small electron or hole polarons) or excitons (self‐trapped excitons).

**Figure 14:**
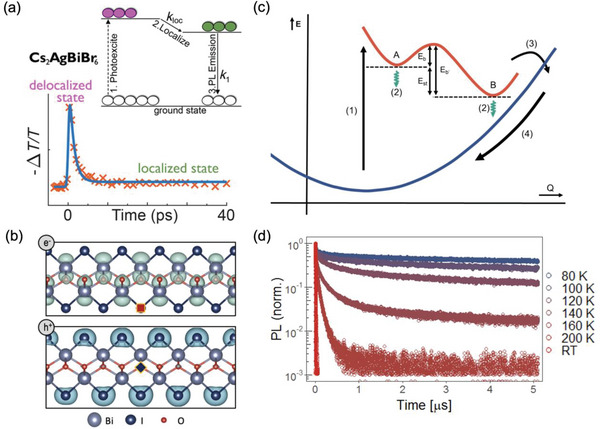
a) Optical pump terahertz probe spectroscopy measurements, which reveal a rapid decrease in photoconductivity. These measurements were fit with a two‐level model, shown inset, from which the carrier localization rate (*k*_loc_) was found to be 0.99±0.43 ps^−1^. Reproduced under the terms of the CC‐BY license.^[^ ^213^ ^]^ Copyright 2021, The Authors. Published by American Chemical Society. b) Spatial distribution of the electron (top; with hole, red square, fixed on iodine) and hole (bottom; with electron, blue diamond, fixed on oxygen) components of the lowest‐lying direct exciton of BiOI. c) Proposed configuration coordinate diagram for BiOI based on ultrafast spectroscopy measurements and computations. Process (1) is photo‐excitation, (2) is the coupling of excited‐state carriers to the ground state, giving off photoluminescence, (3) the direct entry of excited‐state carriers to the ground state, and (4) non‐radiative relaxation of the lattice. Processes (3) and (4) lead to the non‐radiative loss of photo‐excited carriers. d) Time‐resolved photoluminescence decay of BiOI at room temperature, and down to 80 K. Parts (b–d) reproduced under the terms of the CC‐BY license.^[^ ^54^ ^]^ Copyright 2023, The Authors. Published by Springer Nature.

For instance, there have been many detailed studies into Cs_2_AgBiBr_6_ double perovskite.^[^ ^212^ ^]^ Despite having 3D structural dimensionality, the electronic dimensionality is much lower because of the energetic mismatch of the Ag 4d and Bi 6s/6p frontier orbitals.^[^ ^214^ ^]^ Herz and co‐workers found from optical pump terahertz probe measurements that the mobility rapidly decreases with a localization rate of 0.99 ± 0.43 ps^−1^, which is too fast to be due to defect trapping (**Figure** **14**a).^[^ ^213^ ^]^ Wu et al. found that carrier localization occurs in Cs_2_AgBiBr_6_ not because of Fröhlich interactions (since the coupling constant is similar to LHPs), but because of coupling to acoustic phonons, which is strong because of the high deformation potential of 13.7 eV (at the valence band maximum) and 14.7 eV (conduction band minimum), whereas LHPs have smaller deformation potentials (2.2–6.3 eV).^[^ ^188^ ^]^ The acoustic coupling constant is proportional to the square of the deformation potential, and high acoustic coupling constants can lead to self‐trapping.^[^ ^212^ ^]^ As a result, the mobilities in Cs_2_AgBiBr_6_ single crystals only reach up to 11.81 cm^2^ V^−1^ s^−1^ with *µτ* products up to 6.3 × 10^−3^ cm^2^ V^−1^,^[^ ^78^ ^]^ whereas LHP single crystals have mobilities reaching to the hundreds of cm^2^ V^−1^ s^−1^,^[^ ^12^ ^]^ and *µτ* products beyond 10^−2^ cm^2^ V^−1^ (Table 3). Self‐trapping has also been found in AgBiI_4_ and NaBiS_2_, among other materials.^[^ ^21^, ^215^ ^]^

Recently, we have found BiOI to deviate from the bismuth‐halide compounds explored thus far, and have optoelectronic properties that could be fully described by Fröhlich coupling without invoking self‐trapping. Our computational studies showed that the lowest‐energy exciton formed following photoexcitation has electron and hole wavefunctions that are delocalized across the entire I‐Bi‐O‐Bi‐I plane (Figure 14b). This leads to high in‐plane mobilities reaching up to 83 cm^2^ V^−1^ s^−1^. Lal et al. also recently found from optical‐pump terahertz probe spectroscopy measurements that BiOI avoids carrier localization.^[^ ^216^ ^]^ At the same time, Fröhlich coupling in BiOI leads to irreversible reductions in the charge‐carrier lifetime, such that the room‐temperature PL lifetime is only 1.8 ns (Figure 14d).^[^ ^54^ ^]^ This is similar to the PL lifetime we previously measured for BiOI thin films.^[^ ^204^ ^]^ We found through ultrafast spectroscopy measurements and computations that following photoexcitation, impulsive absorption leads to the propagation of two dominant LO phonon modes, with wavenumbers 86 cm^−1^ and 152 cm^−1^ (Figure 14c,d). These two *A*_1g_ modes lead to a distortion of the lattice, such that the excited state forms two local energy minima, at which excited‐state charge‐carriers relax to and couple to the ground state, giving off a red‐shifted, double‐peak PL spectrum (Figure 14c). At room temperature, there is sufficient thermal energy to adequately populate these phonon modes, such that the excited state and ground state approach energy other in energy, allowing excited‐state charge‐carriers to enter into the ground state and non‐radiatively relax in energy. This forms an unavoidable loss channel that will still be present even when the defect densities are as low as they are in the single crystals (2.3 × 10^9^ cm^−3^), thus explaining why the single crystals have the same PL lifetime as thin films. Importantly, we showed that the PL lifetime increased from 1.8 ns at room temperature to 6.8 µs at 80 K because the two *A*_1g_ phonon modes were sufficiently depopulated that the lattice could not distort such that the excited and ground states approached each other, and we therefore suppressed this loss channel (Figure 14c). Apart from reducing thermal energy, we showed that we could also suppress this non‐radiative loss channel by applying an electric field. We believe that this allowed charge‐carriers to be decoupled from distortions in the lattice, such that much longer drift lifetimes are obtained. We measured the in‐plane *µτ* product to be (6±2) × 10^−2^ cm^2^ V^−1^, which means that a drift length of 1.5 mm could be obtained with 5 V applied bias (24 V cm^−1^), whereas the diffusion length could only reach up to a maximum of 0.4 µm at room temperature. This allowed measurable photocurrents to be obtained from the single crystals on the mm‐lengthscale. Note that lower *µτ* products of (1.1±1.4) × 10^−3^ cm^2^ V^−1^ were obtained from the out‐of‐plane direction due to lower mobilities, but this configuration gave the highest sensitivities and lowest LoDDs because of suppressed ion migration.^[^ ^54^ ^]^

It will therefore be important to understand how carrier localization could be avoided in Bi‐based PIMs to design materials with high detector performance, as well as to control the degree of Fröhlich coupling to increase *µτ* products.

## 4 Strategies Toward High‐Performance Radiation Detectors

In the previous section, we discussed the key properties that make halide perovskites and their derivatives highly promising as radiation detectors, along with current challenges. This section discusses the strategies to improve their sensitivity, limit of detection, and energy and spatial resolution by fine‐tuning important properties, such as the bandgap, bulk resistivity, trap density, and activation energy for ion migration. In particular, we expand the discussion from single crystals and structurally 3D perovskites through to low‐dimensional structures and form factors. We also discuss novel applications of these detectors in wearables (through flexible devices), and portable devices through self‐powered and low‐bias radiation detectors. This section also points out strategic scalable synthesis routes and device fabrication techniques employed that are suitable for commercialization.

### 4.1 Structurally Low Dimensional Materials

**Figure 15:**
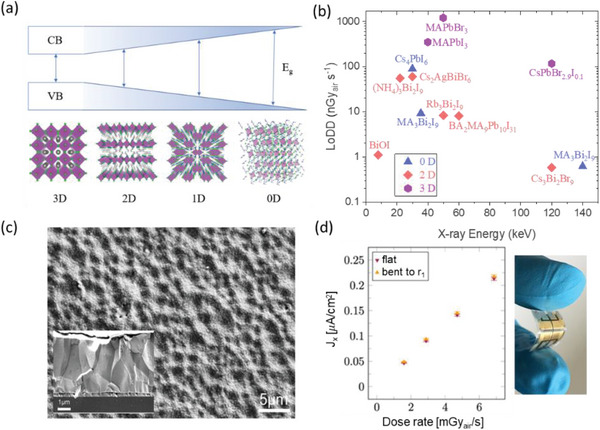
Structurally and morphologically low dimensional materials for radiation detection. a) Schematic showing the widening of the bandgap as the electronic dimensionality in lead‐halide perovskites is reduced. Adapted with permission from Ref. [217] and [218]. Copyright 2011, Wiley‐VCH GmbH and 2019, Elsevier, respectively. b) Detection limit (LoDD) reported for various low dimensional and 3D halide perovskite X‐ray detectors at different X‐ray energies. Data for (b) is extracted from Ref. [51, 78, 79, 95, 98, 141, 142, 146, 219, 220, 221, 222] c) Top‐view scanning electron microscopy (SEM) image of inkjet‐printed perovskite QD film, with the cross‐section inset. d) Dark current corrected X‐ray induced current density at varying X‐ray dose rates under flat and bent (*r*_1_ ≈ 9 mm) conditions, for a perovskite QD‐based flexible X‐ray detector. Image of this device shown on the right. Parts (c) and (d) adapted with permission.^[^ ^223^ ^]^ Copyright 2020, American Chemical Society.

Structurally low dimensional (LD) halide perovskites are advantageous because of their stable performance when used as radiation detectors, owing to their large activation energies for ion migration that arise from their high formation energies for ion vacancies, which hinders dark current drift under applied bias.^[^ ^142^, ^224^ ^]^ Although high *Z*_eff_ is needed to achieve high stopping power,^[^ ^225^ ^]^ increases in *Z*_eff_ also lead to larger wavefunction overlap between adjacent atoms, causing band broadening and reductions in the bandgap.^[^ ^225^ ^]^ Reducing the structural dimensionality from 3D to 0D can minimize this wavefunction overlap and lead to a wider bandgap (**Figure** **15**a).^[^ ^217^ ^]^ Wide bandgaps in LD halide perovskites minimize the thermal carrier concentration and thereby reduce dark current, and their high resistivities along certain crystallographic directions effectively suppress the noise current when operated under a large applied bias.^[^ ^51^, ^146^ ^]^

Owing to these qualities, LD perovskites are especially promising candidates for detecting radiation with energy exceeding 100 keV, where the attenuating medium must be thicker (of ≈mm order) and needs to be operated at high biases to achieve efficient charge‐carrier collection.^[^ ^146^ ^]^ Zheng et al. reported an organic‐inorganic 0D vacancy‐ordered triple perovskite, MA_3_Bi_2_I_9_, with a high activation energy barrier to ion migration (*E*_A_ = 400 meV) and low dark current concentration of ≈10^6^ cm^−3^, which resulted in a high‐performance radiation detector with a sensitivity of 10 620 µC Gy_air_^−1^ cm^−2^ at a high bias voltage of 120 V and an extrapolated LoDD of 0.62 nGy_air_ s^−1^.^[^ ^142^ ^]^ Later Li et al. reported an all‐inorganic two‐dimensional (2D) A_3_B_2_X_9_ material, Cs_3_Bi_2_Br_9_, for 120 keV hard radiation detection, which exhibited a decent sensitivity of 1705 µC Gy_air_^−1^ cm^−2^ and an extrapolated LoDD of 0.58 nGy_air_ s^−1^ due to an ultra‐low dark current density of 0.35 pA mm^−2^ even at an applied bias of 1000 V.^[^ ^146^ ^]^ Owing to an ultra‐high ion activation energy of 1.03 eV, these devices achieved very low dark current drift of only 2.8 × 10^−10^ nA cm^−1^ s^−1^ V^−1^, resulting in exceptional operational stability.^[^ ^146^ ^]^

Quasi‐2D Ruddlesden‐Popper (RP; general formula: A’_2_A*_n_*_‐1_B*_n_* X_3_ *_n_*_‐1_) and Dion‐Jacobson (DJ; general formula: A’’A*_n_*_‐1_B*_n_* X_3_ *_n_*_‐1_) perovskites have also been investigated for their suitability as radiation detectors.^[^ ^220^, ^226^, ^227^, ^228^ ^]^ A’ is a monocation, and A’’ a dication‐based organic spacer. The advantage of RP and DJ perovskites lies in the tunability of the number of inorganic perovskite layers (*n*) which enables the material properties to be finely tuned. As a reduction in *n* is associated with a decrease in dimensionality, it results in increases in resistivity and the activation energy barrier for ion migration. Zhang et al. studied RP BA_2_MA*_n_*_‐1_Pb*_n_* I_3_ *_n_*_‐1_ and tuned *n* values.^[^ ^220^ ^]^ As *n* changed from 40 to 1, both *E*_A_ and resistivity increased. However, the charge‐carrier mobility decreased, and the optimal value of *n* was found to be 10, leading to the best‐performing detectors with a sensitivity of 5362.3 µC Gy_air_^−1^ cm^−2^ at 100 V and LoDD of 8.1 nGy_air_ s^−1^.^[^ ^220^ ^]^ On the other hand, DJ perovskites can have higher µτ products than RP perovskites, owing to suppressed electron‐phonon coupling due to their lattice rigidity, which is crucial for excellent carrier transport.^[^ ^229^ ^]^ Zhang et al. demonstrated this in their work, where changing from RP perovskite (o‐F‐PEA)_2_PbI_4_ (o‐F‐PEA = ortho‐fluoro‐phenethylamine) to a DJ perovskite (DGA)PbI_4_ (DGA = dimethylbiguanide) led to a reduction in the Fröhlich coupling constant (γ_*LO*_) from 70 meV to 58.1 meV. As a result, the sensitivity increased by nearly a factor of three to 4689 µC Gy_air_^−1^ cm^−2^ at 12 kV cm^−1^.^[^ ^228^ ^]^ Although DJ perovskites are promising for radiation detection, there have only been limited studies on these.^[^ ^227^, ^228^ ^]^

While 0D and 2D halide perovskites have proven their promise for direct radiation detection, 1D halide perovskites have been more investigated as scintillators. To our knowledge, there are no reports of lead‐based 1D halide perovskites for direct radiation detection.^[^ ^230^, ^231^, ^232^ ^]^ A handful of research groups have studied the performance of lead‐free (mostly Bi‐based) molecular 1D halide perovskites for direct X‐ray detection.^[^ ^233^, ^234^, ^235^, ^236^, ^237^ ^]^ Ma et al. demonstrated highly water‐resistant benzamidinium (BAH) based 1D (BAH)BiI_4_ single crystals, where negligible decomposition was observed even after soaking these crystal powders in water for 60 days.^[^ ^235^ ^]^ The X‐ray sensitivity (for 40 kVp X‐rays) of these devices was 1181.8 µC Gy_air_^−1^ cm^−2^ with a detection limit lower than 72 nGy_air_ s^−1^. Even when (BAH)BiI_4_ single crystal devices were soaked in water for 2 h, the sensitivity was retained at 801.8 µC Gy_air_^−1^ cm^−2^, without compromising on the device's linear response in the 0.2‐1.6 µGy_air_ s^−1^ dose rate range. Cui et al. proposed a metal‐free 1D halide perovskite DABCO‐NH_4_I_3_ (DABCO = N‐N’‐diazabicyclo[2.2.2]octonium) as a bio‐friendly alternative to heavy metal based radiation detectors (see Table 3).^[^ ^155^ ^]^ Owing to an excellent in‐plane carrier mobility of 110 cm^2^ V^−1^ s^−1^ along the 1D chains in this material, X‐ray detectors with a planar configuration exhibited a good sensitivity of 567 µC Gy_air_^−1^ cm^−2^ for 29 keV X‐rays (Table 3), which is comparable to other metal‐based halide perovskites, such as MAPbBr_3_ and Cs_2_AgBiBr_6_.^[^ ^78^, ^99^, ^155^ ^]^

### 4.2 Morphologically Low Dimensional Materials

As discussed in Section 3.1, LHPs have desirable bulk properties, but are still limited by non‐radiative recombination at surfaces and interfaces. Recently, morphologically low‐dimensional LHPs, such as 2D nanostructures (nanosheets and nanoplates), 1D nanostructures (nanowires, nanoribbons, and nanorods), and 0D nanostructures (quantum dots, nanocrystals, and nanoparticles) have been employed as potential building blocks to construct efficient radiation detectors. In general, low‐dimensional nanostructures have a tendency to be single crystalline within each nanostructure. They therefore have the advantages of single crystals, such as low non‐radiative recombination rates and low concentrations of ionic defects. At the same time, low‐dimensional nanostructures exhibit superior optical and mechanical properties for flexible devices compared to single crystals.^[^ ^238^ ^]^ They can also have tunable bandgaps, as well as high PLQYs, large extinction coefficients, and multiple exciton generation.^[^ ^239^ ^]^ All of these characteristics allow them to show higher performance reliability and signs of better stability than their polycrystalline counterparts, in terms of suppressed ion migration, environmental stability, phase stability, and mechanical stability.

Halide perovskite nanocrystals (NCs) and quantum dots (QDs) have been extensively investigated for scintillators, owing to their large Stokes shift, high PLQYs, and fast response, but studies on their applicability as direct detectors are still limited.^[^ ^240^, ^241^, ^242^, ^243^ ^]^ Solution‐processed halide perovskite NCs and QDs are suitable for flexible and large‐area scalable radiation detector applications, since they can be conveniently printed via cost‐effective and high throughput methods (e.g., inkjet printing) onto a wide variety of substrates, including flexible polymers and paper.^[^ ^97^, ^223^, ^244^ ^]^ It is important to be able to control the thickness of the films deposited, since thick films (ideally on the mm‐scale or larger) are required for the high attenuation of ionizing radiation (Figure 7b,c), but thinner films are preferable for improved mechanical flexibility.^[^ ^244^ ^]^ As these perovskite NCs and QDs can form uniform films of up to a maximum thickness of a few tens of microns (Figure 15c), they are only appropriate for soft X‐ray applications, such as mammography. They are also an excellent choice for flexible, low operating voltage radiation detectors which are suitable for wearable and portable applications, such as personal dosimeters.^[^ ^245^ ^]^ A flexible X‐ray detector can follow the contours of an irregular object better than a flat panel detector, enabling superior imaging performance by minimizing artifacts such as vignetting in radiographs.^[^ ^246^ ^]^

Mescher et al. studied triple cation perovskite QDs on flexible polyethylene naphthalate (PEN) foils for detecting 70 kVp X‐rays.^[^ ^223^ ^]^ With an active layer thickness of 3.7 µm achieved through digital inkjet printing, these devices exhibit good mechanical flexibility up to a bending strain of 0.48% with minimal photocurrent deterioration (Figure 15d) and a sensitivity reaching up to 59.9 µC Gy_air_^−1^ cm^−2^ at 0.1 V applied bias. Subsequently, Ciavatti et al. demonstrated a printed flexible 10 µm thick MAPbI_3_ NC films on polyethylene naphthalate (PEN) substrates for detecting 150 kVp X‐rays, with sensitivities reaching up to 494 µC Gy_air_^−1^ cm^−2^ using less than 4 V applied bias, which is the highest sensitivity reported thus far amongst all flexible direct detectors.^[^ ^244^ ^]^ Even with extensive bending (strain exceeding 10%), these devices retained more than 70% of their original sensitivity. These rapid improvements in performance show the great promise for halide perovskite NCs and QDs in printable and flexible radiation detection technologies.^[^ ^97^, ^223^, ^244^ ^]^

However, the challenge of how to effectively integrate nanoparticles into a film and remove the insulating ligands to facilitate charge transport is still an area being addressed. Recently, the development of nanoparticle superlattices has been introduced as a powerful approach to enhancing the properties of the materials for practical applications.^[^ ^247^ ^]^ Successfully employing nanoparticle superlattices as an active layer could bring new advances to the radiation detector research community in the coming time.

### 4.3 Defect Mitigation and Passivation

**Figure 16:**
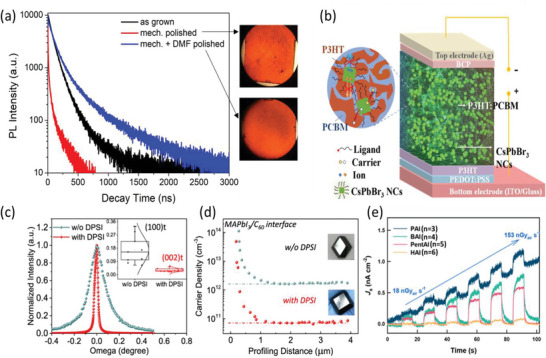
a) Time‐resolved photoluminescence measurements of MAPbBr_3_ single crystals before and after mechanical and mechano‐chemical polishing, along with optical microscopy images of polished crystal surface. Adapted with permission.^[^ ^248^ ^]^ Copyright 2021, Elsevier. b) Illustration of a “perovskite in host” composite X‐ray detector. Reproduced with permission.^[^ ^249^ ^]^ Copyright 2022, Wiley‐VCH GmbH. c) X‐ray rocking curves, with peak FWHM distribution inset, and d) trap density as a function of profiling distance (from top surface) measured using drive‐level capacitance profile technique, for MAPbBr_3_ single crystals grown without and with DPSI ligand. Parts (c) and (d) reproduced under the terms of the CC‐BY license.^[^ ^250^ ^]^ Copyright 2021, The Authors. Published by Springer Nature. e) X‐ray response of Ruddlesden‐Popper perovskites with different A‐site cations. Reproduced with permission.^[^ ^251^ ^]^ Copyright 2022, American Chemical Society.

Earlier in Section 3, we discussed the importance of maintaining low defect densities to achieve high resistivities and suppressed ion migration. Single crystals are a reliable choice for designing radiation detectors with low trap densities (as they are free from grain boundaries, and the defects that can more easily form at grain boundaries), but growing high‐quality single crystals is still challenging.^[^ ^252^ ^]^ Several research groups have developed different strategies to address this challenge, such as controlling the temperature gradient, continuous mass transport processes, low‐temperature metastable crystallization, etc., to hinder the formation of point defects during crystal growth, and consequently improve the performance of the materials in detectors.^[^ ^253^, ^254^, ^255^, ^256^ ^]^ Li et al. grew solution‐processed perovskite single crystals with an ultra‐low trap density of 2.8 × 10^8^ cm^−3^ via solvent‐volatilization‐limited‐growth, where a constant crystal growth rate was maintained to avoid defect formation by modifying the mass transfer dynamics and limiting the solute integration rate onto the growing crystal surface.^[^ ^257^ ^]^ Liu et al. employed a ligand‐assisted perovskite crystal growth using 3‐(decyldimethylammonio)‐propane‐sulfonate (DPSI), where steric hindrance due to the (CH_2_)*_n_*‐ long chain of DPSI played a vital role in significantly improving the crystallinity (**Figure** **16**c) and reducing the density of deep traps (Figure 16d) of single crystals without compromising on their growth rate.^[^ ^250^ ^]^ MAPbI_3_ single crystals grown via this route exhibited a remarkable sensitivity of (2.6± 0.4) × 10^6^ µC Gy_air_^−1^ cm^−2^ and a detection limit of 5.0 ± 0.7 nGy_air_ s^−1^ for 60 kVp hard X‐rays.^[^ ^250^ ^]^

In addition to reducing the bulk defect density, surface and interface defects must also be passivated to minimize surface/interface recombination, as discussed earlier in Section 3.1.5. Liu et al. used UV‐O_3_ treatment on MA_3_Bi_2_I_9_ single crystals and observed oxide formation on the crystal surface due to oxygen chemisorbed onto uncoordinated bismuth at the surface, which resulted in an order of magnitude decrease in dark current density at 2860 V cm^−1^ applied field.^[^ ^258^ ^]^ UV‐O_3_ passivated MA_3_Bi_2_I_9_ radiation detectors exhibited a low detection limit of 31 nGy_air_ s^−1^ and a good spatial resolution of 4.22 lp mm^−1^. Li et al. achieved a sharp reduction in contact/crystal interface defect density from 2.17 × 10^10^ to 8.7 × 10^8^ cm^−2^ in MAPbBr_3_ detectors by simple thermal annealing at 100°C under an inert atmosphere, which resulted in enhanced X‐ray sensitivity.^[^ ^98^ ^]^

Often, halide perovskite single crystals need to be polished to obtain smooth surfaces, and thus maintain a uniform electric field across the detector. However, common mechanical polishing processes can lead to microcracks forming, which can reduce charge‐carrier lifetimes via non‐radiative recombination. Tan et al. demonstrated a mechano‐chemical polishing route using *N*,*N*‐dimethylformamide (DMF) to reduce the surface roughness by a factor of five and eliminate any undesired surface contaminants (e.g., carbon or oxygen). This resulted in significantly improved charge‐carrier lifetime compared to the control sample, which had traditional mechanical polishing (Figure 16a).^[^ ^248^, ^259^ ^]^

### 4.4 Doping and Compositional Tuning

The composition of halide perovskites plays a pivotal role in determining their optoelectronic properties and long‐term stability.^[^ ^260^, ^261^, ^262^ ^]^ Doping or compositional tuning has been proven to be an effective strategy for controlling the bandgap, phase stability, charge‐carrier dynamics, ionic conductivity, and even deep‐level defect concentration,^[^ ^263^, ^264^, ^265^, ^266^, ^267^ ^]^ and some of these are discussed in Section 3.1.4.^[^ ^235^, ^236^, ^237^, ^238^, ^239^ ^]^ The properties required for high‐performance radiation detection, like high mobility‐lifetime products, high activation energy barrier for ion migration, low trap density, low dark current, and noise, can all be realized through compositional engineering.^[^ ^100^, ^123^, ^268^, ^269^ ^]^ Xin et al. performed A‐site doping in RP A_2_MA_9_Pb_10_I_31_ perovskites using alkylamine cations of different carbon chain lengths (with the number of carbon atoms ‘*m*’ varying from 3 to 6), and this simultaneously reduced ion migration and improved µτ products.^[^ ^251^ ^]^ However, it was found that as *m* increased, there was also a decrease in charge‐carrier mobility µ, leading to a trade‐off between polarization stability and detector performance. Nevertheless, a balance between high *E*_A_ and µ was achieved with *m*  =  4, and these materials led to detectors with an LoDD of 7.8 nGy_air_ s^−1^ and sensitivity of ≈7000 *µ*C Gy_air_^−1^ cm^−2^ for 45 keV X‐rays.^[^ ^251^ ^]^ Figure 16e shows the influence of A‐site doping on X‐ray response in these detectors.

Halide doping is a promising route to fine‐tune the bandgap of perovskites, as their valence p orbitals are major contributors to the formation of the band extrema.^[^ ^260^, ^270^ ^]^ Precise anion mixing also allows the suppression of ion migration, increased bulk resistivity, increased charge‐carrier mobility, and improved phase stability.^[^ ^100^, ^266^, ^267^ ^]^ Different research groups have rationalized this enhancement in properties due to anion engineering using various approaches.^[^ ^100^ ^]^ Rybin et al. showed that in MAPbBr_3−_ *_x_* Cl*_x_*_,_ an increase in Cl content leads to a decrease in the effective mass for both electrons ($m_{e}^{*}$) and holes ($m_{h}^{*}$), leading to enhanced charge‐carrier mobilities.^[^ ^266^ ^]^ In studies conducted by Guillen et al., Cl doping in MAPbBr_3_ single crystals leads to improved Pb to halide compositional ratio (confirmed from EDX analysis), leading the crystal structure more towards ABX_3_ stoichiometry.^[^ ^269^ ^]^ They propose that such improvement in stoichiometry can lead to a reduced concentration of point defects such as halide vacancies, which can thereby reduce dark current drift and improve the operational stability of the radiation detector.^[^ ^269^ ^]^ In all of these studies, radiation detectors with improved performance over control MAPbBr_3_ devices were achieved from mixed anion MAPbBr_3−_ *_x_* Cl*_x_* compared, thus emphasizing the potential of doping or compositional tuning to engineer high performance radiation detectors.

### 4.5 Composites

Making composites comprised of halide perovskites and other organic or inorganic semiconductors is an excellent route to achieve suppressed ion migration and efficient charge‐carrier separation, whilst overcoming challenges with scalability and commercialization.^[^ ^249^, ^271^, ^272^, ^273^ ^]^ A few research groups have employed the “perovskite‐in‐host” approach, in which the perovskite is embedded in an organic semiconductor. The perovskite acts as an ionzing radiation photon sensitizer, while the organic semiconductor plays the role of charge transport medium. Wei et al. embedded CsPbBr_3_ NCs into an organic P3HT‐PCBM matrix, where both P3HT and PCBM form type II energy band alignment, with CsPbBr_3_ effectively promoting spontaneous separation of photogenerated charge‐carriers from CsPbBr_3_ NCs into the organic matrix, improving charge collection efficiency.^[^ ^249^ ^]^ In addition, the organic matrix acts as a physical barrier between the NCs and impedes ion migration, thus allowing stable device performance, with a dark current relative drift of only 3.57 × 10^–9^ cm s^–1^ V^–1^ even when operated at a high electric field of 5100 V cm^−1^. Figure 16b shows a schematic of the device structure of these nanocomposite films‐based direct radiation detectors which exhibited an excellent sensitivity of 5696 µC Gy_air_^−1^ cm^−2^, with the lowest directly measured dose rate of 72 nGy_air_ s^−1^.^[^ ^249^ ^]^ Peng et al. employed a similar strategy to achieve high‐performance X‐ray detectors from polycrystalline composite films.^[^ ^273^ ^]^ It is difficult to achieve large area detectors based on perovskite single crystals and integrate them with TFT arrays. On the other hand, polycrystalline films suffer from relatively poor charge collection efficiency due to non‐radiative recombination at grain boundaries. Peng et al. addressed these problems by loading crushed CH_3_NH_3_PbI_3_ single crystals into an organic polymethyl‐methacrylate (PMMA) matrix to fabricate polycrystalline composite films. This method achieved polycrystalline films with large grain size of up to a few microns and pinholes between these large grains were filled out by the PMMA matrix, avoiding short‐circuiting and minimizing leakage current, resulting in a significantly improved sensitivity for X‐rays compared to other CH_3_NH_3_PbI_3_ polycrystalline film‐based detectors.^[^ ^28^, ^273^ ^]^ Also Zaffalon et al. recently demonstrated polymeric nanocomposites of CsPbBr_3_ NCs embedded into PMMA matrix, which exhibited remarkable stability even after exposure to an extreme γ‐ray dose of 1 MGy, showing that composites are an excellent route to enhance radiation hardness of halide perovskite NCs and QDs.^[^ ^40^ ^]^

Perovskite‐based thick (greater than few tens of microns) film flexible detectors are difficult to achieve, as increasing thickness compromises the ability to bend them while avoiding fracture. Zhao et al. demonstrated an excellent way to achieve thick (>1 mm) devices for flexible large area radiation detection via composites.^[^ ^272^ ^]^ In their work, they uniformly infiltrated saturated MAPbI_3_ solution into porous nylon fibers (≈100 µm thick) and annealed them. These perovskite‐filled nylon sheets were laminated to achieve the desired thickness. With a device of thickness ≈1 mm, 100% attenuation efficiency was achieved for 60 keV X‐rays, which resulted in a remarkable sensitivity of 8700±200 µC Gy_air_^−1^ cm^−2^ under a field of 500 V cm^−1^. Highly flexible devices with bending curvatures as low as 2 mm were achieved for 130 µm thick devices, which enable them to image even narrow hollow objects (like pipes) from inside.^[^ ^272^ ^]^ This type of imaging of hollow objects from their interior to image only region of interest, avoids unwanted X‐ray attenuation due to non‐targeted parts and helps in achieving high contrast images at lower X‐ray energies, when compared to what is required for flat panel imaging.^[^ ^272^ ^]^ The strategy of perovskite‐filled membranes employed here allows facile tunability of attenuation efficiency and flexibility by tuning device thickness for application‐specific requirements. Similar approaches to using perovskite composites could also be employed for the direct detection of γ‐rays, but this has not yet been explored.

### 4.6 Heterostructures, Heterojunctions, and Perovskite/Electrode Interfaces

**Figure 17:**
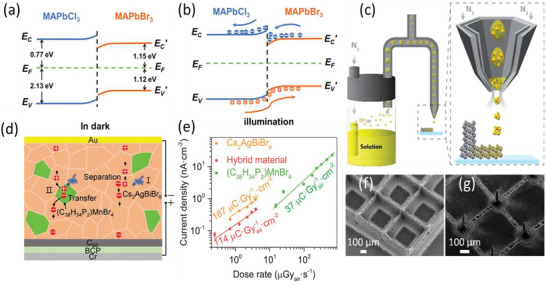
Band diagrams of MAPbCl_3_‐MAPbBr_3_ single crystal heterojunctions a) in the dark and b) under illumination. Reproduced with permission.^[^ ^274^ ^]^ Copyright 2023, Wiley‐VCH GmbH. c) Schematic of an aerosol jet printing system. d) Illustration of charge generation, transfer, and separation mechanisms in a hybrid perovskite radiation detector. e) X‐ray detection performance of a hybrid material wafer, Cs_2_AgBiBr_6_ and (C_38_H_34_P_2_)MnBr_4_ wafers. Parts (d) and (e) reproduced with permission.^[^ ^275^ ^]^ Copyright 2021, Wiley‐VCH GmbH. f,g) 3D printed architectures of perovskites on glass substrate using aerosol jet printing. Parts (c), (f), and (g) reproduced with permission.^[^ ^276^ ^]^ Copyright 2021, American Chemical Society.

Heterostructures, where two or more perovskite materials are grown in a heterojunction, can lead to improved charge‐carrier transport and stable device performance. Perovskite‐based heterostructures are widely used in photodetectors operating in the UV‐Vis‐NIR range and have recently been extended to X‐ray detection as well.^[^ ^277^, ^278^ ^]^ In direct radiation detectors, large dark currents compromise the SNR, limiting their device performance. Heterostructures combine semiconductors of different bandgaps to create a built‐in field at the heterojunction, which is responsible for efficient charge‐carrier separation, improved charge‐carrier transport, and low dark current. Typically, in heterostructures, the active layer material is sandwiched in between electron and hole‐blocking layers, which facilitate charge separation and block current injection from the electrodes into the active layer under reverse bias, thus lowering the dark current.^[^ ^69^ ^]^ In addition, the built‐in electric field at heterojunctions can separate electron‐hole pairs created through ionization without requiring any external bias to be applied (similar to solar cells operating under short‐circuit). This therefore enables radiation detectors to operate in self‐powered mode (**Figure** **17**a,b). However, heterostructures that suffer from large lattice mismatch at the heterojunctions can lead to poor interface quality, which can result in ion migration, interfacial non‐radiative recombination, and ion accumulation. Hence lattice mismatch must be minimized for robust, high‐sensitivity radiation detection.

Epitaxially grown films minimize lattice mismatch and are advantageous over traditional spin‐coated films, as they reduce interface defects. In epitaxially‐grown films, the thickness can be controlled easily by adjusting the growth time, and they can be grown into comparatively thicker films, which is required for better attenuation of high‐energy radiation. Xu et al. have grown epitaxial layers of MAPbBr_3_ and MAPbI_3_ onto MAPbBr_2.5_Cl_0.5_, where MAPbBr_3_ acts as a buffer layer to compensate for lattice mismatch between MAPbI_3_ and MAPbBr_2.5_Cl_0.5_, and achieved less than 5% lattice mismatch.^[^ ^279^ ^]^ Their *p*‐*i*‐*n* type heterostructure with device structure Au/MAPbI_3_/MAPbBr_3_/MAPbBr_2.5_Cl_0.5_/C_60_/PCBM/Ag, has shown stable dark current under an electric field of 959.7 V cm^−1^ and a low dark current density of 0.697 pA mm^−2^ at ‐0.5 V applied bias. Owing to such low dark current densities, which is 3 orders lower than the dark current density of devices without heteroepitaxial layers, this photodetector obtained a high sensitivity of 59.7 × 10^3^ µC Gy_air_^−1^ cm^−2^ for X‐rays at a dose rate of 6.7 µGy_air_ s^−1^. Epitaxially‐grown MAPbBr_3_‐CsPbBr_3_ single crystal heterojunctions based on inverse‐temperature crystallization was investigated by Cui et al. and this device exhibited a sensitivity of 2.0 × 10^5^ µC Gy_air_^−1^ cm^−2^, with an LoDD of 96 nGy_air_ s^−1^ for 120 keV hard X‐ray detection when operated at ‐1250 V cm^−1^.^[^ ^280^ ^]^ Such superior device performance is a result of rectifying the diode behavior of this heterojunction due to the presence of *p*‐type MAPbBr_3_ and *n*‐type CsPbBr_3_, which highly suppressed the detrimental dark current, and dark current drift caused by ion migration, under reverse bias conditions.

2D–3D heterostructures benefit from both 2D structures (high resistivity and reduced ion migration) and 3D structures (higher charge‐carrier transport). He et al. found that 2D‐3D heterocyrstals suppressed the ion migration that dominates in 3D crystals.^[^ ^281^ ^]^ In heterostructures, optimizing the thickness of functional layers is crucial, as it controls the *µτ*‐product and length of the depletion region across the junction. In this work, the thickness of the 2D 4‐fluorophenethylammonium lead bromide (FPEA_2_PbBr_4_) perovskite that was epitaxially grown onto 3D FAPbBr_3_ single crystal was optimized to achieve the best *µτ*‐product. A *µτ*‐product of 2.42 × 10^−3^ cm^2^ V^−1^ was obtained for a heterojunction device with 13 µm thick 2D layer, which is 2 times higher when compared to that of 19 µm thick 2D layer heterojunction. Their best‐performing heterostructure device achieved a LoDD of 55 nGy_air_ s^−1^ for 120 kV_p_ hard X‐rays, which is ≈5 times lower than the 3D FAPbBr_3_‐only device, thus demonstrating the potential of heterostructures for effective low‐dose radiation detection.^[^ ^281^ ^]^

As mentioned earlier, heterostructuring is essential for self‐powered radiation detection to eliminate the need for subjecting devices to high external bias voltages that can trigger ion migration and device degradation. Self‐powered devices are useful for portable radiation detection applications, e.g., personal dosimeters. Yan et al., fabricated a self‐powered detector using an epitaxially grown MAPbBr_3_‐MAPbCl_3_ heterocrystal with a low lattice mismatch of only 3.3%, and achieved an LoDD of 70 nGy_air_ s^−1^ (directly measured), and 15.5 nGy_air_ s^−1^ via extrapolation.^[^ ^274^ ^]^ The device also showed a high sensitivity of 868 µC Gy_air_^−1^ cm^−2^, without any external bias, for 50 kV X‐rays. Other groups also reported excellent passive radiation detection performance of heterostructured devices, indicating a great potential for heterostructures.^[^ ^282^, ^283^ ^]^ In a pioneering work on lead‐free perovskite heterostructures, Zhang et al. grew an epitaxial heterocrystal based on 2D halide double perovskites (BA)_2_CsAgBiBr_7_ and 3D perovskite Cs_2_AgBiBr_6_ with a nearly atomically‐sharp interface, which led to a built‐in potential with a photovoltage of ≈0.2 V under X‐ray illumination at a dose rate of 40 µGy_air_ s^−1^ and enabled self‐powered operation of these 2D‐3D heterocrystals.^[^ ^284^ ^]^ The sensitivity achieved in these heterostructures without any external bias applied (201 µC Gy_air_^−1^ cm^−2^) was still an order of magnitude higher than that of the 3D crystal alone or the 2D crystal alone under external bias, demonstrating the potential of heterostructures for superior radiation detection.^[^ ^284^ ^]^ The planar device architecture employed here helps to minimize the loss of photo‐excited charge‐carriers in trap states in the bulk of the crystals, and exposes the heterojunction to incoming radiation to maximize the photovoltaic effect.

For effective radiation detection, perovskite–electrode interface quality is also crucial. However, the high polarity and chemical reactivity of halide perovskites, arising from their ionic nature, can lead to complex interfacial effects, such as ion accumulation and electrochemical reactions, which result in the formation of interfacial defect states and may cause Fermi‐level pinning.^[^ ^285^ ^]^ External stimuli, such as electrical biasing and exposure to radiation, can promote redox reactions at perovskite‐electrode interfaces, oxidizing the metal electrode and leading to the formation of metal halides and halide vacancies.^[^ ^286^, ^287^ ^]^ The presence of such impurities and defects can escalate charge‐carrier scattering and non‐radiative recombination at perovskite‐electrode interfaces, thereby compromising on the charge collection efficiency in detectors.^[^ ^285^ ^]^ Introducing a very thin (few nm thick) chemically inert interlayer, such as phenyl‐C61‐butyric acid methyl ester (PCBM) or polymethylmethacrylate (PMMA), between the perovskite and electrode has proven to mitigate such electrochemical reactions and improve device performance.^[^ ^288^, ^289^ ^]^ Moreover, the soft lattice structure of halide perovskites can also facilitate metal ion diffusion from the electrodes into the bulk of the perovskite, leading to the formation of shallow or deep traps, depending on the type of metal ion.^[^ ^290^ ^]^ Also, common electrode deposition techniques, such as physical vapor deposition (PVD) involve bombarding the perovskite surface with high‐energy metal atoms, which can cause impact‐induced defects due to the perovskite's low Young's modulus, which is at least an order of magnitude lower than conventional inorganic semiconductors like Si and GaN.^[^ ^291^, ^292^, ^293^ ^]^ Lee et al. demonstrated from SCLC measurements that electrode deposition using PVD increased the defect density of MAPbI_3_ films by 26%‐48%.^[^ ^291^ ^]^ To avoid this issue, alternative electrode deposition routes, such as stamping transfer and physical lamination of pre‐deposited electrode films, have been explored.^[^ ^291^, ^294^, ^295^ ^]^

Using asymmetric electrodes helps to achieve low dark currents even under a high electric field. He et al. employed eutectic Ga‐In alloy and Au as electrodes in CsPbBr_3_ γ‐ray detectors and achieved a low dark current of 780 pA mm^−2^ under an electric field of 2000 V cm^−1^.^[^ ^55^ ^]^ Furthermore, to suppress the influence of charge‐carrier trapping through unipolar sensing mode, contact geometries like quasi‐hemispherical and pixelated configurations were explored, which achieved remarkable energy resolutions of 1.8% and 1.6%, respectively for 662 keV γ‐rays.^[^ ^55^ ^]^ Beyond the judicious choice of the electrode and charge transport layer materials to minimize dark currents, surface, and edge leakage currents have also been suppressed by using a guard ring electrode.^[^ ^100^ ^]^ This involves having a metal electrode surrounding the circular central electrode, separated by a small channel of tens of microns. By having the same applied field between the guard electrode and anode as the central electrode and anode, the surface and edge leakage currents are taken up by the guard electrode. Wei et al. demonstrated this strategy with CsPbBr_2.94_Cl_0.06_ single crystal detectors, achieving a four‐fold reduction in dark current through the use of a guard electrode.^[^ ^100^ ^]^

### 4.7 Hybrid and Integrated Devices

Halide‐perovskite‐based indirect radiation detectors or scintillators suffer from scintillated light scattering, self‐absorption, and after‐glow, which compromise their spatial and time resolution.^[^ ^296^, ^297^ ^]^ On the other hand, although direct detectors, in most cases, have a better spatial resolution, the applied biases required to achieve sufficiently high charge‐collection efficiency could trigger detrimental ion migration, which can cause baseline dark current drift and increase the LoDD.^[^ ^298^ ^]^ Hybrid radiation detectors make use of both scintillation and direct detection mechanisms to effectively mitigate these drawbacks, and can give rise to superior detector performance.^[^ ^275^, ^297^, ^299^, ^300^ ^]^

Traditionally, scintillators are integrated onto silicon photodiodes for converting the downconverted light emission into an electrical signal. Replacing these silicon photodiodes with perovskite photodiodes can enhance the overall performance, due to its ability to function simultaneously as a photodetector for both UV, visible, and high‐energy radiation through direct detection.^[^ ^299^, ^300^ ^]^ Li et al. employed this strategy and designed an all‐perovskite integrated radiation detector using CsPbBr_3_ NCs as the scintillating medium and MAPbI_3_ for the photodiode.^[^ ^300^ ^]^ As opposed to the weak responsivity of Si photodiodes (typically <0.2 A W^−1^), the MAPbI_3_ photodiodes designed here with a device structure of Ag/PCBM/MAPbI_3_/NiO/FTO, exhibited much higher responsivity of up to 0.45 A W^−1^ for visible light and greater than 0.35 A W^−1^ for X‐rays. As a result, integrating CsPbBr_3_ NC film with this perovskite photodiode produced an effective radiation detector with a high sensitivity of 54 684 µC Gy_air_^−1^ cm^−2^ for 22 keV X‐rays at a dose rate of 8.8 µGy_air_ s^−1^.^[^ ^300^ ^]^

Another method is to combine both scintillating materials and direct detectors in the active medium for hybrid radiation detectors. Li et al. used low‐cost fast tableting method (pressing to form tablets) to make hybrid devices from mixed powders of Cs_2_AgBiBr_6_ semiconductor and (C_38_H_34_P_2_)MnBr_4_ scintillator.^[^ ^275^ ^]^ Here, (C_38_H_34_P_2_)MnBr_4_ forms a type II energy band alignment with Cs_2_AgBiBr_6_ at interfaces, leading to the transfer of photogenerated charge‐carriers from the perovskite sensitizer to the halide elpasolite semiconductor (Figure 17d). Avoiding photoluminescence from the scintillator eliminates problems of self‐absorption and afterglow. A very similar approach was adopted by Liu et al. from the same research group to study hybrid devices based on MAPbI_3_ semiconductor and Cs_3_Cu_2_I_5_ scintillator.^[^ ^297^ ^]^ In both cases, owing to this fast charge transfer mechanism, the timing response was significantly improved from microseconds (scintillator devices, limit by afterglow), to just a few nanoseconds in hybrid devices.^[^ ^275^, ^297^ ^]^ Moreover, dark current drift was remarkably decreased by 3 orders of magnitude, due to a reduction in ion migration in comparison with pure semiconductor detectors, which resulted in improved detection limit (Figure 17e).^[^ ^275^, ^297^ ^]^ Liu et al. also demonstrated imaging capability of these hybrid devices, which showed a spatial resolution of 0.5 lp mm^−1^ with a pixel size of 1 mm × 1 mm.^[^ ^297^ ^]^ This work illustrates the potential of hybrid radiation detectors which could also be employed for γ‐ray detection in the future.

### 4.8 Other Strategies

A handful of researchers have reported the enhancement of optical, electrical, and structural properties in halide perovskites after low dose γ‐ray exposure (<200 kGy_air_, although the dose rate employed has varied greatly between groups),^[^ ^301^, ^302^, ^303^, ^304^ ^]^ due to defect passivation, ion redistribution and lattice reparation (self‐healing; see Figure 8c‐e). However, after a threshold accumulated γ‐ray dose, structural decomposition occurs, leading to performance degradation.^[^ ^301^, ^303^, ^304^ ^]^ Xu et al. demonstrated a MAPbBr_3_ single crystal‐based γ‐ray detector, where the samples exposed to 5.52 kGy_air_ γ‐rays showed about 5‐times increase in photocurrent as compared to as‐grown unexposed samples, but clear underlying mechanism for such enhancement was not explained.^[^ ^301^ ^]^ This approach of low dose γ‐rays exposure for enhancing the properties of perovskites is still controversial, and elaborate studies are required to further understand the synergistic effects of defect formation and self‐healing mechanisms under γ‐ray irradiation.^[^ ^304^ ^]^

In addition to improving the intrinsic material properties of halide perovskites, it is also essential to design practical and scalable approaches for their integration onto large thin film transistor (TFT) substrates for high‐resolution imaging applications.^[^ ^81^, ^276^, ^305^ ^]^ Perovskite aerosols surpass perovskite solutions in achieving large‐area thick films with fewer grain boundaries, pinholes, and cracks via controllable crystallization. Quian et al. grew CsPbI_2_Br thick films (>200 µm thick) directly onto 100 cm^2^ large TFT substrates using precursor aerosols, where these devices exhibited a high sensitivity of 1.48 × 10^5^ µC Gy_air_^−1^ cm^−2^, along with an LoDD of 280 nGy_air_ s^−1^.^[^ ^305^ ^]^ Furthermore, for printing technologies, although well‐established routes exist for perovskite solution‐based inks, solution spatter and dissolution of already existing layers still remain challenges to be tackled. Glushkova et al. demonstrated a novel aerosol‐based jet‐printing method that is capable of printing high aspect ratio 3D architectures with µm‐level precision (Figure 17f,g).^[^ ^276^ ^]^ Here, halide perovskite crystals are stabilized as soluble complexes in a polar aprotic solvent which are vented out through nozzle as aerosols using nitrogen flux (Figure 17c), which enables solvent volatilization of the droplets prior to their arrival onto substate minimizing spatter and dissolution of previous layers.^[^ ^276^ ^]^ Such promising routes to grow micron‐sized structures onto pixelated arrays hold great potential for further improving the spatial resolution of direct radiation detectors.

## 5 Challenges for Commercialization

Whilst lead‐halide perovskites can overcome many of the shortcomings of classical semiconductor‐based radiation detectors, challenges still remain before they can be commercialized. The main challenge is the operational stability of the perovskite detectors because the device's degradation can be triggered by environmental factors (i.e., moisture and O_2_ in air), operational conditions (i.e., high applied biases, heat, and illumination), or exposure to high photon fluxes.^[^ ^306^ ^]^ Scaling up the detectors from small single crystals, and developing approaches to manufacture cost‐effectively with high throughput, are also important challenges. In this section, we discuss these challenges in detail, covering the effects of environmental factors on device stability (Section 5.1), encapsulation materials to protect the detectors from these environmental factors (Section 5.2), suppressing field‐induced ion migration (Section 5.3), and detector uniformity and scale‐up of both single crystal wafers and thick films (Section 5.4).

### 5.1 Device Instability from Environmental Factors

Whilst investigations into the environmental stability of perovskite X‐ray detectors are largely lacking in the field, we can make use of the extensive studies made into the operational stability of perovskite photovoltaics and photodetectors. The environmental instability of perovskite devices is primarily attributed to the degradation of perovskite materials caused by heat, oxygen, and moisture, particularly under illumination. High temperatures accelerate ion migration and lead to the removal of organic cations, which induces the degradation of the material structure.^[^ ^307^ ^]^ Under illumination in air, oxygen can be incorporated into the lattice. Excessive oxygen incorporation can cause the rapid decomposition of the perovskite materials.^[^ ^308^ ^]^

Very recently, a work by Tsai et al. discovered that humidity in the environment is especially problematic.^[^ ^309^ ^]^ The presence of moisture promotes ion migration when operating the detector under low bias. Operating the detectors at high bias in high humidity (40% relevant humidity) led to complete failure within a few minutes. This humidity‐induced degradation is not a surprise because similar phenomena are found in perovskite solar cell lifetime tests.^[^ ^310^ ^]^ Water molecules can penetrate the top electrode and arrive at defect sites in the perovskite materials, promoting the movement of mobile ions. An effective protection scheme was proposed by Tsai et al. They found that growing a hydrophobic capping layer on the perovskite's surface can efficiently mitigate the penetration of water molecules. As a result, the detectors can operate more reliably under high relative humidity levels (40%), and the breakdown threshold voltage is increased from 6 kV cm^−1^ to 10 kV cm^−1^.^[^ ^309^ ^]^

### 5.2 Encapsulation

**Figure 18:**
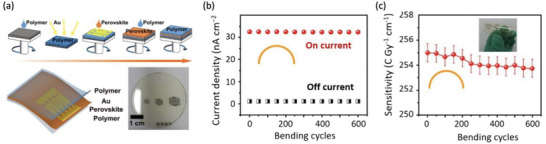
a) Schematic illustrating the fabrication of the flexible polymer‐encapsulated Cs_4_PbI_6_ detectors. b) On–off current control and c) the sensitivity of the perovskite detector device after 600 bending cycles with a bend angle of 90°. Figures adapted with permission.^[^ ^314^ ^]^ Copyright 2020, American Chemical Society.

A practical strategy to address the challenges associated with low LHP environmental stability is through encapsulation. Although encapsulation has been extensively investigated for perovskite photovoltaics, limited efforts on protecting perovskite radiation detectors have been made thus far due to their harsh working conditions.^[^ ^311^ ^]^ Generally, the encapsulation materials for radiation detectors should fulfill the following requirements: 1) be cost‐effective and transparent to ionizing radiation, 2) block UV/visible light to ensure the photocurrent is only generated through X‐rays/γ‐rays, 3) have excellent thermal, moisture and oxygen resistance for the detectors to operate under ambient or harsh environments, 4) have high adhesion to avoid mechanical damage to the device, 5) have high stability under X‐ray illumination, and 6) avoid radiation scattering to minimize background heterogeneity and therefore improve image contrast and sharpness.^[^ ^140^ ^]^ In this regard, cost‐effective materials like thick glass (which are commonly used in optoelectronic devices) cannot be used to encapsulate radiation detectors because of their strong absorption of X‐ray radiation. Polymers, including poly(vinyl alcohol‐*co*‐polymer) (PVA), Surlyn, polyethylene terephthalate (PET), polytetrafluoroethylene (PTFE), polycarbonate (PC), poly(*p*‐chloro‐xylene) (parylene‐c), and poly(ethylene vinyl alcohol) (EVOH) are good candidates for packaging perovskite radiation detectors because these are low‐*Z* materials.^[^ ^312^ ^]^ The fabrication of perovskite/polymer composite films has been shown to not only improve stability but also allow for great flexibility due to the contribution of the polymer matrix (see Section 4.5 for a detailed discussion).^[^ ^313^ ^]^ For example, X‐ray detectors made of perovskite/nylon composites can maintain their functionality even when bent to a radius as small as 2 mm, and the devices showed no degradation after storage for over six months, or after exposure to a dose of 376.8 Gy_air_, equivalent to 1.88 million chest X‐ray scans.^[^ ^272^ ^]^ Polymer‐encapsulated Cs_4_PbI_6_ vacancy‐ordered perovskite reported by Chen et al. were found to retain the same performance after being stored in the air for 60 days, or after bending for 600 cycles (**Figure** **18**a–c).^[^ ^314^ ^]^ Metal foil can also be used to encapsulate X‐ray detectors to retain environmental stability.^[^ ^105^ ^]^ However, it is currently still quite challenging to obtain perovskite radiation detectors with both high stability and excellent optoelectronic performance.

Another strategy to encapsulate radiation detectors is to store them under inert gas or vacuum, with a low‐*Z* Be window. This strategy was adopted by Zhang et al. for Cs_2_AgBiBr_6_ radiation detectors, which were mounted in a N_2_‐filled stainless steel chamber with a 100 µm thick Be window. The Be window blocks ambient light, and yet has 99.7% transmittance for X‐rays (30 keV energy and above).^[^ ^140^ ^]^ These encapsulated devices exhibited excellent sensitivity up to 1 × 10^4^ µC Gy_air_^−1^ cm^−2^ with a low detection limit of 145.2 nGy_air_ s^−1^. This high performance was maintained even after 2 months of storage. When such gas‐filled encapsulating chambers are designed, the contribution of gas ionization to the photocurrent signal must also be considered.^[^ ^51^ ^]^ Encapsulating under vacuum could be an ideal alternative to eliminate air/gas ionization effects.^[^ ^125^ ^]^

### 5.3 Suppressing Field‐Induced Ion Migration for γ‐ray Spectroscopy and X‐ray Imaging

**Figure 19:**
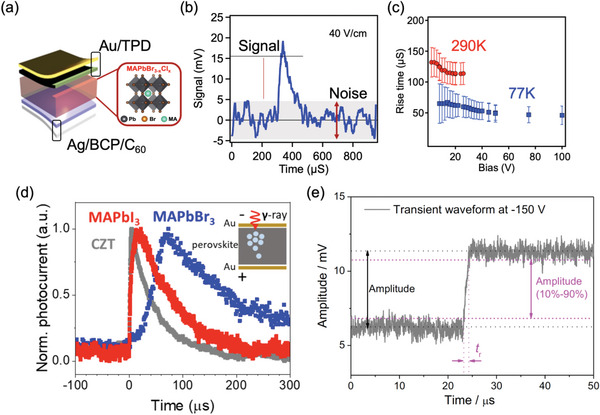
a) Schematic of a *p*‐*i*‐*n* structured MAPbBr_3−_ *_x_* Cl*_x_* perovskite device. b) Typical pulse from a MAPbBr_3_ single crystal detector when operated under a field of 40 V cm^−1^. c) Rise time of the detector as a function of applied bias tested at room temperature (red) and 77 K (blue), (the radiation source here is ^137^Cs γ‐rays). d) Typical pulse signals collected from a MAPbI_3_ detector, MAPbBr_3_ detector and a CZT single crystal detector. e) Pulse from a Bridgman‐grown CsPbBr_3_ single crystal detector. Part (a)–(c) are adapted with permission.^[^ ^317^ ^]^ Copyright 2020, Elsevier. Part (d) adapted with permission.^[^ ^318^ ^]^ Copyright 2020, American Chemical Society. Part (e) is reproduced under the terms of the CC‐BY license.^[^ ^45^ ^]^ Copyright 2019, The Authors. Published by Springer Nature.

An effective radiation detector, especially for hard X‐rays and γ‐rays, is typically made with a large volume of material with thicknesses on the mm scale or larger. Achieving high charge‐collection efficiencies would then typically require an external electric field to be applied. As discussed earlier in Section 3.2, applying an electric field can also be important for decoupling charge carriers from the renormalization of the lattice to enable long drift lifetimes, and therefore larger Schubwegs.^[^ ^54^ ^]^ For γ‐ray spectroscopy, applying a large field is particularly important to extract the small number of ionized charge carriers created from the arrival of each γ‐ray photon.^[^ ^315^ ^]^ A typical LHP γ‐ray detector is shown in **Figure** **19**a, and the typical pulse obtained following interaction with a γ‐ray photon is shown in Figure 19b.^[^ ^275^ ^]^ To accurately determine the pulse height, the detector's signal should be well above the dark current baseline. The rise and fall time of the pulses should also be as short as possible to avoid pulse pile‐up and other problems.^[^ ^316^ ^]^ Relatively high electric fields are required to achieve a distinct shape and signal of the pulse, along with fast rise/fall in signal. For example, Liu et al. showed that the rise/fall time of MAPbBr_3_ detectors decreased as the applied bias increased (Figure 19c).^[^ ^316^ ^]^ At the same time, applying higher fields can increase the fluctuation of the dark current. It is therefore important to develop a detector that can withstand high applied biases, but with low dark current drift.^[^ ^316^ ^]^

It is therefore important to minimize defect densities in both the bulk of the active layer, as well as at interfaces. For example, Shrestha et al. found that MAPbI_3_ single crystal photoconductor detectors exhibited a faster response to γ‐ray photons with a better pulse shape when Pb/MAPbI_3_ Schottky contacts were used, rather than Au/MAPbI_3_ Ohmic electrodes, by reducing the dark current.^[^ ^318^ ^]^ The resulting detectors had a much‐improved pulse shape, with a rise time as short as a few microseconds at high applied biases (Figure 19d), reaching the performance of CZT detectors (Figure 19d).^[^ ^318^ ^]^ As another example, Bridgman‐grown CsPbBr_3_ crystals presented by the Kanatzidis group exhibit remarkably short pulse rise times of less than 1 µs when a high electric field was applied (Figure 19e). Achieving this result required the crystals to have low defect densities and low levels of impurities, up to only 10 ppm for 69 elements.^[^ ^45^, ^55^ ^]^

However, as mentioned earlier in Sections 3.2 and 4, LHPs are mixed ionic‐electronic conductors, and applying high electric fields can trigger ion migration, leading to the detector becoming unreliable. Ion migration has been proposed as the likely underlying cause of the current–voltage hysteresis observed in lead‐halide perovskite devices. Hysteresis is manifest as a gradual current response when the device experiences rapid changes in irradiance and/or changes in the scan direction, scan rate, voltage range, and pre‐poling voltage.^[^ ^319^ ^]^ For example, in perovskite solar cells, hysteresis is manifest in the current–voltage sweeps in the forward and reverse directions being different, often substantially different.^[^ ^320^, ^321^ ^]^ But unlike photovoltaic device characterization, X‐ray detectors are typically measured under a constant field applied for a specified period of time. At the same time, current–voltage sweeps in the dark are still used to measure the resistivity of X‐ray detectors, and hysteresis would then limit how accurately such physical properties can be measured.^[^ ^322^ ^]^ Furthermore, ion migration can result in a change in the baseline dark current, as well as the photocurrent, over time under a constant applied field. This occurs because mobile ions can migrate to the interfaces of the device, screening the electric field within the device, or affecting charge‐injection/extraction at the electrodes. With a constantly drifting baseline, it is challenging to determine the photo‐generated signal accurately, which affects the accuracy of the sensitivity values obtained. Moreover, ion migration could decrease charge‐carrier mobilities, thereby impacting the photoresponse characteristics (such as rise and fall times) of the detectors. This highlights the importance of understanding and mitigating hysteresis effects in lead‐halide perovskite devices to enhance their performance and reliability. Previous experimental and theoretical studies have demonstrated that halide ions (Cl, Br, and I) are the main mobile species that migrate in LHPs because of the low formation energies of halide vacancies, which facilitate halide ion migration.^[^ ^209^ ^]^ The migration of ions can result in phase segregation, void formation, or other defects in the film, which can trap charge‐carriers and impair the optoelectronic properties of the perovskite materials.^[^ ^322^ ^]^ Theoretical studies have revealed that alloying the halides (i.e., Cl mixed with Br) can mitigate ion migration, yielding a more robust γ‐ray detector.^[^ ^266^ ^]^ Halide alloying is also proven to be beneficial to balance electron‐hole mobilities.^[^ ^100^ ^]^ Additive engineering of perovskite materials has emerged as an effective approach for mitigating ion migration or hysteresis in perovskite devices.^[^ ^319^ ^]^ Notably, the addition of a foreign fullerene derivative (PCBM) to the perovskite precursor can suppress hysteretic behavior while doubling the mobility of carrier transport at room temperature compared to perovskite devices without any additives.^[^ ^323^, ^324^ ^]^ PCBM can minimize anion migration through defects at grain boundaries by binding to iodide‐rich surface sites, or simply unincorporated iodide anions. This holds great promise for improving the performance and reliability of perovskite detectors.^[^ ^324^ ^]^

Perovskite X‐ray detectors operating in current mode also suffer from baseline drift and corrosion of the metal electrode due to ion migration under an applied field.^[^ ^119^ ^]^ A general agreement in the literature is that device degradation induced by ion migration is more severe when the device is operated under high external bias and/or light illumination.^[^ ^325^ ^]^ In addition, the current will drift more severely, which greatly limits the use of perovskite devices in radiation detector applications. As a consequence, the highest electrical field applicable over the perovskite detectors is limited. To avoid material degradation caused by the applied electric field, the detectors can be operated under a lower bias, but this would then limit the charge‐collection efficiencies achievable.

These polarization effects can be suppressed through the use of low‐dimensional perovskites, as discussed in Section 3.2, as well as Section 4.1.1.^[^ ^326^ ^]^ However, it is important to develop methods to grow large‐sized 2D or 0D crystals with high quality. A typical 2D perovskite single crystal formed with weak *van der Waals* interactions between layers can potentially be exfoliated into small flakes. cm‐sized 2D perovskite single crystals have been demonstrated, but only with *n* = 1 materials so far, which have poor out‐of‐plane conductivity.^[^ ^322^, ^327^ ^]^ Alternatively, DJ 2D perovskites can be mechanically more robust because the layers are held together with double charged linkers via electrostatic interactions. 0D perovskites are also promising alternatives to stabilize the performances. However, 0D perovskites are usually demonstrated for indirect, scintillator detectors, because charges are strongly localized in the 0D structure, such that rapid radiative recombination occurs.^[^ ^328^ ^]^ Driving ionized charges through the bulk over a distance of a few mm to cm can be challenging. Adding dopants to increase the density of states, or replacing insulating linkers with semiconducting conjugated organics may help to improve the conductivity.^[^ ^329^ ^]^

On the device level, Tisdale et al. found that polarization due to interface defects can be mitigated by switching the applied field's direction.^[^ ^316^ ^]^ The authors applied an electric field for a short duration of time and switched the field's polarity. As a result, the polarized interface was “de‐polarized” once switching the electric field, allowing the detector to “recover” its interface. This switching can maintain the dark current and photocurrent of the detector for an extended period of time. This method was developed for photoconductor devices that have the same signal amplitude with opposite signs at opposing applied biases. Along the same lines, Jin et al. developed a perovskite/indium oxide photo‐transistor device. In this case, the perovskite functions as a photo‐sensitizer, which generates charges and injects them into the metal oxide conducting channel. The charges will be collected across the indium oxide channel under bias, thus avoiding problems associated with ion migration and accumulation. Similar concepts have also been demonstrated by interfacing perovskite with other transistors, such as InGaZnO,^[^ ^330^ ^]^ conducting polymers,^[^ ^92^ ^]^ and graphene.^[^ ^276^ ^]^

Apart from materials innovations, such as using low‐dimensional structures (see Section 4.2), device geometry optimization could be another strategy to suppress field‐induced ion migration. For instance, integrating perovskites into porous matrices can stabilize the material and reduce the channels available for ion migration. As an example, Zhu et al. grew cesium lead halide perovskites into a porous anodic aluminium oxide matrix, and successfully demonstrated a reduction in ion migration and inter‐pixel cross‐talk.^[^ ^331^ ^]^ Along a similar line, other porous matrices such as metal–organic frameworks and polyethylene terephthalate have been employed to suppress ion migration in detectors.^[^ ^332^, ^333^, ^334^ ^]^ In addition, porous polymer matrices have also been shown to be effective in improving the polarization stability of perovskite X‐ray detectors. Zhao et al. showed that by filling methylammonium lead iodide (or iodide‐chloride) perovskites into porous nylon membranes, the stability was improved such that the X‐ray induced current showed no reduction after continuous operation for 5131 min at 12 V under 60 keV X‐rays, and a high dose rate of 1224 µGy_air_ s^−1^.^[^ ^272^ ^]^ Cs_2_AgBiBr_6_ elpasolites have been also been integrated into polymers, with a modest sensitivity of 40 µC Gy_air_ s^−1^, and stable cycling under X‐rays with a dose rate of 13.8 mGy_air_ s^−1^ for at least 50 s.^[^ ^313^ ^]^ In other types of perovskite devices, epitaxial growth and strain engineering have emerged as highly effective methods to stabilize the interfaces used, and these approaches should be utilized for perovskite X‐ray detectors.^[^ ^335^, ^336^ ^]^

As ion migration is usually facilitated by defects and imperfections near the surface, immobilizing interface defects could be another strategy to suppress ion migration. For example, building a lattice‐matched interface via epitaxial growth was employed to suppress the ion migration from the perovskite layer. Liu et al. have attempted such a growth by interfacing MAPbX_3_ with a BaWO_4_ matrix, resulting in an effective inhibition of halide migration.^[^ ^337^ ^]^ The restriction of halide migration is attributed to the BaWO_4_ matrix's lattice‐matched epitaxial growth and the enhanced rigidity that stabilizes the interface.^[^ ^337^ ^]^

It is also postulated that the implementation of self‐powered photodetectors could mitigate perovskite degradation from an applied external electric field.^[^ ^338^ ^]^ Advances in device architecture have shown promising results in terms of providing a consistent power supply. Further investigations are deemed necessary to validate these findings and explore other potential avenues for maintaining long‐term performance.^[^ ^51^, ^105^, ^125^, ^140^, ^272^, ^311^, ^312^, ^313^, ^314^ ^]^

### 5.4 Uniformity and Scaling‐Up Production

As discussed in Sections 3 and 4, a wide variety of perovskite compositions and structures have been exploited for high‐performance radiation detection. However, the upscaling of solution‐grown single‐crystal perovskites is still the most urgent technological bottleneck due to the poor solubility of the precursors, and difficulties in controlling the phases obtained in the crystals grown.^[^ ^339^ ^]^ Very recently, a solution‐based lithography‐assisted epitaxial‐growth‐and‐transfer method was introduced to prepare large‐scale (5.5 cm × 5.5 cm) perovskite single crystals.^[^ ^340^ ^]^ The arrays of tiny perovskite single crystals were designed to facilitate simultaneous epitaxial crystal growth, which subsequently led to the merging of neighboring crystals into a larger, single‐crystalline perovskite over a larger area. The implementation of this method is expected to create a new opportunity for the fabrication of larger‐scale perovskite single crystals with different compositions.

**Figure 20:**
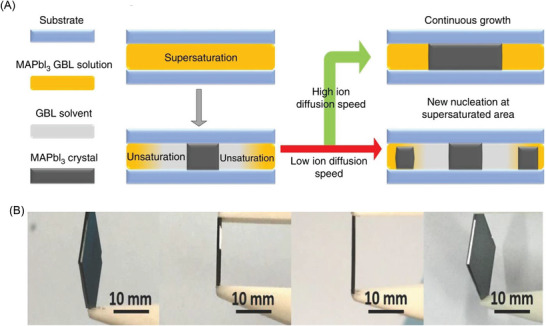
Single crystal wafer growth. a) Illustration of the “confined space” growth method. Reproduced under the terms of the CC‐BY license.^[^ ^342^ ^]^ Copyright 2017, The Authors. Published by Springer Nature. b) Large‐scale single crystal wafers. Image reproduced with permission.^[^ ^341^ ^]^ Copyright 2016, Wiley.

On the other hand, the time‐consuming preparation of large‐scale single crystals, along with their high production costs, and high rigidity still limits the application of perovskite single crystals for flexible electronic devices and further commercialization.^[^ ^272^ ^]^ Making perovskite wafers presents a viable path for scaling up radiation detectors. Single crystal wafers have been synthesized by the “confined space” growth method (**Figure** **20**a).^[^ ^341^, ^342^, ^343^ ^]^ Briefly, two glass substrates with a confined space are immersed in the precursor solution for perovskite crystal growth. A nucleation seed can be placed between the glass to trigger crystal growth within the confined space. As a result, flat crystals with controlled thicknesses from 10s to 100s of µm can be achieved with a large lateral dimension (over 1 cm). Multi‐pixel devices can be built on the flat crystals for imaging applications (Figure 20b). Peng et al. have also demonstrated a sonication‐assisted thin crystal growth method which utilizes an ultra‐sonication pulse to initiate the nucleation for the thin crystal film growth.^[^ ^344^ ^]^ With this method, a thin MAPbBr_3_ single crystal film was obtained that was demonstrated for visible light sensing with 100% external quantum efficiencies.

Polycrystalline thick films have also been developed. A notable advantage of polycrystalline perovskites over single crystals is their superior flexibility, which allows them to be used in a variety of wearable X‐ray detector systems owing to their ability to adhere to uneven surfaces, as well as their lightweight nature. Sintering, isostatic‐pressing, spin‐coating, printing, spraying, and hot‐casting methods can be used to grow large‐area polycrystalline perovskites with large granules.^[^ ^105^ ^]^ The size of the film can reach up to 50 × 50 cm^2^ which is larger than the standard size of commercial digital radiography detectors (1000 cm^2^). Although polycrystalline films obtained by solution processing are known for exhibiting excellent figures‐of‐merit in radiation detection, their electronic properties are typically reduced due to the presence of grain boundaries and pinholes. These pinholes and grain boundaries can trap electrons and holes, leading to increased non‐radiative recombination, slowing charge‐carrier collection, and resulting in poor image resolution. Furthermore, the presence of pinholes in the perovskite active layer can decrease the film density and lead to a higher concentration of defects and shunt pathways, which result in low X‐ray absorption and a large dark current. A thicker polycrystalline perovskite layer is required to sufficiently attenuate the high‐energy photons, in turn, the carrier collection efficiency will be reduced. The fabrication of high‐quality polycrystalline perovskites with submillimeter‐scale thickness still remains an unexplored topic that must be tackled meticulously for optimal device performance.

### 5.5 Integration to Circuits for Read‐Out

Although much of the literature thus far has focused on simple test‐scale devices, the ultimate commercial use of perovskite direct detectors requires their integration with a specific readout integrated circuit for practical applications (i.e., X‐ray imaging, single‐photon emission computed tomography, and particle detection). Beyond the perovskite thick film deposition approaches described in Section 4.8, it is important to consider how the active layer is coupled to the readout circuit, including electrode deposition and bonding. Common electrical integration methods including wire bonding, flip‐chip bonding, and anisotropic conductive film bonding. These processes involve heating and pressing, which may damage the soft perovskite layer. In contrast, monolithically growing perovskite polycrystalline films or single crystals onto thin‐film transistors or complementary metal oxide semiconductor (CMOS) arrays is an effective method for device integration. Yong et al. first reported the integration of MAPbI_3_ polycrystalline film on thin‐film transistor pixels by the paste doctor blading technique.^[^ ^81^ ^]^ Polyimide (PI)‐perovskite composites were inserted to reduce the dark current and enhance adhesion between the perovskite film and transistor backplane. A good sensitivity of 11 000 µC Gy_air_^−1^ cm^−2^ with an imaging spatial resolution of 3.1 lp mm^−1^ was obtained under irradiation with a 100 kV bremsstrahlung source, which is 10 times higher than α‐Se direct detectors or Tl:CsI scintillators.^[^ ^81^ ^]^ Huang et al. directly grew a perovskite single crystal on a Si substrate through a solid mechanical and electrical connection using a dual linker.^[^ ^134^ ^]^ Brominated (3‐aminopropyl) triethoxysilane molecule bonds to silicon at native oxide sites and contributes to the chemical structure of perovskite crystal via ammonium bromide groups. The dipole properties of the linker not only retained the signal intensity of the devices, but also reduced its noise. As a result, the fabricated devices exhibited a high sensitivity of 2.1 × 10^4^ µC Gy_air_^−1^ cm^−2^ under 8 keV X‐ray radiation. The resolution was approximately 10 lp mm^−1^ at 20% modulation transfer function, which is more than twice as large as the spatial resolution of the most recently reported polycrystalline perovskite and hybrid organic and X‐ray detectors.^[^ ^345^ ^]^ The evolution in spatial resolution for perovskite radiation detector‐based X‐ray imaging is summarized in **Table** **4**. Currently, the available ASICs (application‐specific integrated circuits) designed to fit other materials like CdTe and CdZnTe might not be directly suited for perovskites owing to the mismatched amplifier. Appropriate signal readout circuits must be developed for perovskite radiation detector‐based X‐ray imaging panels or γ‐ray cameras.

**Table 4:** Evolution in the spatial resolution of perovskite radiation detectors for X‐ray imaging

Detection mode	Material	Crystal form	Mobility lifetime (µτ) [cm^2^ V^–1^]	Sensitivity (µC Gy_air_^−1^cm^−1^)	Detection limit [nGy_air_ s^−1^]	Spatial resolution [lp mm^−1^]	Reference
Semi‐conductor (direct)	MAPbI_3_ wafers	polycrystals	4 × 10^–4^	9,300	0.22	6.0	
	Polyimid‐MAPbI_3_	polycrystals	1 × 10^−7^	11,000	–	3.1	
	MAPbBr_3_	single crystals	–	21,000	0.1	10.0	
	MAPbI_3_	polycrystals	1 × 10^–4^	11	–	–	[81]
	MAPb(I_0.9_Cl_0.1_)_3_	polycrystals	1 .5 × 10^–3^	8696	–	3.5
	δ‐CsPbI_3_	microwires		190	33.3	12.4
	Cs_2_AgBiBr_6_	polycrystal wafer	1 .57 × 10^–3^	250	95.3	4.9
	MA_3_Bi_2_I_9_	single crystals		872	31	4.22
Scintillator (indirect)	CsPbBr_3_	nanocrystals	–	–	13.0	5.0	
	CsPbBr_3_	nanowires	–	–	–	250.0	
	CsPbBr_3_	quantum dots	–	–	33.0	250.0
	CsPbBr_3_	nanocrystals	–	–	–	9.8	
	Cs_2_Ag_0.6_Na_0.4_In_0.85_Bi_0.15_Cl_6_	polycrystalline	–	–	19	4.3	

It should be noted that the spatial resolution of direct conversion (semiconductor) type devices is not directly comparable with that of indirect conversion (scintillator‐based) devices. The factors that limit the spatial resolution of these two types of detection mechanisms differ significantly. For indirect detectors, the spatial resolution is limited by the attenuation coefficient, light yield, K‐shell X‐ray re‐absorption,^[^ ^346^ ^]^ the spread of visible light during propagation,^[^ ^347^ ^]^ and visible photon readout efficiency. On the other hand, the spatial resolution of direct conversion detectors is determined by the detective conversion efficiency, read‐out circuit used, charge trapping, and inter‐pixel cross‐talk effects.^[^ ^69^ ^]^

## 6 Outlook

As can be seen from the discussion in this Review, metal–halide semiconductors have reinvigorated the development of cost‐effective, high‐performance radiation detectors that can be used for a wide variety of applications. Future work should prioritize the development of more stable semiconductors with high mobility‐lifetime products and low polarization, as well as tackle the challenges of translating the exceptional performance achieved thus‐far in small single crystals to large‐area detectors for practical applications. Here, we discuss our views on important current challenges to address to advance this growing field.

### 6.1 Future Directions with Lead‐Free Perovskite‐Inspired Materials

Bi‐based PIMs have already demonstrated highly‐promising performance, with some of the lowest LoDDs out of all materials (down to a directly measured value of 1.2 nGy_air_ s^−1^),^[^ ^146^ ^]^ lower ion migration than 3D LHPs, stable dark currents, and high radiation hardness to X‐rays and γ‐rays. There have been promising initial reports of these materials as polycrystalline wafers, which have the potential for scaling up the detectors for medical imaging applications. Spatial resolutions of 4.9 lp mm^−1^ have been achieved from BiOBr‐passivated Cs_2_AgBiBr_6_ wafers,^[^ ^78^ ^]^ which is nearly adequate for radiology (5.7 lp mm^−1^ needed), but falls below the minimum required resolution for mammography (10 lp mm^−1^).^[^ ^53^ ^]^ Further work is therefore needed to firstly develop more of the highly‐promising Bi‐based PIMs into imagers, and secondly to optimize the processing of these materials to simultaneously improve charge‐collection efficiencies while minimizing dark current densities to increase the imaging resolution. This will require understanding and mitigating the roles of grain boundaries, while reducing charge‐carrier scattering at grain boundaries or other structural defects, such as through heteroepitaxial passivation or annealing to promote grain growth.

It will also be important to explore more widely approaches to grow Bi‐based PIMs over large area. Apart from isostatic pressing of powders, thick film growth should also be explored, particularly if films can be grown rapidly at low temperature, making them suited to direct integration with Si‐based TFT arrays for imagers. But since many Bi‐based PIMs have low dimensionality, it will be important to control the preferred orientation of grains in wafers or thick films.

Beyond X‐ray detection, it will be important to make use of the potential of Bi‐based PIMs for γ‐ray detection, as well as other sources of ionizing radiation, such as α‐particles. Initial reports of Cs_2_AgBiBr_6_ and Cs_3_Bi_2_I_9_ for γ‐ray detection only achieved energy resolutions of 13.9% (for 59.6 keV ^241^Am)^[^ ^352^ ^]^ and 32% (for 5.5 MeV ^241^Am),^[^ ^353^ ^]^ respectively. However, the crystals investigated did not have state‐of‐the‐art *µτ* products, and there are Bi‐based PIMs with much higher *Z*_eff_ values than Cs_2_AgBiBr_6_. Further efforts in this area are therefore likely to yield much improved results. It will especially be important to select materials that can be grown as single crystals to the mm scale in order to have a sufficiently high attenuation efficiency. Single crystals are especially well‐suited to this application by eliminating grain boundaries, since only a small quantity of charge‐carriers are generated.

Finally, there is a rich opportunity to design materials in this space that reduces coupling to both optical and acoustic phonons. As discussed in Section 3.2.5, carrier localization has been widely found in Bi‐based PIMs with low electronic dimensionality, which reduces charge‐carrier mobilities. The recent work by us and others with BiOI shows that this could be avoided, to achieve fully delocalized excitons following photoexcitation. The next important step will be to explore more materials systems in depth to gain a greater understanding of why this occurs, and whether there are chemical or structural descriptors that could be used to predict materials that can maintain delocalized excitations. Furthermore, it will be important to design materials that can reduce the strength of Fröhlich coupling and achieve large mobility values. Success in this area will not only lead to more effective radiation detectors, but also develop materials that could make efficient diffusion‐driven devices as well, such as indoor photovoltaics or photoelectrochemical cells, which have similar bandgap requirements.

### 6.2 Interface Engineering and Future Work on Heterostructures

Surface defects are common imperfections that can weaken the electrical and thermal properties of perovskite materials. Interface engineering can modulate the device's stability. Several efforts have been made for the passivation of grain boundaries, as elaborated on in Section 4.3. Interface engineering by using a variety of charge transport layers has been shown to increase the stability of perovskite solar cells against oxygen degradation and water,^[^ ^354^ ^]^ which can be borrowed for improving X‐ray detectors. A recent study explored the use of Zr, Bi, Ti, and Ga as metal contacts on CsPbBr_3_ single‐crystal γ‐ray detectors.^[^ ^355^ ^]^ However, the interface stabilities remain uncertain and further efforts on building reliable contacts, including metallic or carbon electrodes or interface barriers to block the ion inter‐diffusion, will be valuable.

Sections 4.5 and 4.6 provided a few examples of the heterostructures explored thus far that have enabled improved charge‐carrier transport and device stability. 2D/3D structures can stabilize the interface and guide charge flow. Heterostructures with two different compositions can create *p*‐*n* junctions with built‐in fields to enhance charge separation. Building on these successes, more heterostructures can be explored for improving charge‐collection efficiencies. Interfaces grown by two perovskites with similar lattice parameters in a superstructure provide a clean channel for charge flow without scattering. Therefore, the choice of materials should be considered carefully to maximize the built‐in electric field and minimize lattice mismatch at the interface for better integration. Double and multiple heterostructure single crystal perovskites were introduced by several groups.^[^ ^278^ ^]^ Heterostructured perovskite single crystals exhibit remarkable stability against various degradation factors and also demonstrate high performance, for example with solar cells based on lead‐tin gradient structures that achieved an average efficiency of 18.77%.^[^ ^340^ ^]^ Despite the potential of heterostructure perovskite single crystals for X‐ray or γ‐ray detection, there have been limited reported uses in this field, suggesting a need for further work. Given the large structural tunability space in the perovskites, the possibility of building lattice‐matched or lattice‐mis‐matched heterostructures is just open. For instance, exploring the fabrication of heterostructure bismuth‐halide perovskite single crystals could be a promising avenue for developing high‐performance radiation detectors for future commercialization. Section 4.6 provides an example of building 2D over 3D double perovskites with atomically sharp interfaces. Such a sharp interface delivered a high built‐in field. More clean interfaces like this example should be explored to further increase the built‐in field strength or widen the depletion width of the junction. Fundamental understanding of the interface growth kinetics that governs the interface charge transport properties is also necessary. Given the large variety of material combinations, screening through the material growth space would be impossible. Therefore, including automated experiment coupled with data science and machine learning that can predict the best interface for high‐performance X‐ray detection could be advantageous.

### 6.3 Standardization of Measurements and Suggestions on Best Practices in Reporting

Currently, there is a lack of standard approaches for testing and reporting the performance of novel direct radiation detectors. Unlike photovoltaic devices where all solar cells are tested under a standard light source with protocols for reporting and verification, perovskite radiation detectors are currently tested under a variety of conditions, using a wide variety of approaches. This makes it difficult to directly compare the performances reported by different groups. Standardization is becoming increasingly important as the perovskite radiation detectors field grows.

#### 6.3.1 Sensitivity Quantification

The X‐ray energies employed for detector testing span from 8 keV to 100 keV (or higher). Since the number of ionized charge pairs depends on the X‐ray photon energy, the resulting sensitivity for a given thickness could drastically change for the same detector. In addition, the detector's operational conditions also alter the resulting sensitivities obtained. As described in Section 2, photoconductive gain can occur at high electrical fields when the carrier recombination lifetime is longer than their transport time. It is noted that some papers report sensitivities measured at low bias, while other studies report the sensitivity obtained under high fields. As discussed in Section 2.2.1, Cao et al. demonstrated that the X‐ray sensitivity can vary by 2 orders of magnitude when a perovskite diode is operating under forward or reverse bias because of the different charge injection from the electrode (Figure 4e). It is known that charge injection from the non‐blocking contact can effectively multiply the photo‐carrier causing a gain in sensitivity (or photoconductive gain). Such an effect can lead to an infinitely high sensitivity number, which according to a recent review article by He et al., is responsible for the high sensitivity obtained in the recently reported perovskite detectors.^[^ ^119^ ^]^ Sensitivity values can also be affected by the dose rate range used for the measurements causing a non‐linear dependence in the charge‐dose rate plot. This could occur when operating the detector under a low intensity where charge trapping or charge injection‐induced sensitivity gain is significant. Furthermore, under the high‐density beam, charge recombination could dominate and decrease the electrical pulse signal.

The presence of photoconductive gain makes it difficult to compare the performance of detectors across the literature because of the significant variation in gain, which, in many cases, are not quantified and reported. It will therefore be helpful to conduct a back‐of‐the‐envelope calculation of the sensitivity value measured from the detector.^[^ ^121^ ^]^ This can be done by calculating the total energy of X‐ray photons absorbed throughout the thickness of the detector material, and the number of charges ionized using the empirical relation of the ionization energy (*W*) given in Equation 11.

(11) $$W\;=\frac{E_{T}\;}{n_{e-h}}\;$$

In Equation 11, *n*_e − h_ is the total number of electron‐hole pairs created, and *E*_T_ is the total photon energy absorbed, which is given by *E*_T_ = (photon density) × (photon energy). η is the device absorption efficiency, which is given by Equation 12.

(12) $$\eta \;=\;1-e^{-\mu_{\mathrm{la}}d}$$

In Equation 12, µ_la_ is the linear attenuation coefficient and *d* is the thickness of the absorber. Overall, Equation 11 describes the energy required to ionize an electron, and is usually three times the semiconductor's optical bandgap (see Equation 1 in Section 2.2). Since *n*_e − h_is known from the detector's sensitivity measurements, assuming a monochromatic energy from the X‐ray's characteristic peak, *W* can be calculated. If *W* is significantly higher than 3*E*_g_, it suggests that not all ionized charges are collected, and if *W* is significantly smaller than the 3*E*_g_, the number of collected charges are likely more than the ionized charges, which could be attributed to a gain.

On more practical terms, photoconductive gain is a beneficial phenomenon to boost the sensing efficiency especially for low flux sensing. At the same time, photoconductive gain can result in an increase in the dark current, and increase the noise. Therefore, an analysis of the signal to noise ratio would be beneficial to evaluate whether photoconductive gain leads to an increase in sensitivity that outweighs the decrease in SNR.

#### 6.3.2 Beam Calibration

To calibrate a beam, a detector with known characteristics can be used. Air kerma (*K*_air_) is a quantity describing the energy per unit mass of a beam of photons or particles that are transferred to the material (units: Gy). Another quantity called exposure (*X*) is also employed for beam characteristics (units: C kg^−1^). *X* is usually used for characterizing the charge liberated through the ionization of dry air. The conversion between *K*_air_ and *X* is given by Equation 13.

(13) $$X\;=K_{air}\;\frac{1}{2.58E-4}\frac{1}{W_{air}/q}$$

*W*_air_/*q* is a constant (33.97 eV/ion pair) for dry air.

Dosimetry used for X‐ray beam calibration includes silicon photodiodes, ion chambers, or Geiger–Müller counters. Silicon photodiodes are solid‐state detectors and should be pre‐calibrated at each X‐ray energy. The Geiger‐Müller counter is not suitable for high‐dose calibration because signal pile‐up will interfere with the results. The most practical and commonly used dosimeter is the ion chamber, which contains a fixed volume of gas, typically dry air, that can be ionized by the radiation photons. The subsequent charge signal (*Q*) collected by the chamber's electrodes corresponds to the dose (*D*) of the incoming photon, as given by Equation 14.

(14) $$D\;=\frac{Q}{m_{air}}\;\left(\frac{W_{air}}{q}\right)$$

Here, the mass of the air (*m*_air_) in the ion chamber is known.

As suggested by Andreo et al., *W*_air_/*q* could change by 0.6%, and is affected by the relative humidity, operational temperature, and pressure.^[^ ^356^ ^]^ A precise dose of radiation should be delivered to the patient for an accurate and safe treatment. Therefore, the calibration protocols for photon sources used in hospitals have been rigorously kept with a high level of consistency following guidance from national or regional organizations. The work by Pan et al. described a variety of beam calibration methods, such as using a silicon photo‐diode to measure the photon flux, and an ion chamber to directly probe the dose rates.^[^ ^78^ ^]^ They found a good consistency of the calibrated values from both methods.

Another factor to consider is the background portion of the X‐ray beam. Although the characteristic peak energy of an X‐ray source is usually reported, its background counts over the full spectrum should not be ignored. This is especially the case for thin‐film detectors in which the high energy characteristic peak could not be efficiently attenuated, and the detector would then show a non‐negligible response to the low‐energy background portion of the incident spectrum. Therefore, the specific X‐ray energies used for detector characterization should be pre‐selected based on the attenuation and material thicknesses. If the energy is too low for a thick perovskite detector, all energies will deposit near the interface of the crystal where charges are ionized non‐uniformly in the device. As a consequence, significant losses could be expected. On the other hand, high‐energy X‐ray beams (e.g., from W tubes) is not suitable to characterize a thin perovskite detector, since the peak X‐ray photon will penetrate through the detector without ionizing charges. The lower‐energy X‐ray generated from the background will likely dominate the charge‐generation process. It is thus difficult to differentiate the signal generated from the characteristic peak of the beam or from its background.

Considering the above discussion, the beam characteristics should also be included in publications. This includes the beam energy spectrum, the characteristic peaks, and the background signals from the X‐ray tube. In addition, the dose from an X‐ray tube could have transient behavior. For example, once turned on, it will first produce a high spike of dose followed by a steady state dose. This should be taken into consideration when calibrating the dose rate, and the detector should be measured after the beam flux is stabilized.

#### 6.3.3 Lowest Detectable Dose Rate

**Figure 21:**
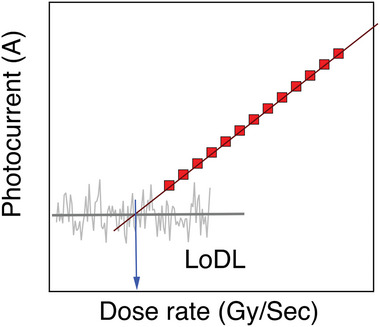
An example of the linear regression curve and the method to find the LoDD (or LoDL). Red squares are the acquired photo‐induced current from a detector, and the gray lines are the noise from the photo‐current.

As pointed out in a recent paper by Cao et al., the LoDDs and sensitivities are not always reported at the same time, or under the same measurement conditions.^[^ ^43^ ^]^ Only reporting the sensitivity might not be meaningful because it can vary significantly based on the testing conditions and the degree of photoconductive gain present. Reporting the LoDDs would therefore be important, but there is no standard protocol for measuring the detection limit between different groups, and, as discussed in Section 2.2.2, the detection limit obtained can vary depending on the measurement conditions. As illustrated in **Figure** **21**, the extracted current from a detector usually scales linearly with the exposure dose rate, and would cut off once it approaches the dark current. The LoDD can thus be estimated by the intersection of the dark current and the extrapolated linear‐regression curve of the photocurrent. However, the fluctuation in the noise introduces a complication. As shown in Figure 21, the intersection is difficult to find as the noise increases. Many groups extract their LoDD values following a 1975 International Union of Pure and Applied Chemistry (IUPAC) detection limit definition, where the SNR must be greater than 3 when determining the LoDD.^[^ ^79^, ^94^, ^96^, ^136^ ^]^ But the methods to quantify the noise are not unified to be able to compare the different values reported.

Cao et al. established a detailed calculation method for the LoDD. This method accounts for the dark noise and photocurrent noise for an accurate extraction of the ionized charge density, and uses the dark current noise to determine the detection limit. They found a large difference when different noises were utilized, and the noise itself is dependent on the applied bias. This comprehensive study hints at the large uncertainty when extracting the LoDD.^[^ ^43^ ^]^

#### 6.3.4 Toward a More Consistent Approach to Stability Testing and Reporting

In the perovskite photovoltaic community, there have been many discussions and protocols written on measuring and reporting a solar cell's operational lifetime. As with sensitivity measurements, there is no unified approach to measuring and reporting stability. For example, from the discussion in Section 3, it can be seen that each different group uses different dose rates and measures the performance for different lengths of time, without any consistency in the applied bias used. The lack of protocols is partly due to the greater number of variables for detector testing, such as photon energies (which vary over a wide range for different applications, as shown in Table 1), photon counting vs integrated current sensing modes, as well the wider range of parameters that need to be consistent with time (sensitivity, LoDD, dark current, phase). It is therefore difficult to establish a general stability testing protocol. However, we believe that a few guidelines can be presented here to initiate discussions on this important topic.

The dosage used for stability tests could be specified based on their target applications. For instance, a typical CT scan delivers a total dose of 5 ≈ 30 mSv,^[^ ^357^ ^]^ corresponding to 5 mGy for each treatment. For detectors operating in near‐Earth orbit, the total radiation dose received by the device is as high as 10000 rad each year (or 1000 Gy each year).^[^ ^358^ ^]^ A detector serving in a multi‐year mission will be exposed to significantly larger total irradiation. Therefore, evaluating the detector's robustness after receiving the total dose that matches the specific application would be useful.

Other reliability tests should be developed for perovskite radiation detectors. In the reliability test protocols for photovoltaic panels, thermal stress, and mechanical tests are usually included to understand the robustness under unforeseen operational conditions. Similarly for radiation detectors, how the detector's performance changes under cold and hot conditions should be evaluated under more extreme environments such as outer space or nuclear reactors. Possible degradation mechanisms, such as temperature‐induced performance decreases or structural decomposition, should be investigated. Mechanical stresses should also be accounted for to evaluate the reliability of the perovskite detectors. Interface delamination or material breakdown could occur upon the application of mechanical stress that can significantly impact the operational lifetime of the detectors. In addition, after radiation bombardment, the properties at interface properties could be altered, which can make the devices more vulnerable to degradation.

## 7 Conclusion

Metal–halide perovskites and their derivatives have led to a renaissance in the ionizing radiation detector field. A wide variety of materials have been developed over the past decade that overcome the limitations of incumbent direct detectors by giving rise to orders of magnitude higher sensitivities, lower detection limits, and improved cost‐effectiveness. These rapid rises in performance have come about because of the high stopping power of these materials, large mobility‐lifetime products, and the amenability of these materials to be grown by a variety of solution‐ and vapor‐based synthesis methods as high‐quality single crystals with low defect densities and high resistivities. In particular, Bi‐based derivatives, which have underperformed in solar cells, have recently emerged as highly promising radiation detector materials. Not only have these materials come to match the low LoDDs achieved in lead‐halide perovskites (down to ≈1 nGy_air_ s^−1^), they overcome some of the limitations of LHPs, namely in terms of toxicity, air stability, and polarization stability. Many factors can be utilized to optimize the performance of both PIMs and LHPs, from the structural dimensionality and form factor, through to the formation of heterostructures.

Bringing these materials to the market will require several challenges to be addressed. Firstly, these materials need to be grown over large area, while maintaining the high performance of the single crystals. Highly promising results have been achieved in polycrystalline wafers and thick films of selected materials, but further work is required to improve their spatial resolution to exceed those of incumbent CZT detectors and fulfill the requirements of digital radiography, as well as the stricter requirements of mammography. Secondly, the environmental, thermal, and polarization stability of these detectors needs to be improved, such as by making use of effective encapsulation, composites, and heterostructures to passivate surfaces. Thirdly, urgent efforts are needed for the field to reach a consensus on protocols for the measurement and reporting of X‐ray and γ‐ray detector performance, particularly accounting for the effects of photoconductive gain, in order to truly pin down the status of the field as it grows and advances. Finally, more efforts are needed to take advantage of the opportunities these materials offer in terms of flexible and wearable medical imaging detectors, as well as high‐resolution room temperature particle detectors, not just for γ‐rays, but also for α‐particles, β‐particles, and many more. Taking advantage of these opportunities will lead to many societal benefits, from safer medical imaging through to more effective detectors for homeland security, particle physics research and astronomy.

## Conflict of Interest

The authors declare no conflict of interest.
